# Mining the Human Phenome Using Allelic Scores That Index Biological Intermediates

**DOI:** 10.1371/journal.pgen.1003919

**Published:** 2013-10-31

**Authors:** David M. Evans, Marie Jo A. Brion, Lavinia Paternoster, John P. Kemp, George McMahon, Marcus Munafò, John B. Whitfield, Sarah E. Medland, Grant W. Montgomery, The GIANT consortium, The CRP consortium, The TAG Consortium, Nicholas J. Timpson, Beate St. Pourcain, Debbie A. Lawlor, Nicholas G. Martin, Abbas Dehghan, Joel Hirschhorn, George Davey Smith

**Affiliations:** 1 MRC Integrative Epidemiology Unit, University of Bristol, Bristol, United Kingdom; 2 School of Social and Community Medicine, University of Bristol, Bristol, United Kingdom; 3 University of Queensland Diamantina Institute, Translational Research Institute, Brisbane, Queensland, Australia; 4 Broad Institute at MIT and Harvard, Cambridge, Massachusetts, United States of America; 5 Queensland Brain Institute, University of Queensland, Brisbane, Australia; 6 UK Centre for Tobacco and Alcohol Studies, School of Experimental Psychology, University of Bristol, Bristol, United Kingdom; 7 QIMR Berghofer Medical Research Institute, Brisbane, Australia; 8 Department of Epidemiology, Erasmus Medical Centre, Rotterdam, The Netherlands; 9 Division of Genetics and Endocrinology and Program in Genomics, Children's Hospital, Boston, Massachusetts, United States of America; 10 Department of Genetics, Harvard Medical School, Boston, Massachusetts; National Institute of Genetics, Japan

## Abstract

It is common practice in genome-wide association studies (GWAS) to focus on the relationship between disease risk and genetic variants one marker at a time. When relevant genes are identified it is often possible to implicate biological intermediates and pathways likely to be involved in disease aetiology. However, single genetic variants typically explain small amounts of disease risk. Our idea is to construct allelic scores that explain greater proportions of the variance in biological intermediates, and subsequently use these scores to data mine GWAS. To investigate the approach's properties, we indexed three biological intermediates where the results of large GWAS meta-analyses were available: body mass index, C-reactive protein and low density lipoprotein levels. We generated allelic scores in the Avon Longitudinal Study of Parents and Children, and in publicly available data from the first Wellcome Trust Case Control Consortium. We compared the explanatory ability of allelic scores in terms of their capacity to proxy for the intermediate of interest, and the extent to which they associated with disease. We found that allelic scores derived from known variants and allelic scores derived from hundreds of thousands of genetic markers explained significant portions of the variance in biological intermediates of interest, and many of these scores showed expected correlations with disease. Genome-wide allelic scores however tended to lack specificity suggesting that they should be used with caution and perhaps only to proxy biological intermediates for which there are no known individual variants. Power calculations confirm the feasibility of extending our strategy to the analysis of tens of thousands of molecular phenotypes in large genome-wide meta-analyses. We conclude that our method represents a simple way in which potentially tens of thousands of molecular phenotypes could be screened for causal relationships with disease without having to expensively measure these variables in individual disease collections.

## Author Summary

The standard approach in genome-wide association studies is to analyse the relationship between genetic variants and disease one marker at a time. Significant associations between markers and disease are then used as evidence to implicate biological intermediates and pathways likely to be involved in disease aetiology. However, single genetic variants typically only explain small amounts of disease risk. Our idea is to construct allelic scores that explain greater proportions of the variance in biological intermediates than single markers, and then use these scores to data mine genome-wide association studies. We show how allelic scores derived from known variants as well as allelic scores derived from hundreds of thousands of genetic markers across the genome explain significant portions of the variance in body mass index, levels of C-reactive protein, and LDLc cholesterol, and many of these scores show expected correlations with disease. Power calculations confirm the feasibility of scaling our strategy to the analysis of tens of thousands of molecular phenotypes in large genome-wide meta-analyses. Our method represents a simple way in which tens of thousands of molecular phenotypes could be screened for potential causal relationships with disease.

## Introduction

It is common practice within genome-wide association studies (GWAS) and their meta-analyses to focus on the relationship between disease risk and single nucleotide polymorphisms (SNPs) one genetic variant at a time. This strategy is often very informative in terms of identifying biological intermediates and/or pathways likely to be important in disease pathogenesis. For example, the association between coronary heart disease and genetic variants located within genes regulating levels of low density lipoprotein (LDLc) [1]–[4], confirms low density cholesterol as a key player in the aetiology of coronary heart disease. Likewise, it is now common practice to follow up disease associated variants in gene expression studies. If the disease associated variant is also related to levels of gene expression in a relevant target tissue, then this is often interpreted as prima facie evidence that the variant exerts at least part of its functional effect by altering transcription levels of that gene, which downstream subsequently predisposes to disease. Using a similar rationale, the absence of genetic association can also be informative in providing evidence against biological intermediates playing a role in disease aetiology so long as the study is adequately powered. For example, Mendelian Randomization [5] studies have shown that variants within the CRP gene appear to be unrelated to hypertension, type 2 diabetes and coronary heart disease, suggesting that CRP is unlikely to be important in the aetiology of these conditions, but rather that observational associations between CRP and these diseases are more likely to represent confounding and/or reverse causation [6]–[8].

Given that genetic variants can highlight potentially important relationships between biological mediators/environmental exposures and disease, it would seem a worthwhile exercise to screen GWAS of as many diseases as possible for SNPs known to be related to biological intermediates. However single variants typically explain only a small proportion of the variance in these biological intermediates, and so it might be expected that the SNPs indexing these variables, may not show strong evidence of association, particularly in smaller GWAS. Potentially, a more powerful strategy would be to look at the combined effect of several genetic variants that together explain greater variance in the intermediate of interest, and consequently may be more strongly related to disease. In other words, our idea is to invert the GWAS paradigm. Rather than investigate SNPs which are associated with disease and then see if they are related to intermediates, take combinations of SNPs known to be related to biological intermediates and test to see if they are related to disease.

One might ask the obvious question, if the interest is on the relationship between biological intermediates and disease, then why not measure these quantities directly in the observational studies themselves? Whilst this is certainly possible, and may have many benefits, we argue that our strategy has several advantages that make it a worthwhile approach to consider. First, our method provides a way to efficiently screen many different biological intermediates quickly and inexpensively without having to measure them in the disease cohort of interest. All that is required is knowledge of the genetic variants that relate to the biological intermediate of interest and that these same SNPs have been genotyped on a sample of disease cases and controls (in practice this will most likely mean using GWAS data). An added benefit is that due to the existence of GWAS consortia, the strategy could in theory be applied to the tens of thousands of individuals that have been genotyped as part of these consortia making the method potentially very powerful.

Second, the same allelic scores could be used to screen an unlimited number of different collections and/or diseases so long as (genome-wide) SNP data is available in these cohorts and includes variants related to the intermediates of interest. Third, allelic scores are more likely to represent individuals' lifetime exposure to the factor of interest rather than a one off measurement of the intermediate, which in contrast, might be susceptible to considerable measurement error and time dependency [2]. Fourth, whilst measurements of biological intermediates in disease populations may be influenced by medications and/or reverse causality, we expect that genetic variants/allele scores are not influenced by many confounders (including medications and/or reverse causality), although we stress that even in this case, correlation does not necessarily imply causation.

Finally, and most importantly, the approach is in theory extendable to any variable of interest, not just single biological intermediates, but potentially multiple molecular phenotypes as well (e.g. levels of gene expression, methylation, metabolomic data etc). This means that in principle tens of thousands of molecular phenotypes could be screened simultaneously for possible causal relationships with the disease of interest, and in so doing flag biological pathways that deserve attention. These associations could then be followed up in more detail e.g. by formal Mendelian Randomization to investigate the possibility of a causal relationship further [5]. We emphasize, however, that the approach will not identify observational associations which are due to environmental factors which affect both the intermediate and disease, nor will it identify associations which are due to the disease causing the intermediate (i.e. reverse causality). This is advantageous if one is only interested in factors which potentially cause disease, but will also by definition exclude non-causal associations which could potentially be of utility such as non-causal biomarkers. For example, assuming that elevated levels of CRP is not a contributing causal factor for coronary heart disease [8], then genetic variants which index CRP, should not be related to coronary heart disease, even though levels of CRP may serve as a useful biomarker of disease risk.

One obvious limitation of what we have proposed so far is that the genetic variants related to the biological intermediate need to be known *a priori* in order for the approach to work. In addition, in the case of intermediates where known variants exist, they may explain only a small amount of the total phenotypic variance in that variable. However, we and others have previously shown that genome-wide allelic scores generated by simply counting up hundreds of thousands of anonymous “risk” alleles in genome-wide SNP data are capable of explaining meaningful amounts of phenotypic variance in traits of interest [9], [10]. Our idea is to use these genome-wide allelic scores in situations where there are no known confirmed genetic variants and/or in situations where the known variants explain inadequate proportions of the phenotypic variance in the biological intermediates of interest. In fact, our previous work has shown that these scores can explain more phenotypic variance than allelic scores constructed from confirmed variants only [9], [10]. This is because many complex phenotypes (including biological intermediates) are influenced by hundreds, if not thousands of common variants of small effect scattered across the genome [11], [12]. There is thus considerable information in the lower part of the genome-wide distribution of association test statistics that could be utilized to explain more of the phenotypic variance in the modifiable exposures of interest (i.e. SNPs that exhibit p values>5×10^−8^ which do not meet the stringent criterion for genome-wide significance also provide important predictive information).

In this manuscript we investigate the possibility of using allelic scores that index biological intermediates as a method of screening for potentially causal associations between these variables and disease. We begin first by investigating the ability of allelic scores to explain variance in modifiable exposures/biological intermediates of interest in a large population based cohort- the Avon Longitudinal Study of Parents and Children (ALSPAC). We compare the explanatory ability of allelic scores constructed from confirmed variants only, to genome-wide allelic scores generated from up to hundreds of thousands of anonymous SNPs. In order to investigate the properties of our approach, we attempt to index three biological intermediates of interest where the results of large GWAS meta-analyses are available: body mass index (BMI), C-reactive protein (CRP) and LDLc [3], [13], [14]. In order to replicate our pattern of associations, we perform the same set of analyses in an independent cohort of Australian twins (QIMR Twins) [15], [16]. We subsequently generate these allelic scores which index BMI, CRP and LDLc in publicly available data from the first Wellcome Trust Case Control Consortium [17], and investigate the extent to which the scores are associated with case control status across seven common diseases (Bipolar disorder, Coronary Artery Disease, Crohn's Disease, Hypertension, Rheumatoid Arthritis, Type I Diabetes, Type II Diabetes).

For several of the intermediate variable - disease pairings there exists strong evidence of a causal relationship between the two e.g. from randomized controlled trials, Mendelian Randomization studies etc. These include LDLc with coronary heart disease [1], [18], and BMI and both coronary heart disease [19] and type 2 diabetes [20], [21]. We therefore expect that at least some of these allelic scores indexing biological intermediates will show association with disease, even in a relatively small sample like the WTCCC. In contrast, for other pairings, even though observational research has shown that the two variables are related, the pairing is unlikely to reflect a causal effect of the intermediate/exposure variable on the disease (e.g. CRP and type 2 diabetes [6] or coronary heart disease [8]), and thus we expect that allelic scores should not show correlation with disease in these cases. If we can show that the approach produces coherent results in situations where we are relatively confident of a causal relationship between the intermediate and disease, then the implication is that the method may also be useful in those situations where we are less certain of the underlying relationship between the variables, such as in a screen of tens of thousands of molecular phenotypes. Finally we investigate the power of the approach, and discuss the likely challenges involved in scaling the strategy up to investigate tens of thousands of molecular phenotypes.

## Results

### Performance of allelic scores in the ALSPAC cohort

**Figure 1:**
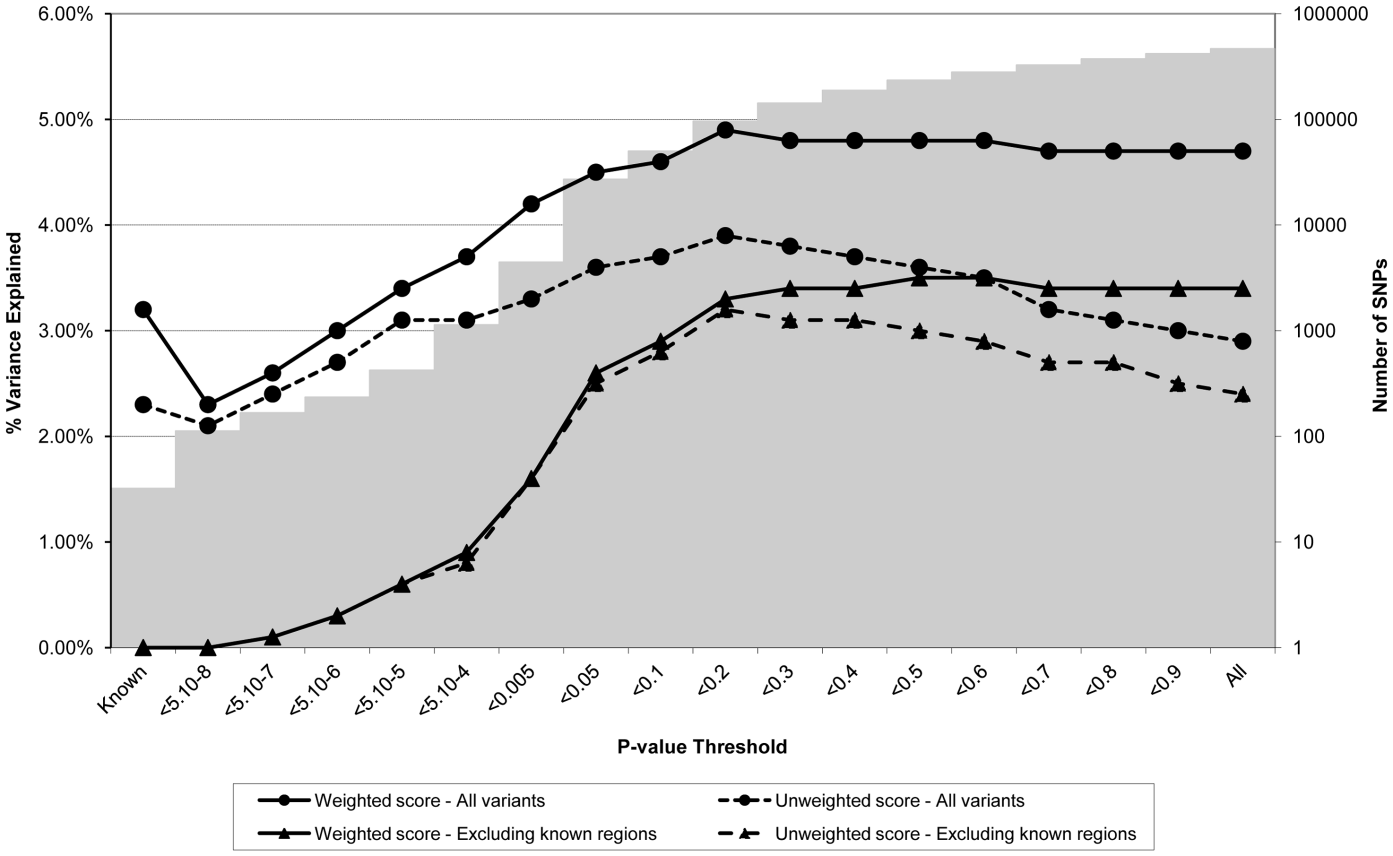
Association between polygene score and BMI measured at age nine in the ALSPAC cohort. Association between polygene score and BMI measured at age nine using different p-value thresholds for the construction of the score in ALSPAC children (N = 5819). The lines joining the circles display the results for allelic scores calculated by using genotyped variants from across the genome in either a weighted (unbroken line) or an unweighted (dashed line) fashion. The lines joining the triangles display scores calculated similarly but excluding all variants +/−1 MB around 32 known BMI variants, and using either a weighted (unbroken line) or unweighted (dashed line) strategy. The histogram in the background displays the number of SNPs involved in construction of the allelic score at each corresponding SNP inclusion threshold for the “All variants” condition.

Figures 1 through 3 display the proportion of variance in each of the different intermediate variables (i.e. BMI, CRP, and LDLc respectively) within the ALSPAC cohort explained by a genome-wide allelic score of variants constructed according to different SNP inclusion thresholds. Figure 1 shows the results for BMI when all the observed genotypes were used in calculation of the scores and when regions around known variants were excluded from construction of the scores. In the case of the genome-wide scores including the known regions, the weighted score explained from 2.3% to 4.9% of the phenotypic variance in BMI depending on the SNP inclusion threshold, whereas the unweighted score explained from 2.1% to 3.9% of the variance. The weighted score explained more of the phenotypic variance in BMI than the unweighted score across all SNP inclusion thresholds tested. In the case of the weighted score, the proportion of variance explained tended to be greatest when the SNP inclusion threshold was liberal (i.e. the more SNPs included in construction of the score the better). In contrast, the predictive ability of the unweighted score reached a maximum at the p<0.2 selection threshold, but decreased either side of this maximum as the threshold became more or less conservative. Constructing an allelic score using only the known variants explained 3.2% of the variance in BMI when weighted and 2.3% of the variance in BMI when using an unweighted score. Interestingly using known variants explained smaller amounts of the phenotypic variance than that explained by the best weighted genome-wide predictors- even with the known regions removed.

**Figure 2:**
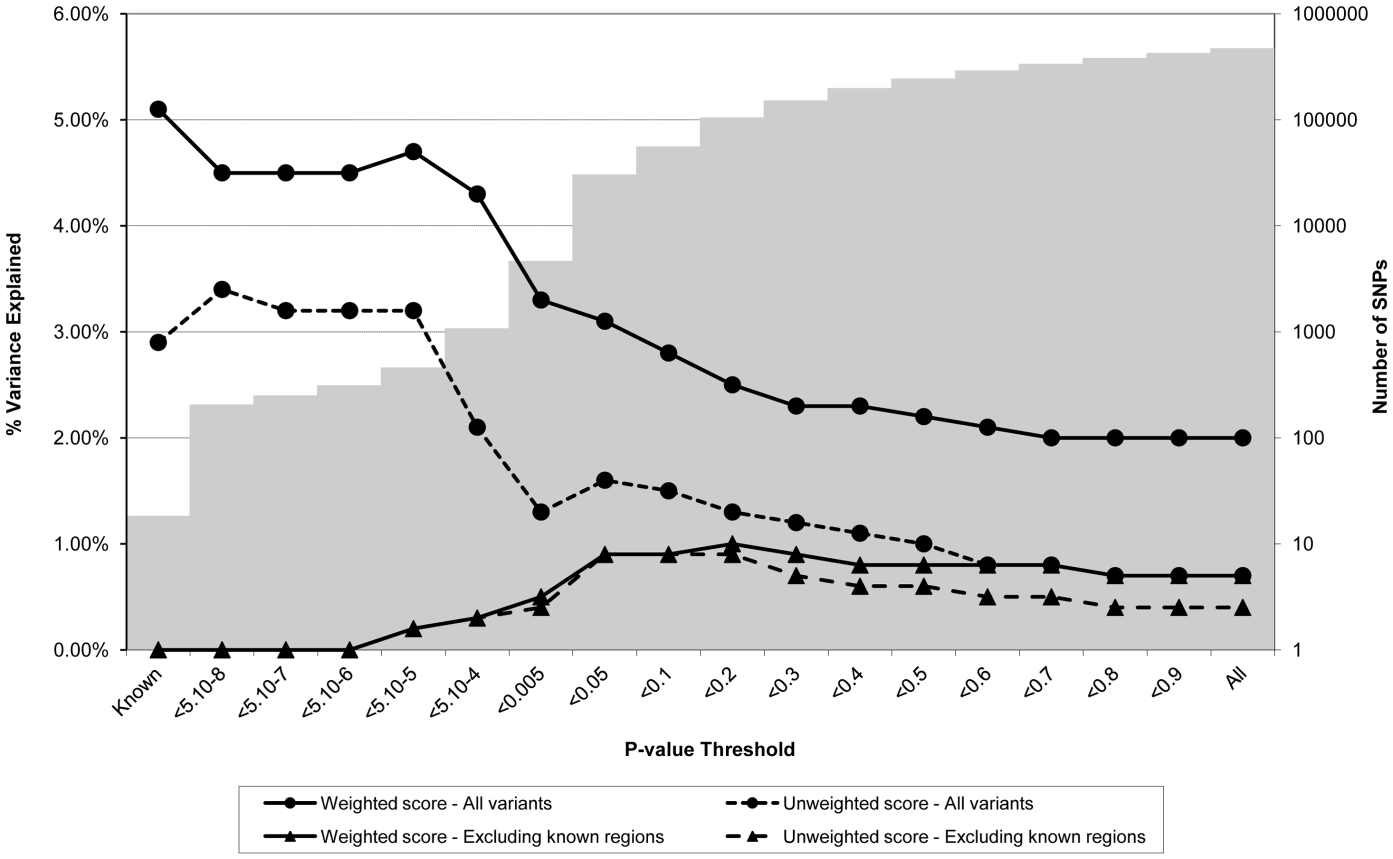
Association between polygene score and CRP measured at age nine in the ALSPAC cohort. Association between polygene score and CRP measured at age nine using different p-value thresholds for the construction of the score in ALSPAC children (N = 4251). The lines joining the circles display the results for allelic scores calculated by using genotyped variants from across the genome in either a weighted (unbroken line) or an unweighted (dashed line) fashion. The lines joining the triangles display scores calculated similarly but excluding all variants +/−1 MB around 18 known CRP variants, and using either a weighted (unbroken line) or unweighted (dashed line) strategy. The histogram in the background displays the number of SNPs involved in construction of the allelic score at each corresponding SNP inclusion threshold for the “All variants” condition.

**Figure 3:**
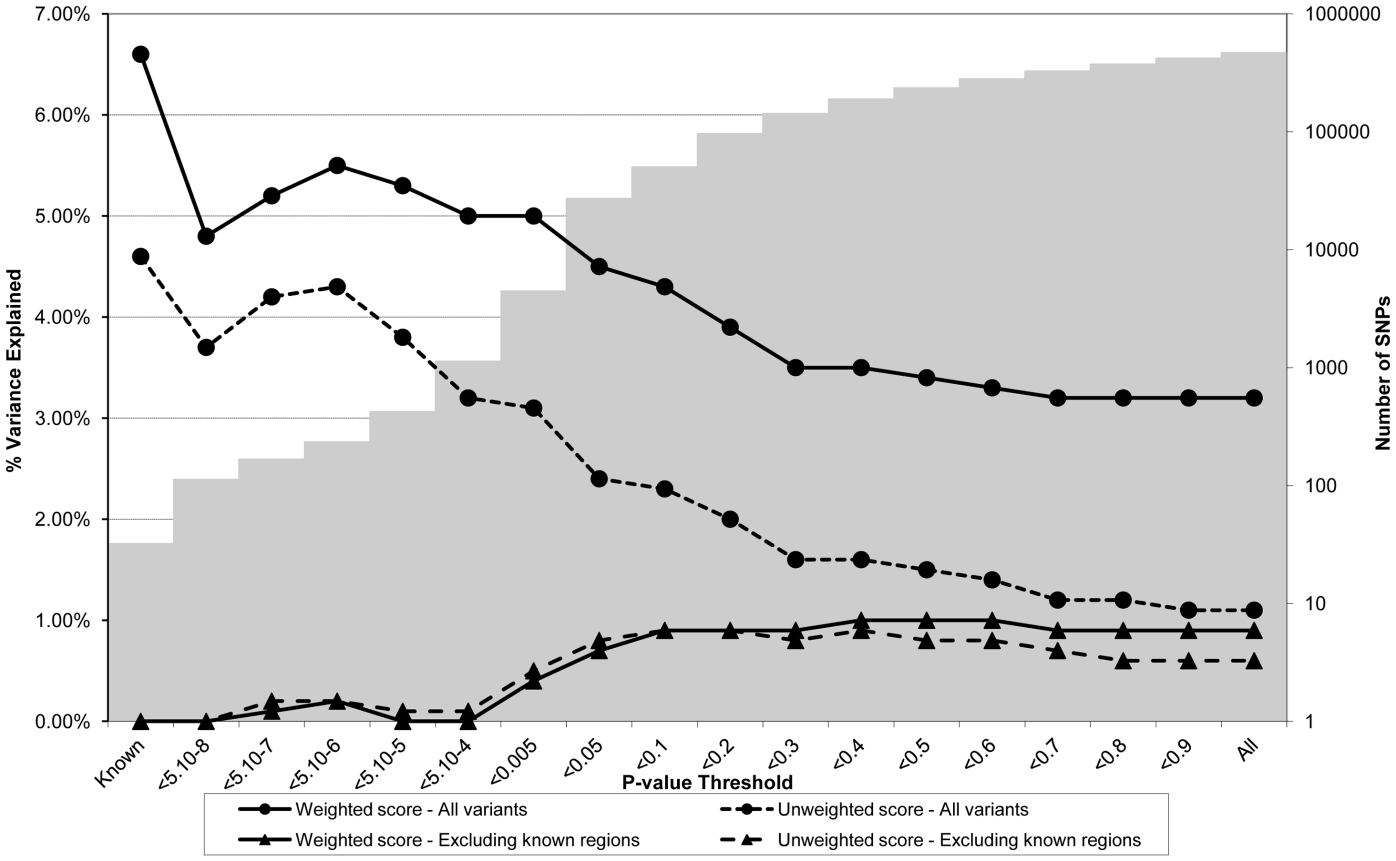
Association between polygene score and LDLc measured at age nine in the ALSPAC cohort. Association between polygene score and LDLc measured at age nine using different p-value thresholds for the construction of the score in ALSPAC children (N = 4251). The lines joining the circles display the results for allelic scores calculated by using genotyped variants from across the genome in either a weighted (unbroken line) or an unweighted (dashed line) fashion. The lines joining the triangles display scores calculated similarly but excluding all variants +/−1 MB around 37 known LDLc variants, and using either a weighted (unbroken line) or unweighted (dashed line) strategy. The histogram in the background displays the number of SNPs involved in construction of the allelic score at each corresponding SNP inclusion threshold for the “All variants” condition.

Figure 2 shows the results for CRP levels, which appear quite different to the results for BMI. In the case of the genome-wide scores including the known variants, the weighted score explained from 2.0% to 4.7% of the phenotypic variance in CRP depending on the SNP inclusion threshold, whereas the unweighted score explained from 0.7% to 3.4% of the variance. The weighted score explained more of the phenotypic variance in CRP than the unweighted score across all SNP inclusion thresholds tested. For both the weighted and unweighted scores, the proportion of variance explained was greatest when the SNP inclusion threshold was conservative (i.e. only SNPs with strong evidence of association included in construction of the score). Similarly, the greatest variance in CRP levels was explained using a weighted allelic score derived from the known variants only. When the known regions were removed from the construction of the scores, the greatest variance was explained using a SNP inclusion cut-off of around p<0.2, whilst the addition of SNPs with higher p values decreased the scores' explanatory ability slightly.

Figure 3 shows the results for LDLc levels. The pattern of results appeared similar to that for CRP in that the proportion of variance explained was greatest when the SNP inclusion threshold was conservative. In the case of the genome-wide scores including the known variants, the weighted score explained from 3.2% to 5.5% of the phenotypic variance in LDLc depending on the SNP inclusion threshold, whereas the unweighted score explained from 1.1% to 4.2% of the variance. The weighted score explained more of the phenotypic variance in LDLc than the unweighted score across all SNP inclusion thresholds tested. Similarly, the most variance in LDLc levels was explained using an allelic score derived from known variants only. When the known regions were removed from the construction of the scores, the most variance explained was obtained using cut-offs in the range 0.4<p<0.6, although the inclusion of extra genotype information decreased the scores' explanatory ability slightly.

We also examined the effect of pruning our data for linkage disequilibrium (LD) before constructing the allelic scores (Figures S1 through S3). All three variables showed similar patterns of results, namely thinning the SNP data improved the amount of variance explained in the biological intermediate when the SNP inclusion threshold was conservative (low p value), but decreased the predictive ability of genome-wide scores at liberal SNP thresholds. The corollary was that the best prediction for CRP and LDLc was produced at conservative SNP inclusion thresholds, whereas the best prediction occurred for BMI at high thresholds. In particular, the LDLc and CRP score thinned for LD showed marked improvement over an allelic score that had not been thinned for LD at conservative SNP inclusion thresholds.

### Performance of allelic scores in the QIMR twins replication cohort

We attempted to “replicate” the pattern of associations observed in the ALSPAC cohort by performing similar analyses in a sample of Australian twins (QIMR twins) who did not participate in the original meta-analyses of CRP, BMI and LDL. The results of these replication analyses are presented in Figures S4 through S6. In general, the proportion of variance explained by the allelic scores for these traits was lower than that explained in ALSPAC, but the pattern of results were roughly similar (i.e. weighted scores performed better than unweighted scores; allelic scores consisting of known variants performed better than genome-wide scores for LDLc and CRP, whereas genome-wide scores explained more phenotypic variance than scores consisting of known variants for BMI etc.; complement scores with the known variants removed could still explain significant amounts of the phenotypic variance etc).

### Performance of allelic scores in the WTCCC

Table 1 and Tables S1 through S3 display the results of the test of association between case-control status in the WTCCC and weighted genome-wide allelic scores calculated from all SNPs across the genome (i.e. the columns in Table 1 labelled “GW Score”), a weighted allelic score constructed from variants in known regions which met p<5×10^−8^ in the relevant GWAS meta-analysis (i.e. the columns in Table 1 labelled “Known”), and a weighted genome-wide score with SNPs from known regions removed from its construction (i.e. the columns in Table 1 labelled “Complement”). In the case of BMI, an allelic score consisting of known variants only showed strong evidence of being related to type 2 diabetes in the expected direction. As the threshold for SNP inclusion became more relaxed, the BMI score also showed nominal evidence of association with other diseases most notably bipolar disorder. These genome-wide scores were also very strongly related to risk of type 2 diabetes, more so than the score constructed from the known regions only. This is expected if the relationship between BMI and type 2 diabetes is causal, since the known variants explained less variation in the BMI intermediate than the genome-wide allelic scores. The fact that the genome-wide score with the known regions removed also showed strong association with type 2 diabetes shows that these associations do not solely reflect the effect of variants within *FTO* and other BMI genes known to be reliably associated with type 2 diabetes.

**Table 1:** Association between case-control status in the WTCCC and either a weighted genome-wide score consisting of all SNPs across the genome (“GW Score”), a weighted allelic score consisting of highly significant SNPs (p<5×10^−8^) from known regions only (“Known”), or a weighted genome-wide score consisting of all SNPs across the genome with SNPs from known regions removed from its construction (“Complement”).

	BMI						CRP						LDLc					
	GW Score		Known		Complement		GW Score		Known		Complement		GW Score		Known		Complement	
	Dir	P	Dir	P	Dir	P	Dir	P value	Dir	P value	Dir	P	Dir	P value	Dir	P value	Dir	P
BD	−	0.051	−	0.62	−	0.026	+	0.37	+	0.11	+	0.96	−	0.049	−	0.88	−	0.059
CHD	+	0.37	+	0.17	+	0.57	+	0.028	+	0.80	+	0.079	+	1.7×10^−3^	+	9.2×10^−3^	+	0.049
HT	−	0.76	−	0.58	+	0.76	+	0.20	+	0.23	+	0.53	−	0.011	−	0.75	−	0.012
CD	−	0.97	+	0.90	+	0.99	+	2.9×10^−4^	+	0.051	+	0.011	−	0.73	−	0.76	−	0.71
RA	−	0.18	+	0.15	−	0.085	+	0.17	+	0.028	+	0.69	−	0.26	−	0.25	−	0.50
T1D	−	0.97	+	0.77	+	0.85	+	0.020	+	0.15	+	0.033	−	0.018	+	0.58	−	0.20
T2D	+	<2×10^−16^	+	4.3×10^−7^	+	1.8×10^−12^	+	7.6×10^−8^	+	0.50	+	2.1×10^−7^	+	0.66	−	0.12	+	0.48

See Tables S1 through S3 for a complete list of results.

BD = Bipolar Disorder; CHD = Coronary Heart Disease; HT = Hypertension; CD = Crohn's Disease; RA = Rheumatoid Arthritis; T1D = Type 1 Diabetes; T2D = Type 2 Diabetes.

Dir = Direction of effect; P = P value.

As expected, the allelic scores indexing CRP derived from the known regions did not show strong evidence of association with coronary heart disease or type 2 diabetes, but did show nominal evidence of association with the auto-immune disease rheumatoid arthritis (Table 1). In sharp contrast, the genome-wide allelic scores indexing CRP showed strong evidence of association with some diseases (Table 1 and Table S2) especially types 1 and 2 diabetes, Crohn's disease, rheumatoid arthritis and coronary heart disease- depending on the threshold chosen for score construction (note that almost all allelic scores were associated with increased risk of disease). It is important to note that in most cases the strength of evidence for association with affection status tended to increase as the inclusion threshold became more liberal, yet the proportion of variance explained in the biological intermediate is likely to have decreased (Figure 2). Likewise, for some thresholds, the unweighted score provided stronger evidence of association with disease than the weighted score, even though the weighted score is likely to have explained more variance in the CRP intermediate (Figure 2 and Table S2).

The allelic scores indexing LDLc constructed from the known regions only, were associated with coronary heart disease (in the expected direction), but were not associated with any of the other diseases. In contrast, the genome-wide allelic scores showed unexpected nominal associations with hypertension (decreased risk), type I diabetes (reduced risk), and bipolar disorder (decreased risk) at some of the inclusion thresholds (Table S3). Similar to the case with CRP, as the SNP inclusion threshold became more liberal, the number of likely spurious associations increased, whilst the proportion of variance explained in LDLc is likely to have decreased (Figure 3).

We also examined the effect of pruning our data for LD before constructing the allelic scores (Tables S4 through S6). Results were similar to that obtained using un-pruned scores in that scores constructed from known variants tended to strongly predict one disease only (e.g. BMI score and type II diabetes, LDLc score and coronary heart disease), whereas genome-wide scores were associated with many different conditions. Interestingly a thinned weighted score of robustly associated LDLc variants predicted CHD very strongly (p = 2.3×10^−8^) consistent with the enhanced ability of this score to predict intermediate LDLc levels.

The results of our power calculations are displayed in Table 2. As expected, power to detect association increased as the proportion of variance explained in the biological intermediate increased, the causal effect of the intermediate on the disease became stronger, and as the prevalence of disease decreased. In the case of allelic scores for BMI/CRP/LDLc comprised entirely of known variants (which explain in the vicinity of 5% of the phenotypic variance in the intermediate), we expect 2000 cases and 3000 controls to provide good power to detect moderate to strong causal effects of the biological intermediate on disease. In contrast, using single variants which might typically explain 0.1% of the phenotypic variance in the biological intermediate would offer poor power to detect association. We expect 50000 cases and 50000 controls to provide high power to detect association in the case of allelic scores which explain >5% of the phenotypic variance in the biological intermediate. Similarly, allelic scores which explain 1% of the variance in the intermediate should still be sufficient to detect moderate or strong causal links between the intermediate and disease in this scenario. Again testing individual SNP variants which explain small proportions of the variance would provide very little power to detect association.

**Table 2:** Approximate power to detect association between an allelic score indexing a biological exposure and disease.

				2000 Cases 3000 Controls	50000 Cases 50000 Controls
σ_G_^2^	β	σ_L_^2^	Disease Prevalence	Power	Power
10%	.1	0.1%	1%	83.8%	100%
10%	.2	0.4%	1%	100%	100%
10%	.5	2.5%	1%	100%	100%
10%	.1	0.1%	5%	66.2%	100%
10%	.2	0.4%	5%	99.7%	100%
10%	.5	2.5%	5%	100%	100%
10%	.1	0.1%	10%	57.0%	100%
10%	.2	0.4%	10%	99.0%	100%
10%	.5	2.5%	10%	100%	100%
10%	.1	0.1%	20%	48.3%	100%
10%	.2	0.4%	20%	97.0%	100%
10%	.5	2.5%	20%	100%	100%
5%	.1	0.05%	1%	55.0%	100%
5%	.2	0.2%	1%	98.6%	100%
5%	.5	1.25%	1%	100%	100%
5%	.1	0.05%	5%	39.1%	99.1%
5%	.2	0.2%	5%	92.0%	100%
5%	.5	1.25%	5%	100%	100%
5%	.1	0.05%	10%	32.7%	94.3%
5%	.2	0.2%	10%	85.6%	100%
5%	.5	1.25%	10%	100%	100%
5%	.1	0.05%	20%	27.3%	81.0%
5%	.2	0.2%	20%	77.4%	100%
5%	.5	1.25%	20%	100%	100%
1%	.1	0.01%	1%	15.4%	14.6%
1%	.2	0.04%	1%	46.2%	99.9%
1%	.5	0.25%	1%	99.7%	100%
1%	.1	0.01%	5%	11.7%	3.0%
1%	.2	0.04%	5%	32.5%	94.0%
1%	.5	0.25%	5%	96.4%	100%
1%	.1	0.01%	10%	10.4%	1.3%
1%	.2	0.04%	10%	27.2%	80.4%
1%	.5	0.25%	10%	92.2%	100%
1%	.1	0.01%	20%	9.3%	0.5%
1%	.2	0.04%	20%	22.8%	58.8%
1%	.5	0.25%	20%	85.8%	100%
0.1%	.1	0.001%	1%	6.0%	0%
0.1%	.2	0.004%	1%	9.1%	0.4%
0.1%	.5	0.025%	1%	31.4%	92.2%
0.1%	.1	0.001%	5%	5.7%	0%
0.1%	.2	0.004%	5%	7.6%	0.1%
0.1%	.5	0.025%	5%	22.1%	54.7%
0.1%	.1	0.001%	10%	5.5%	0%
0.1%	.2	0.004%	10%	7.1%	0%
0.1%	.5	0.025%	10%	18.7%	33.1%
0.1%	.1	0.001%	20%	5.4%	0%
0.1%	.2	0.004%	20%	6.7%	0%
0.1%	.5	0.025%	20%	16%	17.5%

The model is parameterized in terms of the percentage of variance in the biological intermediate explained by the SNP (σ_G_^2^), the strength of the causal relationship between the biological intermediate (β) and liability of disease, which together determine the amount of variance in disease liability explained by the SNP (σ_L_^2^), and the prevalence of disease. Estimates of power are presented for 2000 cases and 3000 controls (α = 0.05), and for 50000 cases and controls (α = 1.1×10^−7^).

## Discussion

In this paper we investigated whether it might be possible to correlate allelic scores which reference biological intermediates with disease status in case control studies, and in so doing provide proof of principle for a method which could be used to screen for thousands of possible associations between intermediate variables and disease. We began by demonstrating that allelic scores explained non-trivial proportions of the phenotypic variance in BMI, CRP, and LDLc, even when known loci were taken into account and removed from the construction of those scores. This result confirms the existence of many common variants of small effect scattered across the genome that were tagged by SNPs on the genome-wide platform, but did not reach genome-wide levels of significance in the meta-analysis. Our results are also consistent with studies using analogous methodologies in other complex traits and diseases, and were a key motivating force in our development of this approach [9], [11], [22].

The proportion of variance explained in CRP by a weighted score of known variants in ALSPAC (5.1%) was similar to that reported in the CRP meta-analysis by Dehghan et al. (2011) who also estimate that a weighted score of confirmed variants explained around 5% of the phenotypic variance in CRP [14]. Teslovich et al. (2010) report that a weighted score of genome-wide significant LDLc associated variants explained 12.2% of the phenotypic variance in LDLc [3]. The lower figure in ALSPAC (6.6%) is probably due to a combination of factors including the ALSPAC analysis not being performed on fasting bloods, the ALSPAC analysis not including secondary loci in the calculation of variance explained by known variants, and the possibility that true differences exist in the size and identity of genetic variants that affect LDLc levels in adults and children. Speliotes et al. (2010) reported that a weighted score of all known BMI associated loci explained 1.45% of the variance in BMI [13]. The proportion of variance explained in the ALSPAC cohort was higher, at 3.2% for the known variants. It is unclear why the proportion of variance explained was greater in ALSPAC but may have to do with the fact that participants were all young children of the same age (9 years) and so there was little variation due to differences in age, sex, puberty, growth in later life etc. In terms of the proportion of phenotypic variance explained in the intermediate variable, a weighted allelic score generally yielded superior performance to an unweighted score. This was expected since weighting includes prior information in that SNPs with large estimated effect sizes contribute more to the overall score. We also note that in the case of the BMI, and CRP analyses, the difference in variance explained between using weighted and unweighted scores became less apparent when the known SNPs (i.e. SNPs with the largest effect sizes) were excluded from the calculations. This was also expected since smaller effects are less likely to be estimated precisely and hence it is more difficult to weight these SNPs appropriately.

The SNP selection threshold producing the allelic score that explained the most variance in the intermediate differed across the variables. This is not surprising since different phenotypes have different underlying genetic architectures and differ in the extent to which the variants that influence them are tagged by SNPs on genome-wide chips. In general, the best allelic scores for BMI were produced using liberal cut-offs. In contrast, allelic scores for CRP and LDLc tended to perform best when constructed from conservative threshold cut-offs. A possible reason for the discrepancy is that CRP and LDLc are both influenced by loci of major effect in ALSPAC (i.e. the variants rs4420638 (R^2^ = 2.5%, p = 1.7×10^−25^) and rs10401969 (R^2^ = 2.0%, p = 1.1×10^−20^) explain disproportionately large proportions of the variance in LDLc, whilst the variants. rs2794520 (R^2^ = 2.4%, p = 5.2×10^−24^) and rs4420065 (R^2^ = 2.3%, p = 4.5×10^−23^) explain large portions of the variance in CRP- see also the last two columns in Table S7). At stringent p value cut-offs therefore, these scores primarily reflect genuine quantitative trait loci of moderate effect which explain decent proportions of the variance in these phenotypes. In contrast, as the cut-offs become more liberal, it is likely that the scores become contaminated by unassociated SNPs and markers of small effect that have less precisely estimated contributions. As a result, the amount of variance explained in the phenotype is reduced. We note that the same pattern of association with a few loci contributing disproportionately large effects for these phenotypes was also seen in the QIMR twins replication cohort although to a less pronounced degree (Table S8). A similar observation has been noted in studies that have used genome-wide allelic scores to predict disease status in auto-immune diseases that involve genetic loci of large effect in the major histocompatibility region [9]. In contrast, in the case of BMI, no single variant contributes disproportionately to explaining trait variance (Table S7), and so the explanatory power of the allelic scores is facilitated through the addition of many variants of small effect scattered across the genome [9], [11], [22].

Another contributing factor to the discrepancy between the BMI and CRP/LDLc scores is that much more phenotypic variance is explained by the residual polygenic score in the case of BMI (∼3%) than LDLc or CRP (∼1%) (see the lower two lines in Figures 1 through 3 which show the variance explained by the polygenic scores when the effect of known loci are removed from construction of the scores). It is unclear why this is the case, but could be due to many factors including genuine differences in the genetic architecture of the traits, a difference in the extent to which loci that affect these traits are shared between adults and children (i.e. the original GWA meta-analyses typically involve adults whereas ALSPAC is a paediatric cohort, although the same phenomenon was also found in the QIMR twins, all of whom are adults), and differences in meta-analysis size (and hence power to detect genuine effects) from which the scores were constructed (e.g. the CRP meta-analysis was smaller than the other two studies). We note that the same pattern of association is also seen in the QIMR twins replication sample suggesting that the pattern of results is not cohort specific.

Pruning for LD enhanced prediction of the biological intermediates at conservative but not liberal SNP construction thresholds. This was most apparent for the CRP and LDLc associated allelic scores. We argue that at conservative thresholds, the contribution of individual variants to the biological intermediate is estimated most precisely when data has been thinned for LD (i.e. the signal is at its most “pure”). In addition, such a score will also capture secondary signals at known loci, which may help explain why the thinned scores performed better than allelic scores consisting of known variants only (additional secondary loci were not included in the known variant scores in this study). However, in the case of genome-wide allelic scores constructed from liberal thresholds, the signal from loci of small effect scattered across the genome are not estimated as precisely as signals from known variants, and so, pruning for LD has the effect of removing the signal from these scores.

Importantly we have demonstrated that genome-wide allelic scores can still explain meaningful portions of the phenotypic variance, even in situations where known variants have been excluded from the calculation of the allelic score. For example, in the case of BMI, a genome-wide allelic score still explained ∼3.4% of the variance in BMI even after known regions were removed from construction of the scores (a high figure was also noted in the QIMR twins replication set). The exciting implication is that in the case of other biological intermediate variables of interest for which there are currently no known genetic variants, genome-wide allelic scores may still be able to proxy these variables and could subsequently be used in tests of association with diseases of interest.

We note that the present study has benefitted from very large genome-wide association meta-analyses from which the SNPs that comprise the genome-wide allelic scores were selected [3], [13], [14]. A recent study by Demirkan et al. (2012) found that a polygenic score only explained 2.6% of the phenotypic variance in LDLc [23]. Demirkan et al suggested that this low figure might have been a consequence of the small size of the discovery sample on which their weighting scheme was based (i.e. ∼20,000 individuals as compared to the ∼100,000 individuals used in Teslovich et al which forms the basis of this study) and consequently decreased precision in estimating effect sizes and direction of effects. These factors (plus the existence of some loci of large effect in ALSPAC) may also partially explain the comparatively better predictive ability in our study (particularly at more liberal p value inclusion thresholds) as construction of allelic scores in ALSPAC was based on the much larger Teslovich et al meta-analysis. Although the proportion of variance explained in ALSPAC was greater than that found by Demirkan et al., we note that the pattern of variance explained across the different SNP inclusion thresholds was similar across both studies as well as the QIMR replication set (i.e. as the threshold becomes less stringent, less variance is explained).

In addition, all of the intermediate variables that we examined exhibit substantial heritability. Our method relies on these preconditions and it remains to be seen how useful the approach will be in scenarios where the genome-wide association meta-analysis is small and/or the variables of interest have low heritabilities. The corollary is that although our method may have worked adequately in the case of these three variables, it does not necessarily follow that our success will translate to other phenotypes and we suggest that those wishing to apply the approach proceed with caution in its application and interpretation.

Finally, we note that whilst we have combined a simple threshold based SNP selection procedure with a straightforward weighting, considerable potential exists to make the approach more powerful by tailoring the selection of SNPs and combining them in more optimal ways. These approaches could include machine learning or lasso regression for example [24].

### Associations between allelic scores and WTCCC disease status

Our method successfully identified established causal relationships between BMI and type 2 diabetes, and LDLc and coronary heart disease. This is consistent with the power calculations presented in Table 2 which suggested that 2000 cases, 3000 controls and an allelic score explaining roughly 5% of the variance in a biological intermediate provided good power to detect moderate to strong relationships between the intermediate and the disease outcome. Interestingly, in the case of BMI, the genome-wide allele score was more strongly related to type 2 diabetes than the allelic score constructed from the known variants only. This observation is consistent with our demonstration that the genome-wide allelic score explained greater proportions of the variance in BMI than the allelic score comprised from the known variants only. In fact, even the genome-wide allelic score indexing BMI with the regions around the known BMI SNPs removed also correlated strongly with type 2 diabetes. Taken together these results suggest that the BMI-type 2 diabetes association does not solely reflect the effect of variants within *FTO* and other BMI genes known to be reliably associated with type 2 diabetes, and also further strengthens the proposition that genome-wide allelic scores may have promise in indexing intermediates, even in situations where there are no known variants underlying the intermediate (i.e. as we have artificially done here by removing the variants from the known BMI associated regions).

It is noteworthy that our method did not appear to detect other observational associations thought to reflect causal relationships. For example, there have been associations reported between BMI and coronary heart disease [25], and BMI and hypertension [26]. Table 2 implies that the most probable explanation for this failure is statistical power in that 2000 cases and 3000 controls is unlikely to provide sufficient power to detect weaker causal relationships between biological intermediates and disease.

Another notable finding involves the relationship between allelic scores that index CRP levels and disease. It is interesting that the genome-wide allelic score that indexed CRP correlated with many of the WTCCC diseases, whereas the allelic score constructed from the known regions only, did not. Mendelian Randomization studies have shown that CRP is unlikely to cause several diseases to which it had been linked including type II diabetes and coronary heart disease, but rather the observational associations are probably a secondary consequence of the disease itself or due to latent confounding [6]–[8]. Given that the genome-wide allelic score actually explained less variance in CRP level than the known variant score (i.e. 2% versus 5%), we suggest that a causal effect of CRP on the different diseases is unlikely, but rather that genetic pleiotropy and the lack of specificity of the genome-wide allelic score is the most likely explanation for this difference. For example, many BMI associated SNPs are present at quite low levels of significance in the CRP GWAS meta-analysis (Table S9), although in this case, not at genome-wide significant levels. Furthermore bidirectional Mendelian Randomization studies have demonstrated that higher BMI leads to elevated CRP, not vice versa [27]. Scores created from these SNPs would therefore show association with CRP level, BMI and consequently (through BMI) greater risk of type 2 diabetes. A similar explanation probably underlies the apparent association between the CRP genome-wide allele score and the auto-immune diseases except the mediating variable is likely to be some immune parameter that affects both CRP and risk of rheumatoid arthritis/type I diabetes/Crohn's disease.

Similar results were also seen for the allelic scores which indexed LDLc. Whilst the allelic score consisting of known variants only correlated with CHD as expected, the genome-wide allelic score showed unexpected nominal correlations with other diseases including hypertension and type I diabetes. As the genome-wide score explained less variance in the intermediate than the allelic score derived from the known variants, we believe that this association also reflects unwanted genetic pleiotropy and lack of specificity in the genome-wide score for similar reasons alluded to above.

These results highlight the potential advantages and disadvantages of using genome-wide allelic scores to index biological intermediates. As the number of SNPs that comprise the allelic score increases, the score may gain power in terms of explaining variance in the exposure/mediator of interest (e.g. as in the case of BMI and possibly also in the case of many other biological intermediates for which no known variants exist), but the downside is that the score potentially loses specificity and may produce associations with disease that do not necessarily reflect causal relationships. Our results also indicate that whilst thinning genome-wide SNP data for LD might be useful in terms of explaining more variance in the biological intermediate (and hence power to detect a true causal relationship between intermediate and disease) it is unlikely to mitigate the endemic issues of pleiotropy and lack of specificity of the genome-wide scores (i.e. likely spurious associations were observed with thinned data also).

A logical strategy therefore might be to use confirmed variants only to generate allelic scores in those situations where there are individual SNPs known to explain variance in the intermediate of interest. In the absence of genetic pleiotropy, these allelic scores should be powerful and specific to the biological intermediate of interest. In contrast, in those situations where there are no variants that are known to affect the intermediate, genome-wide allelic scores could be employed to investigate a possible relationship with disease. In this way a balance can be struck between maintaining power and attempting to preserve specificity, although we note that, even in the case of an allelic score constructed completely from known variants, there is no guarantee that such a score will be completely specific for the intermediate of interest and that there will not exist other paths from SNP to disease. Thus, in the presence of an association between an outcome of interest and an allelic score of known variants that index an exposure, we strongly suggest follow up using formal Mendelian Randomization methodologies [5].

In the situation where there are no variants known to underlie the biological intermediate of interest, formal Mendelian Randomization will not be possible, and so it will be difficult to determine whether an association between a genome-wide allelic score and a disease of interest reflects a causal relationship. In addition, our results suggest that lack of specificity and contamination of genome-wide scores through genetic pleiotropy will mean that many of these associations will be “spurious” and will not reflect causal effects of the intermediate on the outcome. However, it might still be possible to get some indication of whether the data are consistent with a causal effect of the intermediate on the disease by examining the pattern of association across different SNP construction thresholds and weighting schemes. For example, in the presence of a causal influence of the biological intermediate on disease risk, we would expect that the strongest evidence for a relationship between the allelic score and affection status occurs at those conditions/thresholds that simultaneously explain the greatest proportion of phenotypic variance in the intermediate. If this pattern occurs in the data, then the results are at least consistent with a causal effect of the intermediate on disease risk (although this of course does not prove a causal relationship). If this pattern of results is not present in the data, then it suggests that the association is more likely due to genetic pleiotropy and/or lack of specificity in the genome-wide score. For example, in the present set of results, the strongest evidence for associations between LDLc and coronary heart disease occurred at those conditions where a thinned weighted allelic score concurrently explained the greatest variance in the intermediate phenotype, consistent with a causal relationship between LDLc and coronary heart disease (Table S6). In contrast, the strongest evidence for a relationship between CRP and type II diabetes occurred at those thresholds where the variance explained in CRP was at a minimum, suggesting a spurious relationship between the two variables.

Whilst our approach has several similarities to Mendelian Randomization [5], we stress that our method is designed as a screening tool that provides preliminary evidence for a possible causal relationship between an intermediate which may be worth following up in focused future studies. The method is not intended as a means of providing conclusive evidence for a causal relationship between two variables. Specifically, our approach does not rule out the possibility of a pleiotropic relationship between the SNPs that index the intermediate and the disease, nor does it rule out the possibility that an allelic score (particularly a genome-wide allelic score) has been “contaminated” by SNPs as a result of reverse causation. For example, if type 2 diabetes were to cause an elevation of CRP levels, then it is conceivable that some type 2 diabetes SNPs might show association in a GWAS meta-analysis of CRP. Therefore, allelic scores indexing CRP which are based on this GWAS meta-analysis will also show association with type 2 diabetes even though the direction of causation may be from the disease to the biological intermediate. The point we do stress is that our method is useful for flagging putative causal relationships across potentially thousands of biological intermediates, and we recommend following up interesting associations by e.g. formal Mendelian Randomization analysis, randomized controlled trials, mechanistic studies etc.

### Possible biases

In this study we were careful to ensure that the cohorts used to assess the amount of phenotypic variance explained in the biological intermediates (i.e. ALSPAC and QIMR twins) were not also present in the original discovery meta-analyses of CRP, BMI and LDLc (NB. the QIMR twin individuals who contributed to the Teslovich et al. meta-analysis were from different families to those used in the present study) [3]. Inclusion of the same individuals who were in the discovery meta-analysis would have inflated the proportion of variance explained in the biological intermediate particularly when liberal significance thresholds were used in construction of the genome-wide allelic scores [28]. Likewise, we were also careful to exclude the 1958 birth cohort as a control group when examining the predictive ability of allelic scores to determine case control status in the WTCCC (the 1958 birth cohort contributed to the discovery meta-analyses of BMI, LDLc and CRP). We were also able to exclude the Wellcome Trust hypertension, coronary heart disease, type 2 diabetes and control group results from the original Speliotes et al. BMI meta-analysis so that the inclusion of these groups did not bias our analyses of BMI SNPs and WT case control status. However, we were unable to remove the Wellcome Trust hypertension cohort from the Teslovich et al. meta-analysis [3] so this fact should be borne in mind when interpreting the results of these analyses (although LDLc score failed to significantly correlate with hypertension status across most thresholds).

### Extending the allelic score approach to data mine thousands of molecular phenotypes

The most exciting implication of our work, is that the approach could be successfully extended to examine hundreds of thousands of molecular phenotypes. GWAS of molecular technologies that target the transcriptome [29], metabolome [30], and most recently, the methylome [31] have begun to appear in the literature with increasing frequency. Allelic scores which index levels of transcription, methylation and levels of metabolites etc could be constructed and subsequently used as instruments to screen for possible associations with hundreds of traits and diseases, in tens of thousands of individuals. Our power calculations demonstrate that such an approach is realistic in the very large GWA met-analyses that currently exist.

### Conclusions

When genetic variants that affect a biological intermediate are known a priori, we recommend using these SNPs exclusively to construct allelic scores that proxy for the biological intermediate of interest. If a subset of the known variants is specific for the intermediate, then we recommend using these variants solely in construction of the allelic score and excluding variants with pleiotropic effects that may complicate interpretation of the effect. This minimizes (although certainly does not abolish) concerns due to genetic pleiotropy and lack of specificity. We stress that a positive association between an allelic score of known variants and disease does not prove a causal relationship between the intermediate and disease but merely flags an interesting association that may be worthy of follow up by more formal methods (e.g. proper Mendelian Randomization analysis etc).

In the situation where the identity of individual genetic variants affecting the biological intermediate are unknown, a genome-wide allelic score can be used to proxy the trait of interest. In this situation we recommend employing the strictest p-value inclusion threshold in construction of the genome-wide allelic score that maximizes the amount of variance explained in the biological intermediate. In this way, the amount of variance explained in the intermediate is maximized, whilst simultaneously attempting to minimize the number of SNPs with pleiotropic effects that go into construction of the score. Since the potential for spurious association due to pleiotropy is particularly high when using hundreds or thousands of SNPs, we recommend that if genome-wide scores are used, that their results are cautiously interpreted and followed up with care.

In conclusion, whilst genome-wide association studies have identified thousands of genetic variants underlying complex traits and diseases, a criticism of the approach has been that in many cases, knowledge of the risk variants underlying disease has yet to be translated into interventions or information that directly impacts clinical medicine and public health. Our idea is to use allelic scores that proxy biological intermediates to data mine genome-wide association studies. We would argue that our simple approach is an easy to understand statistical method which has the potential to identify possible causal relationships between these variables and disease outcomes, and through this, translate the findings from genetic research into information that is relevant to public health as in the case of Mendelian Randomization studies [32]. Our results suggest that our approach may even be possible in the case of biological intermediates where confirmed genetic variants are unknown *a priori* through the application of genome-wide allelic scores. Our method has the potential to revolutionize the way exposure-disease associations are identified in observational epidemiological studies and ensure that the considerable investment in genome-wide association studies over the past decade is maximized in terms of public health impact.

## Materials and Methods

### Participants

ALSPAC Cohort: ALSPAC is a population-based birth cohort study consisting initially of over 13 000 women and their children recruited in the county of Avon, UK in the early 1990s [33], [34]. Both mothers and children have been extensively followed from the mothers' early pregnancy onwards using a combination of self-reported questionnaires, medical records and physical examinations. DNA has been extracted for 10121 of the children from this cohort. Ethical approval for the study was obtained from the ALSPAC Law and Ethics Committee (IRB# 00003312) and the Local Research Ethics Committees (Bristol and Weston, Southmead, and Frenchay Health Authorities). Written informed consent was obtained from all participants in the study. Parents provided written informed consent for their child. Children's standing height at age 9 years was measured using a Harpenden Stadiometer. Weight was quantified using a Tanita Body Fat Analyser at the same age. Body mass index (BMI) was calculated as weight in kilograms divided by height in metres squared. Non-fasting blood samples were taken using standard procedures when the children were age 9 years, with samples immediately spun and frozen at −80°C. The measurements were assayed in 2008 after a median of 7.5 years in storage with no previous freeze–thaw cycles during this period. Plasma lipids (total cholesterol, triglycerides, and HDL cholesterol) were performed by modification of the standard Lipid Research Clinics Protocol using enzymatic reagents for lipid determination. C-reactive protein was measured by automated particle-enhanced immunoturbidimetric assay (Roche UK, Welwyn Garden City, UK). All assay coefficients of variation were <5%. All variables were inverse normal transformed for males and females separately.

QIMR Twins Replication Cohort: Data for Australian subjects were obtained from adult participants in twin and family studies conducted by the Queensland Institute of Medical Research [15], [16], [35]. BMI was calculated from clinically measured height and weight supplemented with self-report for those participants where clinical measurements were not available. CRP and LDL-C (calculated from the Friedewald equation) measurements were performed using Roche methods on Hitachi 917 or Modular P analysers. The data used here represent 4781, 2767 and 2630 individuals who had genome-wide SNP information and information on BMI, CRP and LDLc respectively. A maximum of one individual per family was used in these analyses, yielding a set of unrelated individuals who had not contributed any data to the previous BMI, CRP or LDLc meta-analyses.

WTCCC: We employed previously published data from the WTCCC in order to test the association between allelic scores and disease status [17]. Briefly, the WTCCC is a GWAS involving individuals with one of seven diseases: bipolar disorder (1868 individuals), coronary heart disease (1926 individuals), Crohn's disease (1748 individuals), hypertension (1952 individuals), rheumatoid arthritis (1860 individuals), type I diabetes (1963 individuals) or type II diabetes (1924 individuals), as well as a common set of 1478 unselected controls from the 1958 British Birth Cohort and 1458 from the National Blood Service.

### Genotyping

ALSPAC: A total of 9912 ALSPAC children were genotyped using the Illumina HumanHap550 quad genome-wide SNP genotyping platform by the Wellcome Trust Sanger Institute, Cambridge, UK and the Laboratory Corporation of America, Burlington, NC, USA. Individuals were excluded from further analysis on the basis of having incorrect sex assignments; minimal or excessive heterozygosity (<0.320 and >0.345 for the Sanger data and <0.310 and >0.330 for the LabCorp data); disproportionate levels of individual missingness (>3%); evidence of cryptic relatedness (>10% IBD) and being of non-European ancestry (as detected by a multidimensional scaling analysis seeded with HapMap 2 individuals, EIGENSTRAT analysis revealed no additional obvious population stratification and genome-wide analyses with other phenotypes indicate a low lambda). The resulting data set consisted of 8365 individuals and 488311 autosomal SNPs. SNPs with a minor allele frequency of <1% and call rate of <95% were removed. Furthermore, only SNPs which passed an exact test of Hardy–Weinberg equilibrium (p>5×10^−7^) were considered for analysis. Of these 8365 individuals, 5819 had BMI data, and 4251 had CRP and LDLc levels measured. In order to investigate the effect of specific SNPs from GWAS meta-analyses of BMI, CRP, and LDLc we used autosomal genotypic data that had been imputed using Markov Chain Haplotyping software (MACH v.1.0.16) and phased haplotype data from CEU individuals (Hapmap release 22, Phase II NCBI B36, dbSNP 126).

QIMR Twins Replication Cohort: Genotyping within this cohort was performed in multiple waves using Illumina SNP chips (317K ; 370K duo; 370K quad; 610K; or 660K). Details of cleaning, data merging and imputation protocols have been described extensively in Medland et al. (2009) [35]. Briefly, each wave of genotyping was screened for call rate and quality, following this the data sets were merged and checked for calling consistency using a series of overlapping samples which were included in multiple genotyping waves. The merged genotype sets were then screened for call rate <95% and quality (GenCall>.7), minor allele frequency <1%, and Hardy–Weinberg equilibrium (p>1×10^−6^). In addition, as the QIMR cohort contains data from nuclear families (including parents, twins, siblings, spouses and offspring) we also screened the genotypes to confirm reported relationships, check for unknown relatedness and identify Mendelian errors taking the conservative approach of dropping a SNP for all family members if the erroneous genotype could not be identified.

WTCCC: Individuals were genotyped at the Wellcome Trust Sanger Institute, Cambridge, UK using the Affymetrix 500K SNP chip. Genotype data were subjected to rigorous quality control measures (SNPs with MAF <1%, missing rate >5% or Hardy Weinberg p<5×10^−8^ were excluded) in order to remove poor quality SNPs as well as putatively related individuals and those of non-European ancestry (for a full description of the cohorts see the original WTCCC article [17]).

### Construction and testing of allelic scores

We were interested in whether allelic scores constructed from hundreds of thousands of SNPs across the genome might produce powerful instruments that explained larger proportions of the phenotypic variance in biological intermediates than allelic scores derived from combinations of known variants. We used recent large scale GWAS meta-analyses of BMI [13], CRP [14], and LDLc [3] to select SNPs that went into the construction of the genome-wide allelic scores. In other words, SNPs that met a certain p-value threshold in the GWAS meta-analysis were then used to construct an allelic score in the ALSPAC dataset (NB. ALSPAC was not part of the BMI, CRP, or LDLc meta-analyses).

Genome-wide allelic scores were constructed from directly genotyped SNPs in the ALSPAC children's samples using the profile scoring routine in the PLINK software package [36]. The profile score for each individual was derived as a sum across SNPs of the number of putative increaser alleles (0,1 or 2) at each locus multiplied by a weight. In the case of missing genotype data for an individual, expected dosage based upon allele frequency of the increaser alleles at the locus was used instead of the number of increaser alleles. We investigated two methods of constructing allelic scores- an unweighted strategy where each copy of the increaser allele provided a score of one, and a strategy where the contribution of each SNP was weighted by its regression coefficient from the relevant genome-wide meta-analysis [9]. We refer to these strategies as the “unweighted” and “weighted” strategies respectively. Allelic scores were constructed for BMI, CRP, and LDLc separately. We also constructed allelic scores using 32 variants known to affect BMI [13], 18 variants known to affect CRP levels [14], and 37 variants known to affect LDLc levels [3] (Table S7). These analyses were based on best guess genotypes from imputation using Markov Chain Haplotyping software (MACH v.1.0.16) and phased haplotype data from CEU individuals (Hapmap release 22, Phase II NCBI B36, dbSNP 126) as described previously.

In order to determine the amount of variance explained in the biological intermediates using different strategies, we constructed allelic scores using seventeen different p value inclusion thresholds from the GWAS meta-analyses ranging from liberally including all SNPs, to including only those SNPs that met a stringent genome-wide significant criterion of p<5×10^−8^. In order to separate the effect of known genetic variation from residual polygenic variation scattered across the genome, we also constructed genome-wide allelic scores using the strategies above, but excluding SNPs within one megabase either side of known variants (i.e. 32 regions in the case of allelic scores indexing BMI; 18 regions in the case of allelic scores indexing CRP; and 37 regions in the case of allelic scores underlying LDLc- for a complete list of loci, please see Table S7) and refer to these analyses as the “Complement” conditions. Child's phenotype (i.e. BMI, CRP or LDLc) was then regressed on allelic score to determine the percentage of variance explained by each of the scores. Similar analyses were also performed in the QIMR twins cohort in order to test how robust the observed patterns of associations were except that all analyses involving “known” variants used genotypic dosages rather than best guess genotypes.

We also examined the effect that thinning the SNP data for LD had on the predictive ability of the scores. We employed the “LD based results clumping” routine from the PLINK software package [36] to generate the thinned data. Briefly, this routine orders the GWA meta-analysis association p values from strongest to weakest. SNPs are then selected in this order, with the proviso that a variant cannot be included, if it is in LD with a previously selected SNP. For the purposes of this analysis we defined LD as the variants being r^2^>0.2 and within 250 kb of each other.

The ability of the allelic scores to predict case control status was tested using data from the WTCCC. Several disease groups from the WTCCC were present in the original BMI discovery meta-analysis [13]. Because of the possibility of inducing bias into the results because of this, these groups were removed and the Speliotes et al. BMI meta-analysis repeated according to the same protocols as outlined in the original paper [13]. In addition, since the 1958 Birth Cohort controls were also included in the original meta-analyses of CRP and LDL, we removed these individuals from the WTCCC control set (i.e. only individuals from the National Blood Donors Study remained as controls). Case-control status for each disease was regressed on allelic score and the direction of effect and p value were recorded. We tested a weighted genome-wide score consisting of all variants across the genome (unweighted in the case of LDLc), an allelic score consisting of variants from known regions only (i.e. SNPs that met p<5×10^−8^ in the meta-analysis of the relevant phenotype), and a weighted genome-wide allelic score with known variants (+/−500 KB) removed from the score's construction. This was to contrast the performance of a completely agnostic strategy (i.e. utilizing all the SNPs) versus the strategy of only using known regions in construction of the scores. Finally, we examined the performance of LD pruning (as defined above) on the ability of weighted allelic scores to predict case-control status in the WTCCC dataset.

### Power calculations

In order to investigate the power of our approach, we assumed the standard liability threshold model in which a continuous normal distribution of liability underlies risk of disease. Under this model, individuals who are affected have a liability exceeding a certain threshold, the value of which being determined by the prevalence of the disease in the population. In the situation where a SNP (or an allelic score of SNPs) affects a biological intermediate which then in turn affects likelihood of disease, power to detect association between the SNP and disease is determined by (a) the proportion of variance in the biological intermediate explained by the allelic score (denoted by σ_G_^2^), (b) the strength of the causal relationship between the intermediate and the disease (denoted by β) which together with σ_G_^2^ determines the proportion of liability in the disease that is explained by the SNP (denoted by σ_L_^2^), (c) the disease prevalence, (d) the sample size of the case-control study in which the test is performed, and (e) the type I error level.

We calculated power using the “Case-control for threshold-selected quantitative traits” module of the genetic power calculator (c.f. http://pngu.mgh.harvard.edu/~purcell/gpc/qcc.html; [37]) which approximates power in this situation, a difference being that power is calculated assuming a single equally frequent allele rather than a continuous allelic score. We calculated power to detect association using 2000 cases and 3000 controls assuming a type I error level of α = 0.05 (these conditions mimic the BMI analyses in the current manuscript and therefore provide an indication of whether significant results are likely to reflect true effects). We also investigated what our power might be if we scaled our strategy up to investigate hundreds of thousands of molecular phenotypes (e.g. 450000 methylation sites on the Illumina 450K array) in a large genome-wide meta-analysis. We therefore calculated power to detect association assuming a conservative type I error level of α = 0.05/450000 = 1.1×10^−7^ in 50000 cases and controls, which reflects the current sample size of some of the larger international GWAS consortia. We investigated the effect of varying the amount of variance the allelic score explained in the biological intermediate (σ_G_^2^ = 10%, 5%, 1%, 0.1%), the strength of relationship (i.e. linear regression coefficient) between the intermediate and underlying disease liability (β = 0.1, 0.2 or 0.5), and the prevalence of disease (K = 1%, 5%, 10%, 20%) in both scenarios.

## Supporting Information

Figure S1 Association between polygene score and BMI measured at age nine in the ALSPAC cohort before and after pruning for linkage disequilibrium. Association between polygene score and BMI measured at age nine using different p-value thresholds for the construction of the score in ALSPAC children (N = 5819). The lines joining the circles display the results for weighted allelic scores calculated by using genotyped variants from across the genome before (unbroken line) and after pruning for linkage disequilibrium (dashed line). The histogram in the background displays the number of SNPs involved in construction of the allelic score for the “All variants” condition at each corresponding SNP inclusion threshold.
(PDF)

Figure S2 Association between polygene score and CRP measured at age nine in the ALSPAC cohort before and after pruning for linkage disequilibrium. Association between polygene score and CRP measured at age nine using different p-value thresholds for the construction of the score in ALSPAC children (N = 4251). The lines joining the circles display the results for weighted allelic scores calculated by using genotyped variants from across the genome before (unbroken line) and after pruning for linkage disequilibrium (dashed line). The histogram in the background displays the number of SNPs involved in construction of the allelic score for the “All variants” condition at each corresponding SNP inclusion threshold.
(PDF)

Figure S3 Association between polygene score and LDLc measured at age nine in the ALSPAC cohort before and after pruning for linkage disequilibrium. Association between polygene score and LDLc measured at age nine using different p-value thresholds for the construction of the score in ALSPAC children (N = 4251). The lines joining the circles display the results for unweighted allelic scores calculated by using genotyped variants from across the genome before (unbroken line) and after pruning for linkage disequilibrium (dashed line). The histogram in the background displays the number of SNPs involved in construction of the allelic score for the “All variants” condition at each corresponding SNP inclusion threshold.
(PDF)

Figure S4 Association between polygene score and BMI measured at age nine in the QIMR twins replication sample. Association between polygene score and BMI using different p-value thresholds for the construction of the score in unrelated individuals from the QIMR twins cohort (N = 4781). The lines joining the circles display the results for allelic scores calculated by using genotyped variants from across the genome in either a weighted (unbroken line) or an unweighted (dashed line) fashion. The lines joining the triangles display scores calculated similarly but excluding all variants +/−1 MB around 32 known BMI variants, and using either a weighted (unbroken line) or unweighted (dashed line) strategy. The histogram in the background displays the number of SNPs involved in construction of the allelic score for the “All variants” condition at each corresponding SNP inclusion threshold.
(PDF)

Figure S5 Association between polygene score and CRP measured at age nine in the QIMR twins replication sample. Association between polygene score and CRP using different p-value thresholds for the construction of the score in unrelated individuals from the QIMR twins cohort (N = 2767). The lines joining the circles display the results for allelic scores calculated by using genotyped variants from across the genome in either a weighted (unbroken line) or an unweighted (dashed line) fashion. The lines joining the triangles display scores calculated similarly but excluding all variants +/−1 MB around 18 known CRP variants, and using either a weighted (unbroken line) or unweighted (dashed line) strategy. The histogram in the background displays the number of SNPs involved in construction of the allelic score for the “All variants” condition at each corresponding SNP inclusion threshold.
(PDF)

Figure S6 Association between polygene score and LDLc measured at age nine in the QIMR twins replication sample. Association between polygene score and LDLc using different p-value thresholds for the construction of the score in unrelated individuals from the QIMR twins cohort (N = 2630). The lines joining the circles display the results for allelic scores calculated by using genotyped variants from across the genome in either a weighted (unbroken line) or an unweighted (dashed line) fashion. The lines joining the triangles display scores calculated similarly but excluding all variants +/−1 MB around 37 known LDLc variants, and using either a weighted (unbroken line) or unweighted (dashed line) strategy. The histogram in the background displays the number of SNPs involved in construction of the allelic score for the “All variants” condition at each corresponding SNP inclusion threshold.
(PDF)

File S1 Members of the GIANT Consortium.
(PDF)

File S2 Members of the CRP Consortium.
(PDF)

File S3 Members of the TAG Consortium.
(PDF)

Table S1 Association between case-control status in the WTCCC and an allelic score that proxies for BMI.
(PDF)

Table S2 Association between case-control status in the WTCCC and an allelic score that proxies for CRP.
(PDF)

Table S3 Association between case-control status in the WTCCC and an allelic score that proxies for LDL.
(PDF)

Table S4 Association between case-control status in the WTCCC and an LD pruned allelic score that proxies for BMI.
(PDF)

Table S5 Association between case-control status in the WTCCC and an LD pruned allelic score that proxies for CRP.
(PDF)

Table S6 Association between case-control status in the WTCCC and an LD pruned allelic score that proxies for LDLc.
(PDF)

Table S7 Known SNPs contributing to the calculation of BMI, LDLc, and CRP allelic scores in ALSPAC.
(PDF)

Table S8 Known SNPs contributing to the calculation of BMI, LDLc, and CRP allelic scores in QIMR twins replication set.
(PDF)

Table S9 Performance of BMI SNPs in the CRP meta-analysis.
(PDF)
