# Supplementary figures and images for: Mining the Human Phenome Using Allelic Scores That Index Biological Intermediates
Source: PLoS Genet. 2013 Oct 31;9(10):e1003919. doi: 10.1371/journal.pgen.1003919 (PMC3814299; doi:10.1371/journal.pgen.1003919)

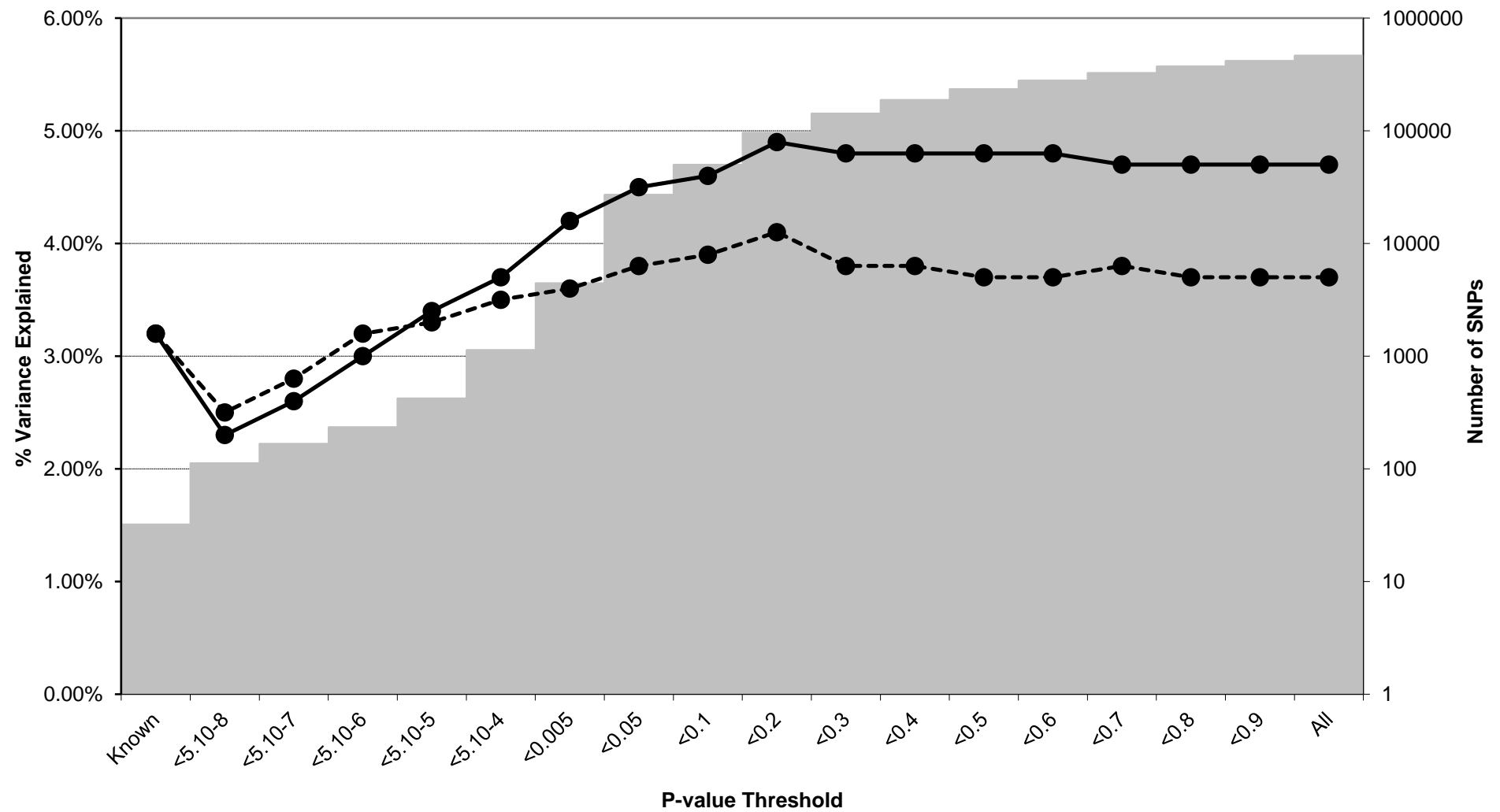

Supplement: Figure S1 — Association between polygene score and BMI measured at age nine in the ALSPAC cohort before and after pruning for linkage disequilibrium. Association between polygene score and BMI measured at age nine using different p-value thresholds for the construction of the score in ALSPAC children (N = 5819). The lines joining the circles display the results for weighted allelic scores calculated by using genotyped variants from across the genome before (unbroken line) and after pruning for linkage disequilibrium (dashed line). The histogram in the background displays the number of SNPs involved in construction of the allelic score for the “All variants” condition at each corresponding SNP inclusion threshold. (PDF) [file pgen.1003919.s001.pdf]

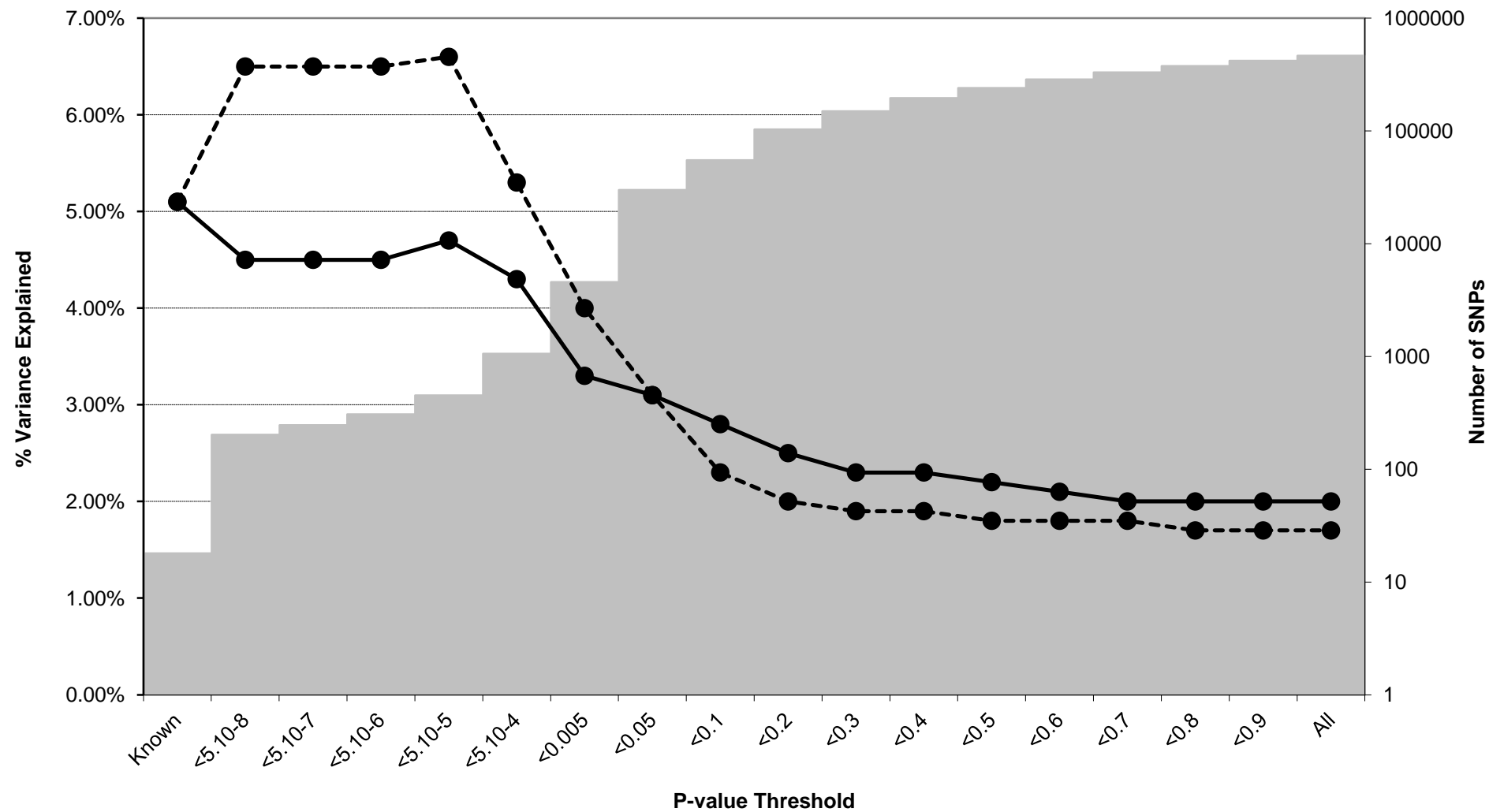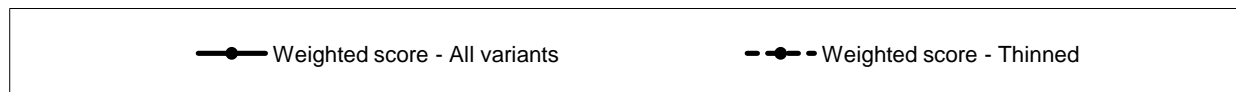

Supplement: Figure S2 — Association between polygene score and CRP measured at age nine in the ALSPAC cohort before and after pruning for linkage disequilibrium. Association between polygene score and CRP measured at age nine using different p-value thresholds for the construction of the score in ALSPAC children (N = 4251). The lines joining the circles display the results for weighted allelic scores calculated by using genotyped variants from across the genome before (unbroken line) and after pruning for linkage disequilibrium (dashed line). The histogram in the background displays the number of SNPs involved in construction of the allelic score for the “All variants” condition at each corresponding SNP inclusion threshold. (PDF) [file pgen.1003919.s002.pdf]

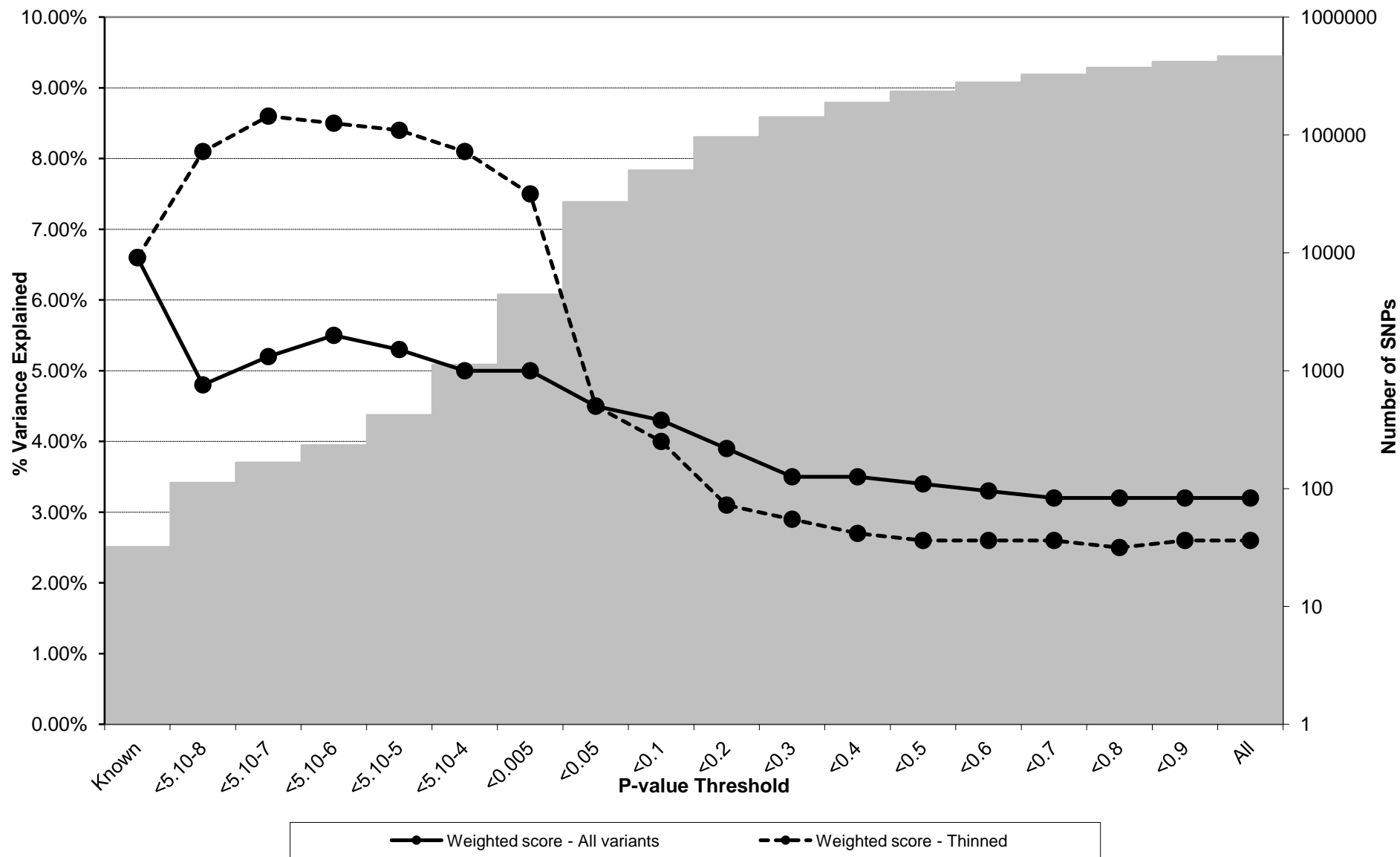

Supplement: Figure S3 — Association between polygene score and LDLc measured at age nine in the ALSPAC cohort before and after pruning for linkage disequilibrium. Association between polygene score and LDLc measured at age nine using different p-value thresholds for the construction of the score in ALSPAC children (N = 4251). The lines joining the circles display the results for unweighted allelic scores calculated by using genotyped variants from across the genome before (unbroken line) and after pruning for linkage disequilibrium (dashed line). The histogram in the background displays the number of SNPs involved in construction of the allelic score for the “All variants” condition at each corresponding SNP inclusion threshold. (PDF) [file pgen.1003919.s003.pdf]

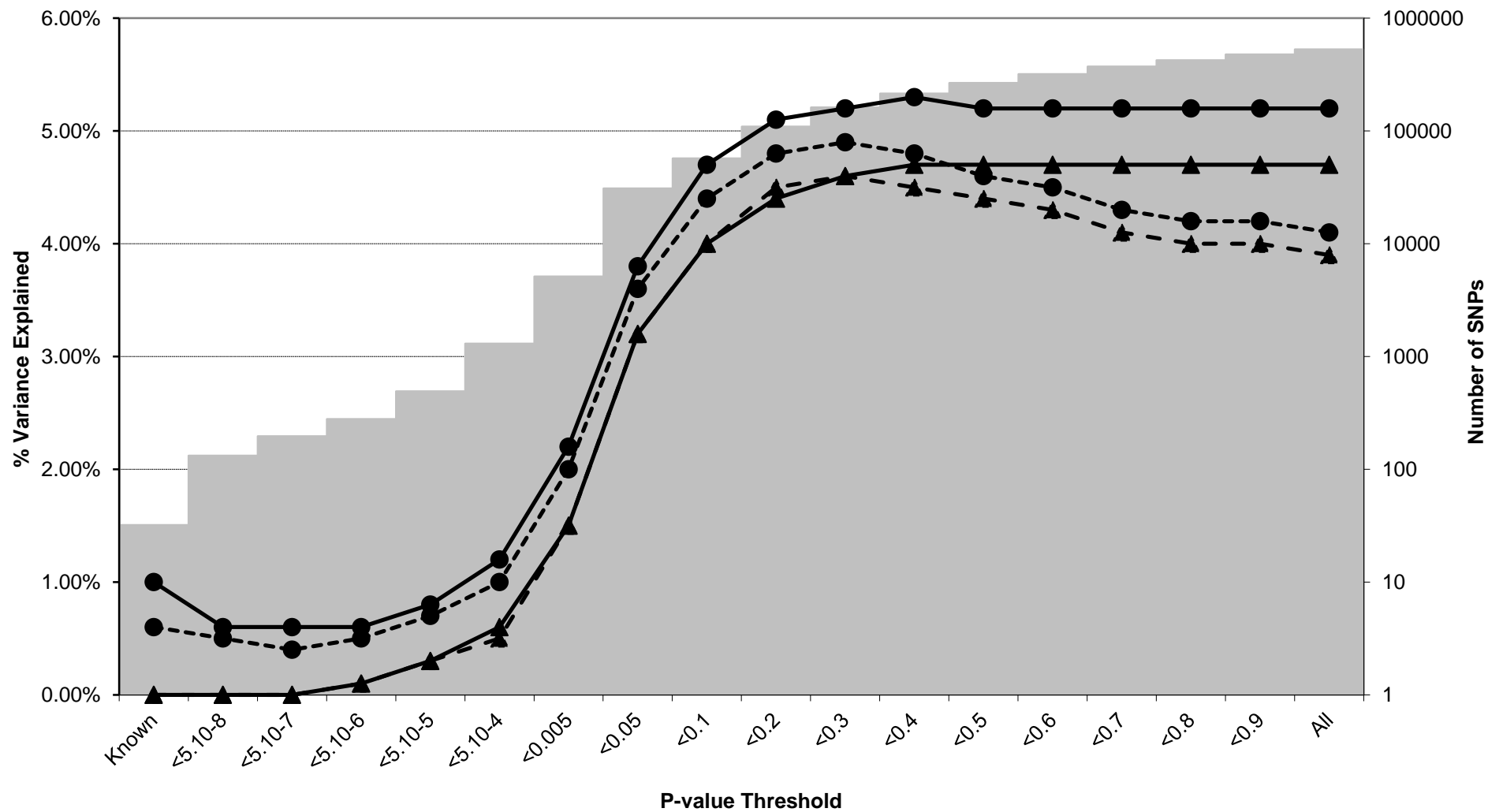

Supplement: Figure S4 — Association between polygene score and BMI measured at age nine in the QIMR twins replication sample. Association between polygene score and BMI using different p-value thresholds for the construction of the score in unrelated individuals from the QIMR twins cohort (N = 4781). The lines joining the circles display the results for allelic scores calculated by using genotyped variants from across the genome in either a weighted (unbroken line) or an unweighted (dashed line) fashion. The lines joining the triangles display scores calculated similarly but excluding all variants +/−1 MB around 32 known BMI variants, and using either a weighted (unbroken line) or unweighted (dashed line) strategy. The histogram in the background displays the number of SNPs involved in construction of the allelic score for the “All variants” condition at each corresponding SNP inclusion threshold. (PDF) [file pgen.1003919.s004.pdf]

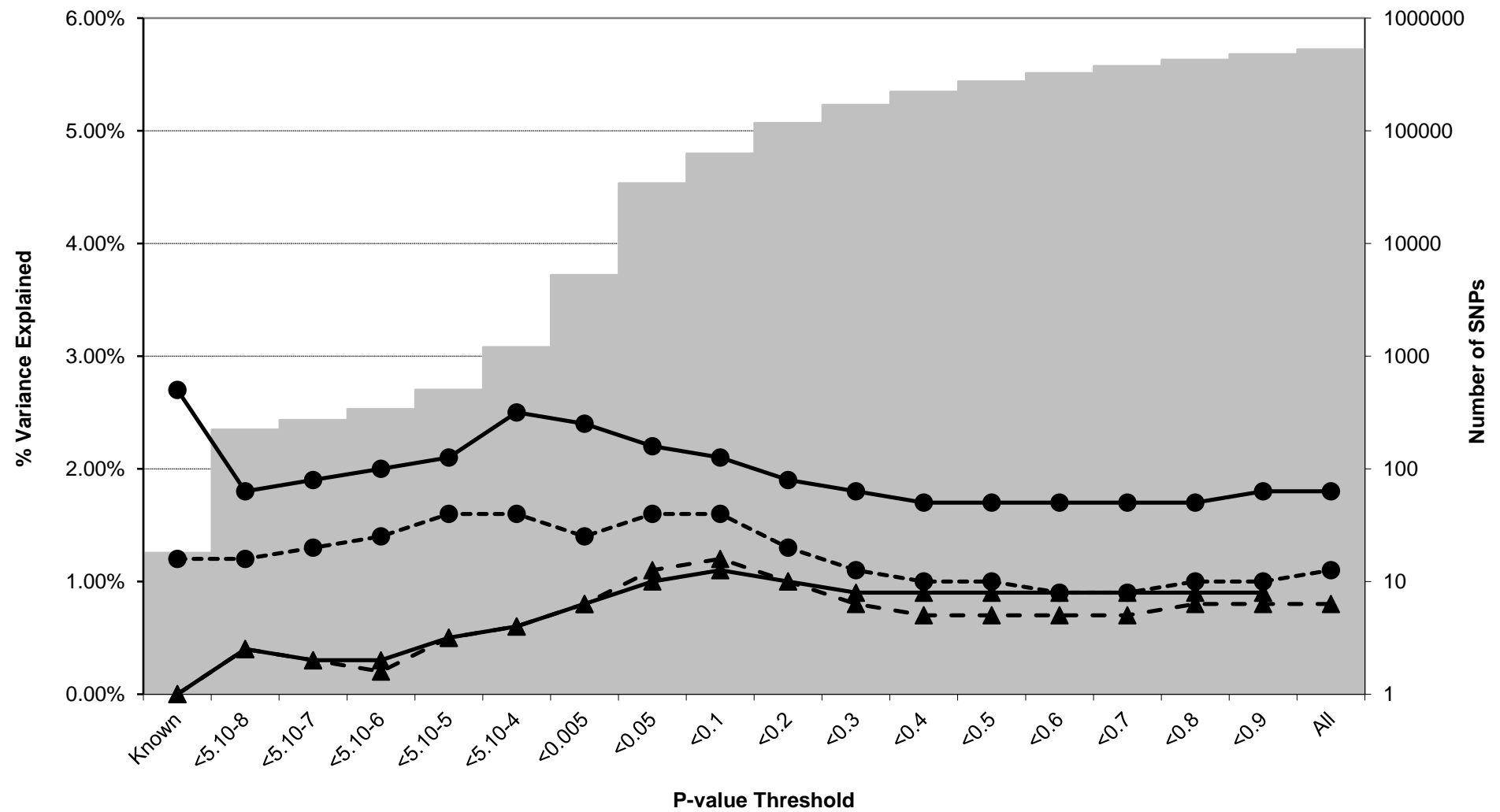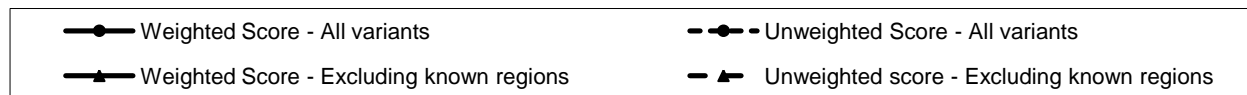

Supplement: Figure S5 — Association between polygene score and CRP measured at age nine in the QIMR twins replication sample. Association between polygene score and CRP using different p-value thresholds for the construction of the score in unrelated individuals from the QIMR twins cohort (N = 2767). The lines joining the circles display the results for allelic scores calculated by using genotyped variants from across the genome in either a weighted (unbroken line) or an unweighted (dashed line) fashion. The lines joining the triangles display scores calculated similarly but excluding all variants +/−1 MB around 18 known CRP variants, and using either a weighted (unbroken line) or unweighted (dashed line) strategy. The histogram in the background displays the number of SNPs involved in construction of the allelic score for the “All variants” condition at each corresponding SNP inclusion threshold. (PDF) [file pgen.1003919.s005.pdf]

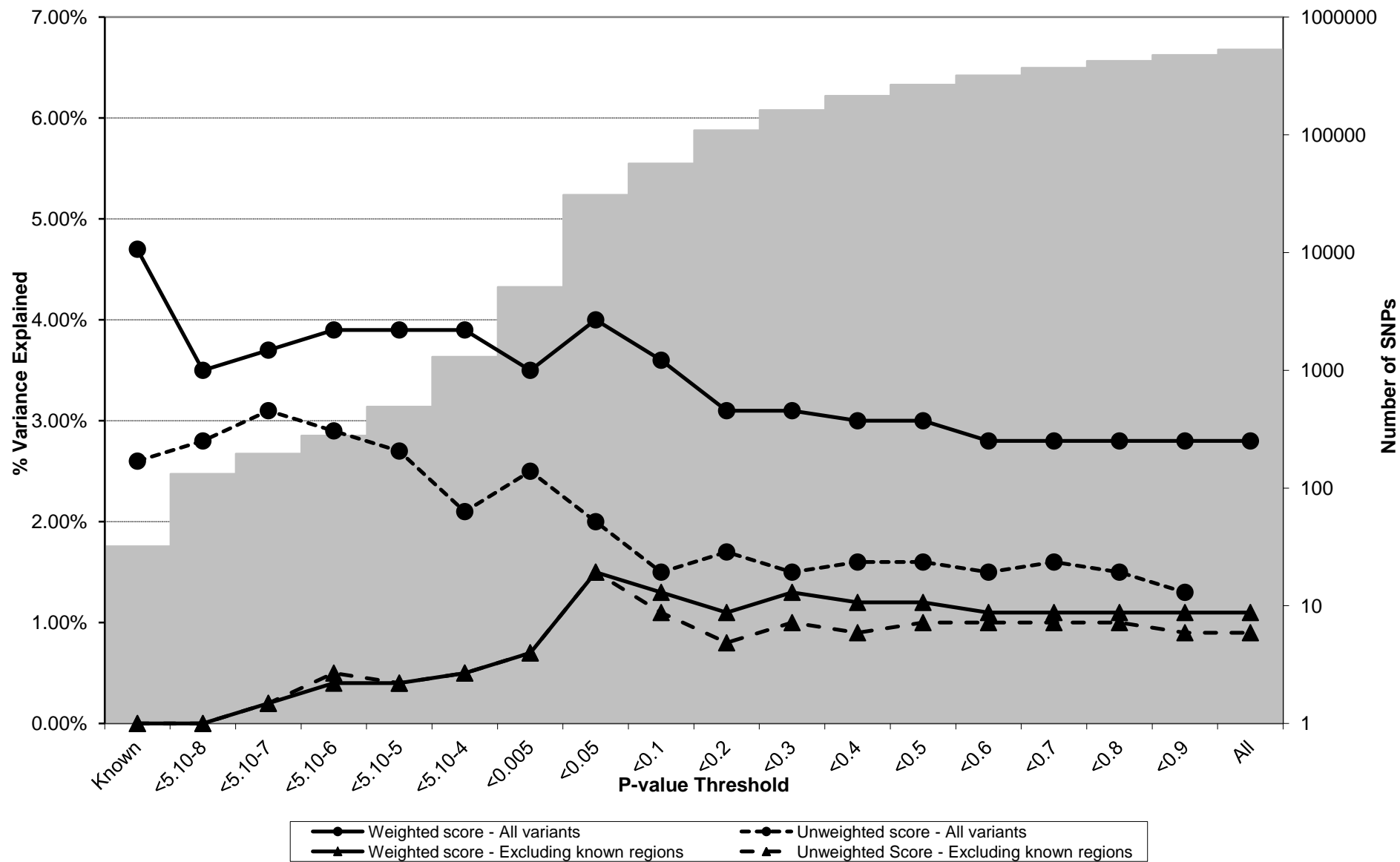

Supplement: Figure S6 — Association between polygene score and LDLc measured at age nine in the QIMR twins replication sample. Association between polygene score and LDLc using different p-value thresholds for the construction of the score in unrelated individuals from the QIMR twins cohort (N = 2630). The lines joining the circles display the results for allelic scores calculated by using genotyped variants from across the genome in either a weighted (unbroken line) or an unweighted (dashed line) fashion. The lines joining the triangles display scores calculated similarly but excluding all variants +/−1 MB around 37 known LDLc variants, and using either a weighted (unbroken line) or unweighted (dashed line) strategy. The histogram in the background displays the number of SNPs involved in construction of the allelic score for the “All variants” condition at each corresponding SNP inclusion threshold. (PDF) [file pgen.1003919.s006.pdf]
