# Supplementary material for: Mining the Human Phenome Using Allelic Scores That Index Biological Intermediates
Source: PLoS Genet. 2013 Oct 31;9(10):e1003919. doi: 10.1371/journal.pgen.1003919 (PMC3814299; doi:10.1371/journal.pgen.1003919)
Supplement: File S1 — Members of the GIANT Consortium. (PDF) [file pgen.1003919.s007.pdf]

Members of the GIANT Consortium

Elizabeth K. Speliotes[1,2], Cristen J. Willer[3], Sonja I. Berndt[4], Keri L. Monda[5], Gudmar Thorleifsson[6], Anne U. Jackson[3], Hana Lango Allen[7], Cecilia M. Lindgren[8,9], Jian'an Luan[10], Reedik Mägi[8], Joshua C. Randall[8], Sailaja Vedantam[1,11], Thomas W. Winkler[12], Lu Qi[13,14], Tsegaselassie Workalemahu[13], Iris M. Heid[12,15], Valgerdur Steinthorsdottir[6], Heather M. Stringham[3], Michael N. Weedon[7], Eleanor Wheeler[16], Andrew R. Wood[7], Teresa Ferreira[8], Robert J. Weyant[3], Ayellet V. Segré[17,18,19], Karol Estrada[20,21,22], Liming Liang[23,24], James Nemesh[18], Ju-Hyun Park[4], Stefan Gustafsson[25], Tuomas O. Kilpeläinen[10], Jian Yang[26], Nabila Bouatia-Naji[27,28], Tõnu Esko[29,30,31], Mary F. Feitosa[32], Zoltán Kutalik[33,34], Massimo Mangino[35], Soumya Raychaudhuri[18,36], Andre Scherag[37], Albert Vernon Smith[38,39], Ryan Welch[3], Jing Hua Zhao[10], Katja K. Aben[40], Devin M. Absher[41], Najaf Amin[20], Anna L. Dixon[42], Eva Fisher[43], Nicole L. Glazer[44,45], Michael E. Goddard[46,47], Nancy L. Heard-Costa[48], Volker Hoesel[49], Jouke-Jan Hottenga[50], Åsa Johansson[51,52], Toby Johnson[33,34,53,54], Shamika Ketkar[32], Claudia Lamina[15,55], Shengxu Li[10], Miriam F. Moffatt[56], Richard H. Myers[57], Narisu Narisu[58], John R.B. Perry[7], Marjolein J. Peters[21,22], Michael Preuss[59], Samuli Ripatti[60,61], Fernando Rivadeneira[20,21,22], Camilla Sandholt[62], Laura J. Scott[3], Nicholas J. Timpson[63], Jonathan P. Tyrer[64], Sophie van Wingerden[20], Richard M. Watanabe[65,66], Charles C. White[67], Fredrik Wiklund[25], Christina Barlassina[68], Daniel I. Chasman[69,70], Matthew N. Cooper[71], John-Olov Jansson[72], Robert W. Lawrence[71], Niina Pellikka[60,61], Inga Prokopenko[8,9], Jianxin Shi[4], Elisabeth Thiering[15], Helene Alavere[29], Maria T. S. Alibrandi[73], Peter Almgren[74], Alice M. Arnold[75,76], Thor Aspelund[38,39], Larry D. Atwood[48], Beverley Balkau[77,78], Anthony J. Balmforth[79], Amanda J. Bennett[9], Yoav Ben-Shlomo[80], Richard N. Bergman[66], Sven Bergmann[33,34], Heike Biebermann[81], Alexandra I.F. Blakemore[82], Tanja Boes[37], Lori L. Bonnycastle[58], Stefan R. Bornstein[83], Morris J. Brown[84], Thomas A. Buchanan[66,85], Fabio Busonero[86], Harry Campbell[87], Francesco P. Cappuccio[88], Christine Cavalcanti-Proença[27,28], Yii-Der Ida Chen[89], Chih-Mei Chen[15], Peter S. Chines[58], Robert Clarke[90], Lachlan Coin[91], John Connell[92], Ian N.M. Day[63], Martin den Heijer[93,94], Jubao Duan[95], Shah Ebrahim[96,97], Paul Elliott[91,98], Roberto Elosua[99], Gudny Eiriksdottir[38], Michael R. Erdos[58], Johan G. Eriksson[100,101,102,103,104], Maurizio F. Facheris[105,106], Stephan B. Felix[107], Pamela Fischer-Posovszky[108], Aaron R. Folsom[109], Nele Friedrich[110], Nelson B. Freimer[111], Mao Fu[112], Stefan Gaget[27,28], Pablo V. Gejman[95], Eco J.C. Geus[50], Christian Gieger[15], Anette P. Gjesing[62], Anuj Goel[8,113], Philippe Goyette[114], Harald Grallert[15], Jürgen Gräßler[115], Danielle M. Greenawalt[116], Christopher J. Groves[9], Vilmundur Gudnason[38,39], Candace Guiducci[1], Anna-Liisa Hartikainen[117], Neelam Hassanali[9], Alistair S. Hall[79], Aki S. Havulinna[118], Caroline Hayward[119], Andrew C. Heath[120], Christian Hengstenberg[121,122], Andrew A. Hicks[105], Anke Hinney[123], Albert Hofman[20,22], Georg Homuth[124], Jennie Hui[71,125,126], Wilmar Igl[51], Carlos Iribarren[127,128], Bo Isomaa[103,129], Kevin B. Jacobs[130], Ivonne Jarick[131], Elizabeth Jewell[3], Ulrich John[132], Torben Jørgensen[133,134], Pekka Jousilahti[118], Antti Jula[135], Marika Kaakinen[136,137], Eero Kajantie[101,138], Lee M. Kaplan[2,70,139], Sekar Kathiresan[17,18,140,141,142], Johannes Kettunen[60,61], Leena Kinnunen[143], Joshua W. Knowles[144], Ivana Kolcic[145], Inke R. König[59], Seppo Koskinen[118], Peter Kovacs[146], Johanna Kuusisto[147], Peter Kraft[23,24], Kirsti Kvaløy[148], Jaana Laitinen[149], Olivier Lantieri[150], Chiara Lanzani[73], Lenore J. Launer[151], Cecile Lecoeur[27,28], Terho Lehtimäki[152], Guillaume Lettre[114,153], Jianjun Liu[154], Marja-Liisa Lokki[155], Mattias Lorentzon[156], Robert N. Luben[157], Barbara Ludwig[83], MAGIC[158], Paolo Manunta[73], Diana Marek[33,34], Michel Marre[159,160], Nicholas G. Martin[161], Wendy L. McArdle[162], Anne McCarthy[163], Barbara McKnight[75], Thomas Meitinger[164,165], Olle Melander[166], David Meyre[27,28], Kristian Midthjell[148], Grant W. Montgomery[167], Mario A. Morken[58], Andrew P. Morris[8], Rosanda Mulic[168], Julius S. Ngwa[67], Mari Nelis[29,30,31], Matt J. Neville[9], Dale R. Nyholt[169], Christopher J. O'Donnell[141,170], Stephen O'Rahilly[171], Ken K. Ong[10], Ben Oostra[172], Guillaume Paré[173], Alex N. Parker[174], Markus Perola[60,61], Irene Pichler[105], Kirsi H. Pietiläinen[175,176], Carl G.P. Platou[148,177], Ozren Polasek[145,178], Anneli Pouta[117,179], Suzanne Rafelt[180], Olli Raitakari[181,182], Nigel W. Rayner[8,9], Martin Ridderstråle[166], Winfried Rief[183], Aimo Ruokonen[184], Neil R. Robertson[8,9], Peter Rzehak[15,185], Veikko Salomaa[118], Alan R. Sanders[95], Manjinder S. Sandhu[10,16,157], Serena Sanna[86], Jouko Saramies[186], Markku J. Savolainen[187], Susann Scherag[123], Sabine Schipf[110,188], Stefan Schreiber[189], Heribert Schunkert[190], Kaisa Silander[60,61], Juha Sinisalo[191], David S. Siscovick[45,192], Jan H. Smit[193], Nicole Soranzo[16,35], Ulla Sovio[91], Jonathan Stephens[194,195], Ida Surakka[60,61], Amy J. Swift[58], Mari-Liis Tammesoo[29], Jean-Claude Tardif[114,153], Maris Teder-Laving[30,31], Tanya M. Teslovich[3], John R. Thompson[196,197], Brian Thomson[1], Anke Tönjes[198,199], Tiinamaija Tuomi[103,200,201], Joyce B.J.

van Meurs[20,21,22], **Gert-Jan van Ommen**[202,203], **Vincent Vatin**[27,28], **Jorma Viikari**[204], **Sophie Visvikis-Siest**[205], **Veronique Vitart**[119], **Carla I. G. Vogel**[123], **Benjamin F. Voight**[17,18,19], **Lindsay L. Waite**[41], **Henri Wallaschofski**[110], **G. Bragi Walters**[6], **Elisabeth Widen**[60], **Susanna Wiegand**[81], **Sarah H. Wild**[87], **Gonneke Willemsen**[50], **Daniel R. Witte**[206], **Jacqueline C. Witteman**[20,22], **Jianfeng Xu**[207], **Qunyuan Zhang**[32], **Lina Zgaga**[145], **Andreas Ziegler**[59], **Paavo Zitting**[208], **John P. Beilby**[125,126,209], **I. Sadaf Farooqi**[171], **Johannes Hebebrand**[123], **Heikki V. Huikuri**[210,210], **Alan L. James**[126,211], **Mika Kähönen**[212], **Douglas F. Levinson**[213], **Fabio Macciardi**[68,214], **Markku S. Nieminen**[191,191], **Claes Ohlsson**[156], **Lyle J. Palmer**[71,126], **Paul M. Ridker**[69,70], **Michael Stumvoll**[198,215], **Jacques S. Beckmann**[33,216], **Heiner Boeing**[43], **Eric Boerwinkle**[217], **Dorret I. Boomsma**[50], **Mark J. Caulfield**[54], **Stephen J. Chanock**[4], **Francis S. Collins**[58], **L. Adrienne Cupples**[67], **George Davey Smith**[63], **Jeanette Erdmann**[190], **Philippe Froguel**[27,28,82], **Henrik Grönberg**[25], **Ulf Gyllensten**[51], **Per Hall**[25], **Torben Hansen**[62,218], **Tamara B. Harris**[151], **Andrew T. Hattersley**[7], **Richard B. Hayes**[219], **Joachim Heinrich**[15], **Frank B. Hu**[13,14,23], **Kristian Hveem**[148], **Thomas Illig**[15], **Marjo-Riitta Jarvelin**[91,136,137,179], **Jaakko Kaprio**[60,175,220], **Fredrik Karpe**[9,221], **Kay-Tee Khaw**[157], **Lambertus A. Kiemeney**[40,93,222], **Heiko Krude**[81], **Markku Laakso**[147], **Debbie A. Lawlor**[63], **Andres Metspalu**[29,30,31], **Patricia B. Munroe**[54], **Willem H. Ouwehand**[16,194,195], **Oluf Pedersen**[62,223,224], **Brenda W. Penninx**[193,225,226], **Annette Peters**[15], **Peter P. Pramstaller**[105,106,227], **Thomas Quertermous**[144], **Thomas Reinehr**[228], **Aila Rissanen**[176], **Igor Rudan**[87,168], **Nilesh J. Samani**[180,196], **Peter E.H. Schwarz**[229], **Alan R. Shuldiner**[112,230], **Timothy D. Spector**[35], **Jaakko Tuomilehto**[143,231,232], **Manuela Uda**[86], **André Uitterlinden**[20,21,22], **Timo T. Valle**[143], **Martin Wabitsch**[108], **Gérard Waeber**[233], **Nicholas J. Wareham**[10], **Hugh Watkins**[8,113], **James F. Wilson**[87], **Alan F. Wright**[119], **M. Carola Zillikens**[21,22], **Nilanjan Chatterjee**[4], **Steven A. McCarroll**[17,18,19], **Shaun Purcell**[17,234,235], **Eric E. Schadt**[236,237], **Peter M. Visscher**[26], **Themistocles L. Assimes**[144], **Ingrid B. Borecki**[32,238], **Panos Deloukas**[16], **Caroline S. Fox**[239], **Leif C. Groop**[74], **Talin Haritunians**[89], **David J. Hunter**[13,14,23], **Robert C. Kaplan**[240], **Karen L. Mohlke**[241], **Jeffrey R. O'Connell**[112], **Leena Peltonen**[16,60,61,234,242], **David Schlessinger**[243], **David P. Strachan**[244], **Cornelia M. Van Duijn**[20,22], **H.-Erich Wichmann**[15,185,245], **Timothy M. Frayling**[7], **Unnur Thorsteinsdottir**[6,246], **Gonçalo R. Abecasis**[3], **Inês Barroso**[16,247], **Michael Boehnke**[3], **Kari Stefansson**[6,246,*], **Kari E. North**[5,248], **Mark I. McCarthy**[8,9,221], **Joel N. Hirschhorn**[1,11,249], **Erik Ingelsson**[25], and **Ruth J.F. Loos**

[1] Metabolism Initiative and Program in Medical and Population Genetics, Broad Institute, Cambridge, Massachusetts 02142, USA

[2] Division of Gastroenterology, Massachusetts General Hospital, Boston, Massachusetts 02114, USA

[3] Department of Biostatistics, Center for Statistical Genetics, University of Michigan, Ann Arbor, Michigan 48109, USA

[4] Division of Cancer Epidemiology and Genetics, National Cancer Institute, National Institutes of Health, Department of Health and Human Services, Bethesda, Maryland 20892, USA

[5] Department of Epidemiology, School of Public Health, University of North Carolina at Chapel Hill, Chapel Hill, North Carolina 27514, USA

[6] deCODE Genetics, 101 Reykjavik, Iceland

[7] Genetics of Complex Traits, Peninsula College of Medicine and Dentistry, University of Exeter, Exeter, EX1 2LU, UK

[8] Wellcome Trust Centre for Human Genetics, University of Oxford, Oxford, OX3 7BN, UK

[9] Oxford Centre for Diabetes, Endocrinology and Metabolism, University of Oxford, Oxford, OX3 7LJ, UK

[10] MRC Epidemiology Unit, Institute of Metabolic Science, Addenbrooke's Hospital, Cambridge, CB2 0QQ, UK

[11] Divisions of Genetics and Endocrinology and Program in Genomics, Children's Hospital, Boston, Massachusetts 02115, USA

[12] Regensburg University Medical Center, Department of Epidemiology and Preventive Medicine, 93053 Regensburg, Germany

[13] Department of Nutrition, Harvard School of Public Health, Boston, Massachusetts 02115, USA
[14] Channing Laboratory, Department of Medicine, Brigham and Women's Hospital and Harvard Medical School, Boston, Massachusetts 02115, USA
[15] Institute of Epidemiology, Helmholtz Zentrum München - German Research Center for Environmental Health, 85764 Neuherberg, Germany
[16] Wellcome Trust Sanger Institute, Hinxton, Cambridge, CB10 1SA, UK
[17] Center for Human Genetic Research, Massachusetts General Hospital, Boston, Massachusetts 02114, USA
[18] Program in Medical and Population Genetics, Broad Institute of Harvard and Massachusetts Institute of Technology, Cambridge, Massachusetts 02142, USA
[19] Department of Molecular Biology, Massachusetts General Hospital, Boston, Massachusetts 02114, USA
[20] Department of Epidemiology, Erasmus MC, Rotterdam, 3015GE, The Netherlands
[21] Department of Internal Medicine, Erasmus MC, Rotterdam, 3015GE, The Netherlands
[22] Netherlands Genomics Initiative (NGI)-sponsored Netherlands Consortium for Healthy Aging (NCHA)
[23] Department of Epidemiology, Harvard School of Public Health, Boston, Massachusetts 02115, USA
[24] Department of Biostatistics, Harvard School of Public Health, Boston, Massachusetts 02115, USA
[25] Department of Medical Epidemiology and Biostatistics, Karolinska Institutet, 171 77 Stockholm, Sweden
[26] Queensland Statistical Genetics Laboratory, Queensland Institute of Medical Research, Queensland 4006, Australia
[27] CNRS UMR8199-IBL-Institut Pasteur de Lille, F-59019 Lille, France
[28] University Lille Nord de France, 59000 Lille, France
[29] Estonian Genome Center, University of Tartu, Tartu 50410, Estonia
[30] Estonian Biocenter, Tartu 51010, Estonia
[31] Institute of Molecular and Cell Biology, University of Tartu, Tartu 51010, Estonia
[32] Department of Genetics, Washington University School of Medicine, St Louis, Missouri 63110, USA
[33] Department of Medical Genetics, University of Lausanne, 1005 Lausanne, Switzerland
[34] Swiss Institute of Bioinformatics, 1015 Lausanne, Switzerland
[35] Department of Twin Research and Genetic Epidemiology, King's College London, London, SE1 7EH, UK
[36] Division of Rheumatology, Immunology and Allergy, Brigham and Women's Hospital, Harvard Medical School, Boston, Massachusetts 02115 USA
[37] Institute for Medical Informatics, Biometry and Epidemiology, University of Duisburg-Essen, 45122 Essen, Germany
[38] Icelandic Heart Association, Kopavogur, Iceland
[39] University of Iceland, Reykjavik, Iceland
[40] Comprehensive Cancer Center East, 6501 BG Nijmegen, The Netherlands
[41] Hudson Alpha Institute for Biotechnology, Huntsville, Alabama 35806, USA
[42] Department of Pharmacy and Pharmacology, University of Bath, Bath, BA1 1RL, UK
[43] Department of Epidemiology, German Institute of Human Nutrition Potsdam-Rehbruecke, 14558 Nuthetal, Germany
[44] Department of Medicine, University of Washington, Seattle, Washington 98101, USA
[45] Cardiovascular Health Research Unit, University of Washington, Seattle, Washington 98101, USA

[46] University of Melbourne, Parkville 3010, Australia
[47] Department of Primary Industries, Melbourne, Victoria 3001, Australia
[48] Department of Neurology, Boston University School of Medicine, Boston, Massachusetts 02118, USA
[49] Technical University Munich, Chair of Biomathematics, Boltzmannstrasse 3, 85748 Garching
[50] Department of Biological Psychology, VU University Amsterdam, 1081 BT Amsterdam, The Netherlands
[51] Department of Genetics and Pathology, Rudbeck Laboratory, University of Uppsala, SE-75185 Uppsala, Sweden
[52] Department of Cancer Research and Molecular Medicine, Faculty of Medicine, Norwegian University of Science and Technology (NTNU), Trondheim, N-7489, Norway
[53] Clinical Pharmacology, William Harvey Research Institute, Barts and The London School of Medicine and Dentistry, Queen Mary, University of London, London, UK
[54] Clinical Pharmacology and Barts and The London Genome Centre, William Harvey Research Institute, Barts and The London School of Medicine and Dentistry, Queen Mary University of London, Charterhouse Square, London EC1M 6BQ, UK
[55] Division of Genetic Epidemiology, Department of Medical Genetics, Molecular and Clinical Pharmacology, Innsbruck Medical University, 6020 Innsbruck, Austria
[56] National Heart and Lung Institute, Imperial College London, London SW3 6LY, UK
[57] Department of Neurology, Boston University School of Medicine, Boston, Massachusetts 02118, USA
[58] National Human Genome Research Institute, National Institutes of Health, Bethesda, Maryland 20892, USA
[59] Institut fur Medizinische Biometrie und Statistik, Universitat zu Lubeck, Universitatsklinikum Schleswig-Holstein, Campus Lubeck, 23562 Lubeck, Germany
[60] Institute for Molecular Medicine Finland (FIMM), University of Helsinki, 00014, Helsinki, Finland
[61] National Institute for Health and Welfare, Department of Chronic Disease Prevention, Unit of Public Health Genomics, 00014, Helsinki, Finland
[62] Hagedorn Research Institute, 2820 Gentofte, Denmark
[63] MRC Centre for Causal Analyses in Translational Epidemiology, Department of Social Medicine, Oakfield House, Bristol, BS8 2BN, UK
[64] Department of Oncology, University of Cambridge, Cambridge, CB1 8RN, UK
[65] Department of Preventive Medicine, Keck School of Medicine, University of Southern California, Los Angeles, California 90089, USA
[66] Department of Physiology and Biophysics, Keck School of Medicine, University of Southern California, Los Angeles, California 90033, USA
[67] Department of Biostatistics, Boston University School of Public Health, Boston, Massachusetts 02118, USA
[68] University of Milan, Department of Medicine, Surgery and Dentistry, 20139 Milano, Italy
[69] Division of Preventive Medicine, Brigham and Women's Hospital, Boston, Massachusetts 02215, USA
[70] Harvard Medical School, Boston, Massachusetts 02115, USA
[71] Centre for Genetic Epidemiology and Biostatistics, University of Western Australia, Crawley, Western Australia 6009, Australia
[72] Department of Physiology, Institute of Neuroscience and Physiology, Sahlgrenska Academy, University of Gothenburg, 405 30 Gothenburg, Sweden
[73] University Vita-Salute San Raffaele, Division of Nephrology and Dialysis, 20132 Milan, Italy

[74] Lund University Diabetes Centre, Department of Clinical Sciences, Lund University, 20502 Malmö, Sweden
[75] Departments of Biostatistics, University of Washington, Seattle, Washington 98195, USA
[76] Collaborative Health Studies Coordinating Center, Seattle, Washington 98115, USA
[77] INSERM CESP Centre for Research in Epidemiology and Public Health U1018, Epidemiology of diabetes, obesity and chronic kidney disease over the lifecourse, 94807 Villejuif, France
[78] University Paris Sud 11, UMRS 1018, 94807 Villejuif, France
[79] Multidisciplinary Cardiovascular Research Centre (MCRC), Leeds Institute of Genetics, Health and Therapeutics (LIGHT), University of Leeds, Leeds LS2 9JT, UK
[80] Department of Social Medicine, University of Bristol, Bristol, BS8 2PS, UK
[81] Institute of Experimental Paediatric Endocrinology, Charite Universitatsmedizin Berlin, 13353 Berlin, Germany
[82] Department of Genomics of Common Disease, School of Public Health, Imperial College London, W12 0NN, London, UK
[83] Department of Medicine III, University of Dresden, 01307 Dresden, Germany
[84] Clinical Pharmacology Unit, University of Cambridge, Addenbrooke's Hospital, Hills Road, Cambridge CB2 2QQ, UK
[85] Division of Endocrinology, Keck School of Medicine, University of Southern California, Los Angeles, California 90033, USA
[86] Istituto di Neurogenetica e Neurofarmacologia del CNR, Monserrato, 09042, Cagliari, Italy
[87] Centre for Population Health Sciences, University of Edinburgh, Teviot Place, Edinburgh, EH8 9AG, Scotland
[88] University of Warwick, Warwick Medical School, Coventry, CV2 2DX, UK
[89] Medical Genetics Institute, Cedars-Sinai Medical Center, Los Angeles, California 90048, USA
[90] Clinical Trial Service Unit, Richard Doll Building, Old Road Campus, Roosevelt Drive, Oxford, OX3 7LF, UK
[91] Department of Epidemiology and Biostatistics, School of Public Health, Faculty of Medicine, Imperial College London, London, W2 1PG, UK
[92] University of Dundee, Ninewells Hospital & Medical School, Dundee, DD1 9SY, UK
[93] Department of Epidemiology, Biostatistics and HTA, Radboud University Nijmegen Medical Centre, 6500 HB Nijmegen, The Netherlands
[94] Department of Endocrinology, Radboud University Nijmegen Medical Centre, 6500 HB Nijmegen, The Netherlands
[95] Northshore University Healthsystem, Evanston, Ilinois 60201, USA
[96] The London School of Hygiene and Tropical Medicine, London, WC1E 7HT, UK
[97] South Asia Network for Chronic Disease
[98] MRC-HPA Centre for Environment and Health, London W2 1PG, UK
[99] Cardiovascular Epidemiology and Genetics, Institut Municipal D'investigacio Medica and CIBER Epidemiologia y Salud Publica, Barcelona, Spain
[100] Department of General Practice and Primary health Care, University of Helsinki, Helsinki, Finland
[101] National Institute for Health and Welfare, 00271 Helsinki, Finland
[102] Helsinki University Central Hospital, Unit of General Practice, 00280 Helsinki, Finland
[103] Folkhalsan Research Centre, 00250 Helsinki, Finland
[104] Vasa Central Hospital, 65130 Vasa, Finland
[105] Institute of Genetic Medicine, European Academy Bozen/Bolzano (EURAC), Bolzano/Bozen, 39100, Italy. Affiliated Institute of the University of Lubeck, Lubeck, Germany

[106] Department of Neurology, General Central Hospital, Bolzano, Italy
[107] Department of Internal Medicine B, Ernst-Moritz-Arndt University, 17475 Greifswald, Germany
[108] Pediatric Endocrinology, Diabetes and Obesity Unit, Department of Pediatrics and Adolescent Medicine, 89075 Ulm, Germany
[109] Division of Epidemiology and Community Health, School of Public Health, University of Minnesota, Minneapolis Minnesota 55454, USA
[110] Institut fur Klinische Chemie und Laboratoriumsmedizin, Universitat Greifswald, 17475 Greifswald, Germany
[111] Center for Neurobehavioral Genetics, University of California, Los Angeles, California 90095, USA
[112] Department of Medicine, University of Maryland School of Medicine, Baltimore, Maryland 21201, USA
[113] Department of Cardiovascular Medicine, University of Oxford, Level 6 West Wing, John Radcliffe Hospital, Headley Way, Headington, Oxford, OX3 9DU
[114] Montreal Heart Institute, Montreal, Quebec, H1T 1C8, Canada
[115] Department of Medicine III, Pathobiochemistry, University of Dresden, 01307 Dresden, Germany
[116] Merck Research Laboratories, Merck & Co., Inc., Boston, Massachusetts 02115, USA
[117] Department of Clinical Sciences/Obstetrics and Gynecology, University of Oulu, 90014 Oulu, Finland
[118] National Institute for Health and Welfare, Department of Chronic Disease Prevention, Chronic Disease Epidemiology and Prevention Unit, 00014, Helsinki, Finland
[119] MRC Human Genetics Unit, Institute for Genetics and Molecular Medicine, Western General Hospital, Edinburgh, EH4 2XU, Scotland, UK
[120] Department of Psychiatry and Midwest Alcoholism Research Center, Washington University School of Medicine, St Louis, Missouri 63108, USA
[121] Klinik und Poliklinik fur Innere Medizin II, Universitat Regensburg, 93053 Regensburg, Germany
[122] Regensburg University Medical Center, Innere Medizin II, 93053 Regensburg, Germany
[123] Department of Child and Adolescent Psychiatry, University of Duisburg-Essen, 45147 Essen, Germany
[124] Interfaculty Institute for Genetics and Functional Genomics, Ernst-Moritz-Arndt-University Greifswald, 17487 Greifswald, Germany
[125] PathWest Laboratory of Western Australia, Department of Molecular Genetics, J Block, QEII Medical Centre, Nedlands, Western Australia 6009, Australia
[126] Busselton Population Medical Research Foundation Inc., Sir Charles Gairdner Hospital, Nedlands, Western Australia 6009, Australia
[127] Division of Research, Kaiser Permanente Northern California, Oakland, California 94612, USA
[128] Department of Epidemiology and Biostatistics, University of California, San Francisco, San Francisco, California 94107, USA
[129] Department of Social Services and Health Care, 68601 Jakobstad, Finland
[130] Core Genotyping Facility, SAIC-Frederick, Inc., NCI-Frederick, Frederick, Maryland 21702, USA
[131] Institute of Medical Biometry and Epidemiology, University of Marburg, 35037 Marburg, Germany
[132] Institut fur Epidemiologie und Sozialmedizin, Universitat Greifswald, 17475 Greifswald, Germany

[133] Research Centre for Prevention and Health, Glostrup University Hospital, 2600 Glostrup, Denmark
[134] Faculty of Health Science, University of Copenhagen, 2100 Copenhagen, Denmark
[135] National Institute for Health and Welfare, Department of Chronic Disease Prevention, Population Studies Unit, 20720 Turku, Finland
[136] Institute of Health Sciences, University of Oulu, 90014 Oulu, Finland
[137] Biocenter Oulu, University of Oulu, 90014 Oulu, Finland
[138] Hospital for Children and Adolescents, Helsinki University Central Hospital and University of Helsinki, 00029 HUS, Finland
[139] MGH Weight Center, Massachusetts General Hospital, Boston, Massachusetts 02114, USA
[140] Cardiovascular Research Center and Cardiology Division, Massachusetts General Hospital, Boston, Massachusetts 02114, USA
[141] Framingham Heart Study of the National, Heart, Lung, and Blood Institute and Boston University, Framingham, Massachusetts 01702, USA
[142] Department of Medicine, Harvard Medical School, Boston, Massachusetts 02115, USA
[143] National Institute for Health and Welfare, Diabetes Prevention Unit, 00271 Helsinki, Finland
[144] Department of Medicine, Stanford University School of Medicine, Stanford, California 94305, USA
[145] Andrija Stampar School of Public Health, Medical School, University of Zagreb, 10000 Zagreb, Croatia
[146] Interdisciplinary Centre for Clinical Research, University of Leipzig, 04103 Leipzig, Germany
[147] Department of Medicine, University of Kuopio and Kuopio University Hospital, 70210 Kuopio, Finland
[148] HUNT Research Centre, Department of Public Health and General Practice, Norwegian University of Science and Technology, 7600 Levanger, Norway
[149] Finnish Institute of Occupational Health, 90220 Oulu, Finland
[150] Institut inter-regional pour la sante (IRSA), F-37521 La Riche, France
[151] Laboratory of Epidemiology, Demography, Biometry, National Institute on Aging, National Institutes of Health, Bethesda, Maryland 20892, USA
[152] Department of Clinical Chemistry, University of Tampere and Tampere University Hospital, 33520 Tampere, Finland
[153] Department of Medicine, Universite de Montreal, Montreal, Quebec, H3T 1J4, Canada
[154] Human Genetics, Genome Institute of Singapore, Singapore 138672, Singapore
[155] Transplantation Laboratory, Haartman Institute, University of Helsinki, 00014, Helsinki, Finland
[156] Department of Internal Medicine, Institute of Medicine, Sahlgrenska Academy, University of Gothenburg, 413 45 Gothenburg, Sweden
[157] Department of Public Health and Primary Care, Institute of Public Health, University of Cambridge, Cambridge CB2 2SR, UK
[158] On behalf of the MAGIC (Meta-Analyses of Glucose and Insulin-related traits Consortium) investigators
[159] Department of Endocrinology, Diabetology and Nutrition, Bichat-Claude Bernard University Hospital, Assistance Publique des Hopitaux de Paris, F-75018 Paris, France
[160] Cardiovascular Genetics Research Unit, Universite Henri Poincare-Nancy 1, 54000, Nancy, France
[161] Genetic Epidemiology Laboratory, Queensland Institute of Medical Research, Queensland 4006, Australia

[162] Avon Longitudinal Study of Parents and Children (ALSPAC) Laboratory, Department of Social Medicine, University of Bristol, Bristol, BS8 2BN, UK
[163] Division of Health, Research Board, An Bord Taighde Slainte, Dublin, 2, Ireland
[164] Institute of Human Genetics, Klinikum rechts der Isar der Technischen Universitat Munchen, 81675 Munich, Germany
[165] Institute of Human Genetics, Helmholtz Zentrum Munchen - German Research Center for Environmental Health, 85764 Neuherberg, Germany
[166] Department of Clinical Sciences, Lund University, 20502 Malmo, Sweden
[167] Molecular Epidemiology Laboratory, Queensland Institute of Medical Research, Queensland 4006, Australia
[168] Croatian Centre for Global Health, School of Medicine, University of Split, Split 21000, Croatia
[169] Neurogenetics Laboratory, Queensland Institute of Medical Research, Queensland 4006, Australia
[170] National, Lung, and Blood Institute, National Institutes of Health, Framingham, Massachusetts 01702, USA
[171] University of Cambridge Metabolic Research Laboratories, Institute of Metabolic Science, Addenbrooke's Hospital, Cambridge CB2 0QQ, UK
[172] Department of Clinical Genetics, Erasmus MC, Rotterdam, 3015GE, The Netherlands
[173] Department of Pathology and Molecular Medicine, McMaster University, Hamilton, Ontario L8N3Z5, Canada
[174] Amgen, Cambridge, Massachusetts 02139, USA
[175] Finnish Twin Cohort Study, Department of Public Health, University of Helsinki, 00014, Helsinki, Finland
[176] Obesity Research unit, Department of Psychiatry, Helsinki University Central Hospital, Helsinki, Finland
[177] Department of Medicine, Levanger Hospital, The Nord-Trøndelag Health Trust, 7600 Levanger, Norway
[178] Gen-Info Ltd, 10000 Zagreb, Croatia
[179] National Institute for Health and Welfare, 90101 Oulu, Finland
[180] Department of Cardiovascular Sciences, University of Leicester, Glenfield Hospital, Leicester, LE3 9QP, UK
[181] Research Centre of Applied and Preventive Cardiovascular Medicine, University of Turku, 20520 Turku, Finland
[182] The Department of Clinical Physiology, Turku University Hospital, 20520 Turku, Finland
[183] Clinical Psychology and Psychotherapy, University of Marburg, 35032 Marburg, Germany
[184] Department of Clinical Sciences/Clinical Chemistry, University of Oulu, 90014 Oulu, Finland
[185] Ludwig-Maximilians-Universität, Institute of Medical Informatics, Biometry and Epidemiology, Chair of Epidemiology, 81377 Munich, Germany
[186] South Karelia Central Hospital, 53130 Lappeenranta, Finland
[187] Department of Clinical Sciences/Internal Medicine, University of Oulu, 90014 Oulu, Finland
[188] Institut für Community Medicine, 17489 Greifswald, Germany
[189] Christian-Albrechts-University, University Hospital Schleswig-Holstein, Institute for Clinical Molecular Biology and Department of Internal Medicine I, 24105 Kiel, Germany
[190] Universität zu Lübeck, Medizinische Klinik II, 23562 Lübeck, Germany
[191] Division of Cardiology, Cardiovascular Laboratory, Helsinki University Central Hospital, 00029 Helsinki, Finland

[192] Departments of Medicine and Epidemiology, University of Washington, Seattle, Washington 98195, USA
[193] Department of Psychiatry/EMGO Institute, VU University Medical Center, 1081 BT Amsterdam, The Netherlands
[194] Department of Haematology, University of Cambridge, Cambridge CB2 0PT, UK
[195] NHS Blood and Transplant, Cambridge Centre, Cambridge, CB2 0PT, UK
[196] Leicester NIHR Biomedical Research Unit in Cardiovascular Disease, Glenfield Hospital, Leicester, LE3 9QP, UK
[197] Department of Health Sciences, University of Leicester, University Road, Leicester, LE1 7RH, UK
[198] Department of Medicine, University of Leipzig, 04103 Leipzig, Germany
[199] Coordination Centre for Clinical Trials, University of Leipzig, Härtelstr. 16-18, 04103 Leipzig, Germany
[200] Department of Medicine, Helsinki University Central Hospital, 00290 Helsinki, Finland
[201] Research Program of Molecular Medicine, University of Helsinki, 00014 Helsinki, Finland
[202] Department of Human Genetics, Leiden University Medical Center, 2333 ZC Leiden, the Netherlands
[203] Center of Medical Systems Biology, Leiden University Medical Center, 2333 ZC Leiden, the Netherlands
[204] Department of Medicine, University of Turku and Turku University Hospital, 20520 Turku, Finland
[205] INSERM Cardiovascular Genetics team, CIC 9501, 54000 Nancy, France
[206] Steno Diabetes Center, 2820 Gentofte, Denmark
[207] Center for Human Genomics, Wake Forest University, Winston-Salem, North Carolina 27157, USA
[208] Department of Physiatrics, Lapland Central Hospital, 96101 Rovaniemi, Finland
[209] School of Pathology and Laboratory Medicine, University of Western Australia, Nedlands, Western Australia 6009, Australia
[210] Department of Internal Medicine, University of Oulu, 90014 Oulu, Finland
[211] School of Medicine and Pharmacology, University of Western Australia, Perth, Western Australia 6009, Australia
[212] Department of Clinical Physiology, University of Tampere and Tampere University Hospital, 33520 Tampere, Finland;
[213] Stanford University School of Medicine, Stanford, California 93405, USA
[214] Department of Psychiatry and Human Behavior, University of California, Irvine (UCI), Irvine, California 92617, USA
[215] LIFE Study Centre, University of Leipzig, Leipzig, Germany
[216] Service of Medical Genetics, Centre Hospitalier Universitaire Vaudois (CHUV) University Hospital, 1011 Lausanne, Switzerland
[217] Human Genetics Center and Institute of Molecular Medicine, University of Texas Health Science Center, Houston, Texas 77030, USA
[218] Faculty of Health Science, University of Southern Denmark, 5000 Odense, Denmark
[219] New York University Medical Center, New York, New York 10016, USA
[220] National Institute for Health and Welfare, Department of Mental Health and Substance Abuse Services, Unit for Child and Adolescent Mental Health, 00271 Helsinki, Finland
[221] NIHR Oxford Biomedical Research Centre, Churchill Hospital, Oxford, OX3 7LJ, UK
[222] Department of Urology, Radboud University Nijmegen Medical Centre, 6500 HB Nijmegen, The Netherlands
[223] Institute of Biomedical Sciences, University of Copenhagen, 2200 Copenhagen, Denmark

[224] Faculty of Health Science, University of Aarhus, 8000 Aarhus, Denmark
[225] Department of Psychiatry, Leiden University Medical Centre, 2300 RC Leiden, The Netherlands
[226] Department of Psychiatry, University Medical Centre Groningen, 9713 GZ Groningen, The Netherlands
[227] Department of Neurology, University of Lübeck, Lübeck, Germany
[228] Institute for Paediatric Nutrition Medicine, Vestische Hospital for Children and Adolescents, University of Witten-Herdecke, 45711 Datteln, Germany
[229] Department of Medicine III, Prevention and Care of Diabetes, University of Dresden, 01307 Dresden, Germany
[230] Geriatrics Research and Education Clinical Center, Baltimore Veterans Administration Medical Center, Baltimore, Maryland 21201, USA
[231] Hjelt Institute, Department of Public Health, University of Helsinki, 00014 Helsinki, Finland
[232] South Ostrobothnia Central Hospital, 60220 Seinajoki, Finland
[233] Department of Internal Medicine, Centre Hospitalier Universitaire Vaudois (CHUV) University Hospital, 1011 Lausanne, Switzerland
[234] The Broad Institute of Harvard and MIT, Cambridge, Massachusetts 02142, USA
[235] Department of Psychiatry, Harvard Medical School, Boston, Massachusetts 02115, USA
[236] Pacific Biosciences, Menlo Park, California 94025, USA
[237] Sage Bionetworks, Seattle, Washington 98109, USA
[238] Division of Biostatistics, Washington University School of Medicine, St. Louis, Missouri 63110, USA
[239] Division of Intramural Research, National Heart, Lung and Blood Institute, Framingham Heart Study, Framingham, Massachusetts 01702, USA
[240] Department of Epidemiology and Population Health, Albert Einstein College of Medicine, Bronx, New York 10461, USA
[241] Department of Genetics, University of North Carolina, Chapel Hill, North Carolina 27599, USA
[242] Department of Medical Genetics, University of Helsinki, 00014 Helsinki, Finland
[243] Laboratory of Genetics, National Institute on Aging, Baltimore, Maryland 21224, USA
[244] Division of Community Health Sciences, St George’s, University of London, London, SW17 0RE, UK
[245] Klinikum Grosshadern, 81377 Munich, Germany
[246] Faculty of Medicine, University of Iceland, 101 Reykjavík, Iceland
[247] University of Cambridge Metabolic Research Labs, Institute of Metabolic Science Addenbrooke’s Hospital, CB2 OQQ, Cambridge, UK
[248] Carolina Center for Genome Sciences, School of Public Health, University of North Carolina Chapel Hill, Chapel Hill, North Carolina 27514, USA
[249] Department of Genetics, Harvard Medical School, Boston, Massachusetts 02115, USA
