# Supplementary material for: Mining the Human Phenome Using Allelic Scores That Index Biological Intermediates
Source: PLoS Genet. 2013 Oct 31;9(10):e1003919. doi: 10.1371/journal.pgen.1003919 (PMC3814299; doi:10.1371/journal.pgen.1003919)
Supplement: File S2 — Members of the CRP Consortium. (PDF) [file pgen.1003919.s008.pdf]

Members of the CRP Consortium

Abbas Dehghan, MD PhD[1,2], **Josée Dupuis, PhD**[3,4], **Maja Barbalic, PhD**[5], **Joshua C Bis, PhD**[6], **Gudny Eiriksdottir, MSc**[7], **Chen Lu, M.A.**[3], **Niina Pellikka, BEng**[8], **Henri Wallaschofski, MD**[9], **Johannes Kettunen, MSci**[10], **Peter Henneman, MSc**[11], **Jens Baumert, PhD**[12], **David P Strachan, MD**[13], **Christian Fuchsberger, PhD**[14], **Veronique Vitart, PhD**[15], **James F Wilson, BSc DPhil**[16], **Guillaume Paré, MD MSc**[17], **Silvia Naitza, PhD**[18], **Megan E Rudock, PhD**[19], **Ida Surakka, BSci**[20], **Eco JC de Geus, PhD**[21], **Behrooz Z Alizadeh, PhD**[22], **Jack Guralnik, MD, PhD**[23], **Alan Shuldiner, MD**[24], **Toshiko Tanaka, PhD**[25,26], **Robert YL Zee, PhD**[27], **Renate B Schnabel, MD MSc**[28], **Vijay Nambi, MD**[29], **Maryam Kavousi, MD MSc**[1,2], **Samuli Ripatti, PhD**[20], **Matthias Nauck, MD**[9], **Nicholas L Smith, PhD**[30,31], **Albert V Smith, PhD**[7], **Jouko Sundvall, PhD**[32], **Paul Scheet, PhD**[33], **Yongmei Liu, MD PhD**[19], **Aimo Ruokonen, MD PhD**[34], **Lynda M Rose, MSc**[27], **Martin G Larson, ScD**[4], **Ron C Hoogeveen, PhD**[29], **Nelson B Freimer, MD**[17], **Alexander Teumer, Dipl-Math**[35], **Russell P Tracy, PhD**[36], **Lenore J Launer, PhD**[23], **Julie E Buring, DSc**[27], **Jennifer F Yamamoto, MA**[4], **Aaron R Folsom, MD MPH**[37], **Eric JG Sijbrands, MD PhD**[38], **James Pankow, PhD**[37], **Paul Elliott, MBBS PhD FMedSci**[39], **John F Keaney, MD**[4], **Wei Sun, MD PhD**[40], **Antti-Pekka Sarin, BSci**[20], **João D Fontes, MD**[4], **Sunita Badola, MSc**[41], **Brad C Astor, PhD MPH**[29], **Albert Hofman, MD PhD**[1,2], **Anneli Pouta, MD PhD**[42], **Karl Werdan, MD**[43], **Karin H Greiser, MD**[44,45], **Oliver Kuss, PhD**[44], **Henriette E Meyer zu Schwabedissen, MD**[46], **Joachim Thiery, MD**[47], **Yalda Jamshidi, PhD**[48,49], **Ilja M Nolte, PhD**[22], **Nicole Soranzo, PhD**[50], **Timothy D Spector, MD MSc FRCP**[51], **Henry Völzke, MD**[52], **Alexander N Parker, PhD**[41], **Thor Aspelund, PhD**[7,53], **David Bates, MD MSc**[27], **Lauren Young**[41], **Kim Tsui**[41], **David S Siscovick, MD MPH**[54], **Xiuqing Guo, PhD**[55], **Jerome I Rotter, MD**[55], **Manuela Uda, PhD**[18], **David Schlessinger, PhD**[56], **Igor Rudan, MD**[16,57], **Andrew A Hicks, PhD**[14], **Brenda W Penninx, PhD**[58], **Barbara Thorand, PhD MPH**[12], **Christian Gieger, PhD MS**[12], **Joe Coresh, MD PhD**[29], **Gonneke Willemsen, PhD**[21], **Tamara B Harris, MD MSc**[23], **Andre G Uitterlinden, PhD**[2,38], **Marjo-Riitta Järvelin, MD PhD**[39,42,59], **Kenneth Rice, PhD**[60], **Dörte Radke**[52], **Veikko Salomaa, MD PhD**[61], **Ko Willems van Dijk, PhD**[62], **Eric Boerwinkle, PhD**[5], **Ramachandran S Vasan, MD**[4,63], **Luigi Ferrucci, MD PhD**[25], **Quince D Gibson, MBA**[24], **Stefania Bandinelli, MD**[64], **Harold Snieder, PhD**[22], **Dorret I Boomsma, PhD**[21], **Xiangjun Xiao**[33], **Harry Campbell, MBChB MD**[16], **Caroline Hayward, PhD**[15], **Peter P Pramstaller, MD**[14,65,66], **Cornelia M van Duijn, PhD**[1,2], **Leena Peltonen, MD PhD**[10], **Bruce M Psaty, MD PhD**[54,67], **Vilmundur Gudnason, MD PhD**[7,53], **Paul M Ridker, MD MPH**[27], **Georg Homuth, PhD**[35], **Wolfgang Koenig, MD, PhD**[68], **Christie M Ballantyne, MD**[29], **Jacqueline CM Witteman, PhD**[1,2], **Emelia J Benjamin, MD, ScM**[4,63], **Markus Perola, MD, PhD**[8], and **Daniel I Chasman, PhD**[27]

[1]Department of Epidemiology, Erasmus Medical Center, Rotterdam, The Netherlands [2]Member of Netherlands Consortium for Healthy Aging (NCHA) sponsored by Netherlands Genomics Initiative (NGI), Leiden, The Netherlands [3]Department of Biostatistics, School of Public Health, Boston University, Boston, MA, USA [4]The NHLBI and Boston University's Framingham Heart Study, Framingham, MA, USA [5]Human Genetics Center and Institute of Molecular Medicine, University of Texas Health Science Center at Houston, Houston, TX, USA [6]Department of Medicine, University of Washington, Seattle, WA USA [7]Icelandic Heart Association, Kopavogur, Iceland [8]Unit of Public Health Genomics, Department of Chronic Disease Prevention, National Institute for Health and Welfare, Helsinki, Finland [9]Institute of Clinical Chemistry and Laboratory Medicine, University of Greifswald, Germany [10]Department of Human Genetics, Wellcome Trust Sanger Institute, Wellcome Trust Genome Campus, Hinxton, Cambridge, UK [11]Department of Human Genetics, Leiden University Medical Centre, Leiden, The Netherlands [12]Institute of Epidemiology, Helmholtz Zentrum München, German Research Center for Environmental Health, Neuherberg, Germany [13]Division of Community Health Sciences, St George's University of London, London, UK [14]Institute of Genetic Medicine, European Academy Bozen/Bolzano (EURAC), Bolzano, Italy. Affiliated Institute of University of Lübeck, Lübeck, Germany [15]MRC Human Genetics Unit, Institute of Genetics and Molecular Medicine, Western General Hospital, Edinburgh, UK [16]Centre for Population Health Sciences, University of Edinburgh, Edinburgh EH89AG, UK [17]Center for Cardiovascular Disease Prevention, Harvard Medical School, Boston, MA, USA [18]Istituto di Neurogenetica e Neurofarmacologia, Consiglio Nazionale delle Ricerche, Cagliari, Italy [19]Department of Epidemiology and Prevention, Wake Forest University School of Medicine, Wake Forest, USA [20]Institute for Molecular Medicine Finland FIMM, University of Helsinki, Helsinki, Finland [21]Department of Biological Psychology, VU University, Amsterdam, The Netherlands [22]Unit of Genetic Epidemiology and Bioinformatics, Department of Epidemiology, University Medical Center Groningen, University of Groningen, Groningen, The Netherlands [23]Laboratory of Epidemiology, Demography and Biometry, National Institute on Aging,

NIH, Bethesda, MD, USA [24]Division of Endocrinology, Diabetes and Nutrition, University of Maryland School of Medicine, Maryland, USA [25]Clinical Research Branch, National Institute on Aging, Baltimore, Maryland, USA [26]Medstar Research Institute, Baltimore MD, USA [27]Division of Preventive Medicine, Brigham and Women's Hospital, Boston, USA [28]Department of Medicine, Johannes Gutenberg-University, Mainz, Germany [29]Department of Medicine, Baylor College of Medicine and Center for Cardiovascular Prevention, Methodist DeBakey Heart and Vascular Center, Houston, USA [30]Department of Epidemiology, University of Washington, Seattle, WA, USA [31]Seattle Epidemiologic Research and Information Center of the Department of Veterans Affairs Office of Research and Development, Seattle, WA, USA [32]Unit of Disease Risk, Department of Chronic Disease Prevention, National Institute for Health and Welfare, Helsinki, Finland [33]Department of Epidemiology, MD Anderson Cancer Center, University of Texas, Houston, Texas, USA [34]Department of Clinical Chemistry, University of Oulu, Oulu, Finland [35]Interfaculty Institute for Genetics and Functional Genomics, Ernst-Moritz-Arndt-University Greifswald, 17487 Greifswald, Germany [36]Departments of Pathology and Biochemistry, Colchester Research Facility, Colchester, VT, USA [37]Division of Epidemiology and Community Health, University of Minnesota, Minneapolis, MN, USA [38]Department of Internal Medicine, Erasmus Medical Center, Rotterdam, The Netherlands [39]MRC-HPA Centre for Environment and Health, Department of Epidemiology and Biostatistics, School of Public Health, St Mary's Campus, Imperial College London, London, UK [40]Department of Biostatistics, Department of Genetics, University of North Carolina, Chapel Hill, NC, USA [41]Amgen, Inc, Cambridge, MA, USA [42]Department of Life course and Services, National Institute for Health and Welfare, Helsinki, Finland [43]Department of Medicine III, Martin- Luther-University Halle-Wittenberg, Germany [44]Institute for Medical Epidemiology, Biostatistics, and Informatics, Martin-Luther-University Halle-Wittenberg, Germany [45]Division of Cancer Epidemiology, German Cancer Research Centre, Heidelberg, Germany [46]Department of Pharmacology, Ernst-Moritz-Arndt-University of Greifswald, Germany [47]Institute of Laboratory Medicine, Clinical Chemistry and Molecular Diagnostics (ILM), University of Leipzig, Germany [48]Division of Clinical Developmental Sciences, St George's University of London, London, UK [49]Department of Twin Research and Genetic Epidemiology Unit, St Thomas' Campus, King's College London, St Thomas' Hospital, London, UK [50]Wellcome Trust Sanger Institute, United Kingdom [51]Department of Twin Research and Genetic Epidemiology Unit, King's College London, United Kingdom [52]Institute for Community Medicine, Ernst-Moritz-Arndt-Universität Greifswald, Greifswald, Germany [53]University of Iceland, Reykjavik, Iceland [54]Cardiovascular Health Research Unit, Departments of Medicine, Epidemiology, and Health Services, University of Washington, Seattle, WA, USA [55]Medical Genetics Institute, Cedars-Sinai Medical Center, Los Angeles, CA, USA [56]Laboratory of Genetics, National Institute on Aging, Baltimore, MD 21224, USA [57]Croatian Centre for Global Health, University of Split Medical School, Split, Croatia [58]Department of Psychiatry/EMGO Institute/Neuroscience Campus, VU University Medical Centre, Amsterdam, The Netherlands [59]Institute of Health Sciences and Biocenter Oulu, Faculty of Medicine, University of Oulu, Oulu, Finland [60]Department of Biostatistics, University of Washington, Seattle, WA, USA [61]Unit of Chronic Disease Epidemiology and Prevention, Department of Chronic Disease Prevention , National Institute for Health and Welfare, Helsinki, Finland [62]Departments of Internal Medicine and Human Genetics, Leiden University Medical Centre, Leiden, The Netherlands [63]Preventive Medicine and Cardiology Sections, Department of Medicine, Boston University School of Medicine, Boston, MA, USA [64]Geriatric Unit, Azienda Sanitaria Firenze, Florence, Italy [65]Department of Neurology, General Central Hospital, Bolzano, Italy [66]Department of Neurology, University of Lübeck, Lübeck, Germany [67]Group Health Research Institute, Group Health Cooperative, Seattle, WA, USA [68]Department of Internal Medicine II - Cardiology, University of Ulm Medical Center, Ulm, Germany
