# Supplementary material for: Mining the Human Phenome Using Allelic Scores That Index Biological Intermediates
Source: PLoS Genet. 2013 Oct 31;9(10):e1003919. doi: 10.1371/journal.pgen.1003919 (PMC3814299; doi:10.1371/journal.pgen.1003919)
Supplement: File S3 — Members of the TAG Consortium. (PDF) [file pgen.1003919.s009.pdf]

Members of the TAG Consortium

Helena Furberg[1,2], YunJung Kim[1], Jennifer Dackor[1], Eric Boerwinkle[3], Nora Franceschini[4], Diego Ardissino[5], Luisa Bernardinelli[6,7], Pier M Mannucci[8], Francesco Mauri[9], Piera A Merlini[9], Devin Absher[10], Themistocles L Assimes[11], Stephen P Fortmann[12], Carlos Iribarren[13], Joshua W Knowles[11], Thomas Quertermous[11], Luigi Ferrucci[14], Toshiko Tanaka[15], Joshua C Bis[16,17], Curt D Furberg[18], Talin Haritunians[19], Barbara McKnight[16,20], Bruce M Psaty[16,17,21,22], Kent D Taylor[19], Evan L Thacker[16,23], Peter Almgren[24], Leif Groop[24], Claes Ladenvall[24], Michael Boehnke[25], Anne U Jackson[25], Karen L Mohlke[1,2], Heather M Stringham[25], Jaakko Tuomilehto[26–28], Emelia J Benjamin[29,30], Shih-Jen Hwang[31], Daniel Levy[32], Sarah Rosner Preis[31], Ramachandran S Vasan[29,32], Jubao Duan[33], Pablo V Gejman[33], Douglas F Levinson[34], Alan R Sanders[33], Jianxin Shi[35], Esther H Lips[36], James D McKay[36], Antonio Agudo[37], Luigi Barzan[38], Vladimir Bencko[39], Simone Benhamou[40,41], Xavier Castellsagué[37], Cristina Canova[42], David I Conway[43], Eleonora Fabianova[44], Lenka Foretova[45], Vladimir Janout[46], Claire M Healy[47], Ivana Holcátová[39], Kristina Kjaerheim[48], Pagona Lagiou[49], Jolanta Lissowska[50], Ray Lowry[51], Tatiana V Macfarlane[52], Dana Mates[53], Lorenzo Richiardi[54], Peter Rudnai[55], Neonilia Szeszenia-Dabrowska[56], David Zaridze[57], Ariana Znaor[58], Mark Lathrop[59,60], Paul Brennan[36], Stefania Bandinelli[61], Timothy M Frayling[62], Jack M Guralnik[63], Yuri Milaneschi[64], John R B Perry[62], David Altshuler[65–70], Roberto Elosua[71], Sek Kathiresan[65,68,72], Gavin Lucas[71], Olle Melander[73], Christopher J O'Donnell[74], Veikko Salomaa[75], Stephen M Schwartz[16], Benjamin F Voight[76], Brenda W Penninx[77,78], Johannes H Smit[77,78], Nicole Vogelzangs[77,78], Dorret I Boomsma[79], Eco J C de Geus[79], Jacqueline M Vink[79], Gonneke Willemsen[79], Stephen J Chanock[80], Fangyi Gu[81], Susan E Hankinson[82], David J Hunter[81], Albert Hofman[83], Henning Tiemeier[83,84], Andre G Uitterlinden[83,85], Cornelia M van Duijn[83,86], Stefan Walter[83,87], Daniel I Chasman[88], Brendan M Everett[88,89], Guillaume Paré[88], Paul M Ridker[88,89], Ming D Li[90], Hermine H Maes[91,92], Janet Audrain-McGovern[93], Danielle Posthuma[94,95], Laura M Thornton[96], Caryn Lerman[93,97], Jaakko Kaprio[26,75,98], Jed E Rose[99], John P A Ioannidis[100–102], Peter Kraft[81], Dan-Yu Lin[103] & Patrick F Sullivan[1,2]

[1] Department of Genetics, University of North Carolina, Chapel Hill, North Carolina, USA. [2]University of North Carolina Lineberger Comprehensive Cancer Center, University of North Carolina, Chapel Hill, North Carolina, USA. [3]Human Genetics Center and Institute for Molecular Medicine, University of Texas Health Science Center, Houston, Texas, USA. [4]Department of Epidemiology, University of North Carolina, Chapel Hill, North Carolina, USA. [5]Division of Cardiology, Azienda Ospedaliero-Universitaria di Parma, Parma, Italy. [6]Statistical Laboratory, Centre for Mathematical Sciences, University of Cambridge, Cambridge, UK. [7]Department of Applied Health Sciences, University of Pavia, Pavia, Italy. [8]Department of Internal Medicine and Medical Specialties, Fondazione Istituto di Ricovero e Cura a Carattere Scientifico, Ospedale Maggiore, Mangiagalli e Regina Elena, University of Milan, Milan, Italy. [9]Department of Cardiology, Azienda Ospedaliera Niguarda Ca' Granda, Milan, Italy. [10]HudsonAlpha Institute for Biotechnology, Huntsville, Alabama, USA. [11]Cardiovascular Medicine, Stanford University, Stanford, California, USA. [12]Stanford Prevention Research Center, Stanford University, Stanford, California, USA. [13]Kaiser Permanente Northern California Division of Research, Oakland, California, USA. [14]National Institute on Aging, Baltimore, Maryland, USA. [15]Medstart Research Institute, National Institute on Aging, Baltimore, Maryland, USA. [16]Cardiovascular Health Research Unit, University of Washington, Seattle, Washington, USA. [17]Department of Medicine, University of Washington, Seattle, Washington, USA. [18]Division of Public Health Sciences, Wake Forest University Health Sciences, Winston-Salem, North Carolina, USA. [19]Medical Genetics Institute, Cedars-Sinai Medical Center, Los Angeles, California, USA. [20]Department of Biostatistics, University of Washington, Seattle, Washington, USA. [21]Department of Epidemiology and Health Services, University of Washington, Seattle, Washington, USA. [22]Group Health Research Institute, Seattle, Washington, USA. [23]Department of Epidemiology, University of Washington, Seattle, Washington, USA. [24]Department of Clinical Sciences, Diabetes and Endocrinology Unit, Lund University, Malmö, Sweden. [25]Department of Biostatistics, School of Public Health, University of Michigan, Ann Arbor, Michigan, USA. [26]Hjelt Institute, Department of Public Health, University of Helsinki, Helsinki, Finland. [27]Diabetes Prevention Unit, National Institute for Health and Welfare, Helsinki, Finland. [28]Finland South Ostrobothnia Central Hospital, Seinäjoki, Finland. [29]Boston University School of Medicine, Boston, Massachusetts, USA. [30]Boston University School of Public Health, Boston, Massachusetts, USA. [31]Center for Population Studies, National Heart, Lung, and Blood Institute, Bethesda, Maryland, USA. [32]Department of Medicine, Sections of Preventive Medicine and Cardiology, Boston University School of Medicine, Boston, Massachusetts, USA. [33]Center for Psychiatric Genetics, NorthShore University HealthSystem Research Institute, Evanston, Illinois, USA. [34]Department of Psychiatry and Behavioral Sciences, Stanford University, Stanford, California, USA. [35]Biostatistics Branch, Division of Cancer Epidemiology and Genetics, National Cancer Institute, Bethesda, Maryland, USA. [36]International Agency for Research on Cancer (IARC), Lyon, France. [37]Institut Català d'Oncologia, Barcelona, Spain. [38]General Hospital, Pordenone, Italy. [39]Institute of Hygiene and Epidemiology, First Faculty of Medicine, Charles University, Prague, Czech

Republic. [40]Institut National de la santé et de la Recherche Medicalé (INSERM) U794, Paris, France. [41]Institut Gustave Roussy, Villejuif, France. [42]Department of Environmental Medicine and Public Health, University of Padua, Padua, Italy. [43]University of Glasgow Medical Faculty Dental School, Glasgow, UK. [44]Specialized Institute of Hygiene and Epidemiology, Banska Bystrica, Slovakia. [45]Department of Cancer Epidemiology and Genetics, Masaryk Memorial Cancer Institute, Brno, Czech Republic. [46]Palacky University, Olomouc, Czech Republic. [47]Trinity College School of Dental Science, Dublin, Ireland. [48]Cancer Registry of Norway, Oslo, Norway. [49]University of Athens School of Medicine, Athens, Greece. [50]Department of Cancer Epidemiology and Prevention, Maria Sklodowska-Curie Cancer Center and Institute of Oncology, Warsaw, Poland. [51]University of Newcastle Dental School, Newcastle, UK. [52]University of Aberdeen School of Medicine, Aberdeen, UK. [53]Institute of Public Health, Bucharest, Romania. [54]Center for Experimental Research and Medical Studies, University of Turin, Turin, Italy. [55]National Institute of Environmental Health, Budapest, Hungary. [56]Department of Epidemiology, Institute of Occupational Medicine, Lodz, Poland. [57]Institute of Carcinogenesis, Cancer Research Centre, Moscow, Russia. [58]Croatian National Cancer Registry, Zagreb, Croatia. [59]Centre National de Genotypage, Institut Genomique, Comissariat à l'énergie Atomique, Evry, France. [60]Fondation Jean Dausset-Centre d'Étude du Polymorphisme Humain (CEPH), Paris, France. [61]Geriatric Unit, Azienda Sanitaria di Firenze, Florence, Italy. [62]Genetics of Complex Traits, Peninsula Medical School, The University of Exeter, Exeter, UK. [63]Laboratory of Epidemiology, Demography and Biometry, National Institute on Aging, Bethesda, Maryland, USA. [64]Tuscany Health Regional Agency, Florence, Italy. [65]Broad Institute of Harvard and Massachusetts Institute of Technology, Cambridge, Massachusetts, USA. [66]Department of Molecular Biology, Massachusetts General Hospital, Boston, Massachusetts, USA. [67]Diabetes Unit, Massachusetts General Hospital, Boston, Massachusetts, USA. [68]Center for Human Genetics Research, Massachusetts General Hospital, Boston, Massachusetts, USA. [69]Department of Genetics, Harvard Medical School, Boston, Massachusetts, USA. [70]Department of Medicine, Harvard Medical School, Boston, Massachusetts, USA. [71]Cardiovascular Epidemiology and Genetics, Institut Municipal d'Investigacio Medica, Barcelona, Spain. [72]Harvard Medical School, Boston, Massachusetts, USA. [73]Department of Clinical Sciences, Hypertension and Cardiovascular Diseases, University Hospital Malmö, Lund University, Malmö, Sweden. [74]National Heart, Lung, and Blood Institute's Framingham Heart Study, Framingham, Massachusetts, USA. [75]National Institute for Health and Welfare (THL), Helsinki, Finland. [76]Program in Medical and Population Genetics, Broad Institute of Harvard and Massachusetts Institute of Technology, Cambridge, Massachusetts, USA. [77]EMGO Institute, Vrije Universiteit (VU) Medical Center, Amsterdam, The Netherlands. [78]Department of Psychiatry, VU University Medical Center, Amsterdam, The Netherlands. [79]Biological Psychology, VU University Amsterdam, Amsterdam, The Netherlands. [80]Division of Cancer Epidemiology and Genetics, National Cancer Institute, Bethesda, Maryland, USA. [81]Program in Molecular and Genetic Epidemiology, Department of Epidemiology, Harvard University, Boston, Massachusetts, USA. [82]Channing Laboratory, Department of Medicine, Brigham and Women's Hospital and Harvard Medical School, Boston, Massachusetts, USA. [83]Department of Epidemiology, Erasmus Medical Center, Member of the Netherlands Consortium on Healthy Aging, Rotterdam, The Netherlands. [84]Department of Child and Adolescent Psychiatry, Erasmus Medical Center, Rotterdam, The Netherlands. [85]Department of Internal Medicine, Erasmus Medical Center, Rotterdam, The Netherlands. [86]Centre for Medical Systems Biology, Erasmus Medical Center, Rotterdam, The Netherlands. [87]Department of Public Health, Erasmus Medical Center, Rotterdam, The Netherlands. [88]Division of Preventive Medicine, Department of Medicine, Brigham and Women's Hospital, Harvard Medical School, Boston, Massachusetts, USA. [89]Division of Cardiovascular Medicine, Department of Medicine, Brigham and Women's Hospital, Harvard Medical School, Boston, Massachusetts, USA. [90]Department of Psychiatry and Neurobehavioural Sciences, University of Virginia, Charlottesville, Virginia, USA. [91]Virginia Institute for Psychiatric and Behavioral Genetics, Virginia Commonwealth University, Richmond, Virginia, USA. [92]Massey Cancer Center, Virginia Commonwealth University, Richmond, Virginia, USA. [93]Department of Psychiatry, University of Pennsylvania, Philadelphia, Pennsylvania, USA. [94]Department of Functional Genomics, VU Amsterdam, Amsterdam, The Netherlands. [95]Department of Medical Genomics, VU University Medical Center Amsterdam, Amsterdam, The Netherlands. [96]Department of Psychiatry, University of North Carolina, Chapel Hill, North Carolina, USA. [97]Abramson Cancer Center, University of Pennsylvania, Philadelphia, Pennsylvania, USA. [98]Institute for Molecular Medicine, University of Helsinki, Helsinki, Finland. [99]Department of Psychiatry and Behavioral Sciences, Duke University Medical Center, Durham, North Carolina, USA. [100]Department of Hygiene and Epidemiology, University of Ioannina School of Medicine, Ioannina, Greece. [101]Tufts Clinical and Translational Science Institute, Tufts University School of Medicine, Boston, Massachusetts, USA. [102]Center for Genetic Epidemiology and Modeling, Institute for Clinical Research and Health Policy Studies, Tufts Medical Center, Boston, Massachusetts, USA. [103]Department of Biostatistics, University of North Carolina, Chapel Hill, North Carolina, USA.
