# Supplementary material for: Mining the Human Phenome Using Allelic Scores That Index Biological Intermediates
Source: PLoS Genet. 2013 Oct 31;9(10):e1003919. doi: 10.1371/journal.pgen.1003919 (PMC3814299; doi:10.1371/journal.pgen.1003919)
Supplement: Table S1 — Association between case-control status in the WTCCC and an allelic score that proxies for BMI. (PDF) [file pgen.1003919.s010.pdf]

Table S1. Association between case-control status in the WTCCC and an allelic score that proxies for BMI. The left hand side of the table shows results for an allelic score consisting of all SNPs that meet a certain threshold ("All SNPs"), whilst the right side shows results for an allelic score consisting of all SNPs that meet a certain threshold with SNPs from known regions removed from its construction ("Complement"). SNPs have either been weighted according to their effect size from a previous meta-analysis ("Weighted"), or with each SNP getting an equal weighting ("Unweighted"). Results are shown for seventeen different p value thresholds and the number of SNPs that went into construction of the score for each threshold is listed also. Results are shown for seven different diseases. BD = Bipolar Disorder; CHD = Coronary Heart Disease; HT = Hypertension; CD = Crohn's Disease; RA = Rheumatoid Arthritis; T1D = Type 1 Diabetes; T2D = Type 2 Diabetes; Dir = Direction of effect; Pval = P value.

All SNPs					Complement				
	Weighted		Unweighted			Weighted		Unweighted	
	Dir	Pval	Dir	Pval		Dir	Pval	Dir	Pval
$p<5x10^{-8}$ (158 SNPs)					$p<5x10^{-8}$ (8 SNPs)				
BD	-	0.62	-	0.72		-	0.91	-	0.79
CHD	+	0.17	+	0.22		+	0.91	-	0.90
HT	-	0.58	-	0.74		+	0.97	-	0.99
CD	+	0.90	+	0.75		+	0.61	+	0.58
RA	+	0.15	+	0.086		+	0.40	+	0.40
T1D	+	0.77	+	0.79		-	0.66	-	0.51
T2D	+	$4.3x10^{-7}$	+	$1.5x10^{-5}$		-	0.86	-	0.95
$p<5x10^{-7}$ (224 SNPs)					$p<5x10^{-7}$ (23 SNPs)				
BD	-	0.88	+	0.83		+	0.45	+	0.46
CHD	+	0.13	+	0.15		+	0.15	+	0.16
HT	-	0.63	-	0.77		+	0.12	+	0.11
CD	+	0.81	+	0.57		+	0.17	+	0.13
RA	+	0.15	+	0.11		+	0.73	+	0.75
T1D	+	0.75	+	0.69		-	0.89	-	0.82
T2D	+	$1.3x10^{-7}$	+	$3.0x10^{-6}$		+	0.17	+	0.13

$p<5x10^{-6}$ (398 SNPs)					$p<5x10^{-6}$ (140 SNPs)				
BD	-	0.30	-	0.26		-	$7.1x10^{-3}$	-	$7.7x10^{-3}$
CHD	+	0.18	+	0.26		-	0.74	-	0.83
HT	-	0.53	-	0.63		-	0.87	+	0.99
CD	-	0.62	-	0.63		-	0.23	-	0.34
RA	+	0.18	+	0.20		+	0.94	-	0.92
T1D	+	0.86	+	0.94		-	0.67	-	0.52
T2D	+	$5.3x10^{-8}$	+	$2.7x10^{-6}$		+	0.30	+	0.23
$p<5x10^{-5}$ (861 SNPs)					$p<5x10^{-5}$ (534 SNPs)				
BD	-	0.20	-	0.19		-	0.021	-	0.031
CHD	+	0.24	+	0.33		-	0.76	-	0.94
HT	-	0.76	-	0.99		+	0.72	+	0.64
CD	-	0.50	-	0.51		-	0.32	-	0.41
RA	+	0.37	+	0.65		-	0.62	-	0.49
T1D	+	0.84	+	0.99		-	0.88	-	0.72
T2D	+	$3.4x10^{-8}$	+	$6.8x10^{-6}$		+	0.14	+	0.13
$p<5x10^{-4}$ (2366 SNPs)					$p<5x10^{-4}$ (1895 SNPs)				
BD	-	0.063	-	0.062		-	$7.1x10^{-3}$	-	0.013
CHD	+	0.45	+	0.64		-	0.57	-	0.73
HT	+	0.83	+	0.56		+	0.46	+	0.39
CD	-	0.67	-	0.79		-	0.56	-	0.70
RA	+	0.40	+	0.61		-	0.87	-	0.85
T1D	-	0.85	-	0.71		-	0.41	-	0.34

T2D	+	$3.9x10^{-9}$	+	$9.8x10^{-7}$		+	$4.8x10^{-3}$	+	$3.3x10^{-3}$
$p<5x10^{-3}$ (8402 SNPs)					$p<5x10^{-3}$ (7577 SNPs)				
BD	-	0.036	-	0.062		-	$9.0x10^{-3}$	-	0.025
CHD	+	0.36	+	0.41		+	0.89	+	0.65
HT	+	0.20	+	0.082		+	0.077	+	0.050
CD	+	0.46	+	0.27		+	0.49	+	0.33
RA	+	0.50	+	0.66		-	0.94	-	0.98
T1D	-	0.90	-	0.84		-	0.57	-	0.56
T2D	+	$1.3x10^{-13}$	+	$1.8x10^{-11}$		+	$1.6x10^{-7}$	+	$5.4x10^{-8}$
$p<5x10^{-2}$ (36921 SNPs)					$p<5x10^{-2}$ (35246 SNPs)				
BD	-	0.013	-	0.028		-	$3.2x10^{-3}$	-	$8.7x10^{-3}$
CHD	+	0.20	+	0.25		+	0.48	+	0.42
HT	+	0.58	+	0.46		+	0.48	+	0.42
CD	+	0.30	+	0.17		+	0.32	+	0.21
RA	+	0.78	-	0.98		-	0.77	-	0.71
T1D	+	0.98	-	0.93		-	0.69	-	0.64
T2D	+	$<2x10^{-16}$	+	1.3x10-^{14}		+	$6.3x10^{-12}$	+	$5.1x10^{-12}$
$p<0.1$ (60232 SNPs)					$p<0.1$ (58020 SNPs)				
BD	-	$8.6x10^{-3}$	-	0.015		-	$2.3x10^{-3}$	-	$5.3x10^{-3}$
CHD	+	0.28	+	0.42		+	0.60	+	0.62
HT	+	0.90	+	0.87		+	0.84	+	0.88
CD	+	0.34	+	0.28		+	0.37	+	0.32
RA	-	0.89	-	0.56		-	0.47	-	0.35

T1D	-	0.87	-	0.70		-	0.60	-	0.50
T2D	+	$3.3x10^{-16}$	+	$3.2x10^{-13}$		+	$2.9x10^{-11}$	+	$6.9x10^{-11}$
p<0.2 (101903 SNPs)					p<0.2 (98818 SNPs)				
BD	-	0.025	-	0.058		-	$9.1x10^{-3}$	-	0.028
CHD	+	0.34	+	0.57		+	0.64	+	0.74
HT	-	0.98	-	0.94		-	0.96	-	0.86
CD	+	0.58	+	0.65		+	0.63	+	0.70
RA	-	0.58	-	0.20		-	0.28	-	0.12
T1D	-	0.97	-	0.75		-	0.72	-	0.61
T2D	+	$<2x10^{-16}$	+	$1.5x10^{-13}$		+	$8.1x10^{-12}$	+	$3.0x10^{-11}$
p<0.3 (140135 SNPs)					p<0.3 (136309 SNPs)				
BD	-	0.030	-	0.073		-	0.011	-	0.037
CHD	+	0.34	+	0.58		+	0.61	+	0.73
HT	-	0.99	-	0.88		-	0.97	-	0.81
CD	+	0.59	+	0.71		+	0.63	+	0.74
RA	-	0.37	-	0.074		-	0.18	-	0.050
T1D	+	0.96	-	0.83		-	0.79	-	0.68
T2D	+	$<2x10^{-16}$	+	$1.0x10^{-13}$		+	$1.7x10^{-12}$	+	$9.1x10^{-12}$
p<0.4 (176024 SNPs)					p<0.4 (171538 SNPs)				
BD	-	0.046	-	0.15		-	0.021	-	0.093
CHD	+	0.33	+	0.55		+	0.57	+	0.64
HT	+	0.95	+	0.95		+	0.96	+	0.99
CD	+	0.73	+	0.95		+	0.74	+	0.94

RA	-	0.31	-	0.058		-	0.16	-	0.048
T1D	+	0.88	-	0.99		-	0.89	-	0.90
T2D	+	$<2x10^{-16}$	+	$1.9x10^{-13}$		+	$8.2x10^{-13}$	+	$8.6x10^{-12}$
p<0.5 (210612 SNPs)					p<0.5 (205518 SNPs)				
BD	-	0.050	-	0.16		-	0.023	-	0.11
CHD	+	0.31	+	0.50		+	0.54	+	0.59
HT	-	0.87	-	0.78		-	0.86	-	0.75
CD	+	0.79	-	0.92		+	0.80	-	0.95
RA	-	0.22	-	0.023		-	0.11	-	0.021
T1D	+	0.85	+	0.95		-	0.93	-	0.98
T2D	+	$<2x10^{-16}$	+	$3.6x10^{-13}$		+	$6.8x10^{-13}$	+	$1.3x10^{-11}$
p<0.6 (244122 SNPs)					p<0.6 (238451 SNPs)				
BD	-	0.052	-	0.19		-	0.026	-	0.14
CHD	+	0.31	+	0.51		+	0.51	+	0.56
HT	-	0.82	-	0.64		-	0.81	-	0.62
CD	+	0.86	-	0.78		+	0.85	-	0.84
RA	-	0.20	-	0.019		-	0.095	-	0.018
T1D	+	0.81	+	0.88		-	0.99	+	0.91
T2D	+	$<2x10^{-16}$	+	$9.3x10^{-13}$		+	$7.5x10^{-13}$	+	$2.6x10^{-11}$
p<0.7 (277294 SNPs)					p<0.7 (2710176 SNPs)				
BD	-	0.057	-	0.23		-	0.029	-	0.18
CHD	+	0.31	+	0.53		+	0.50	+	0.54
HT	-	0.79	-	0.57		-	0.79	-	0.56

CD	+	0.89	-	0.75		+	0.87	-	0.83
RA	-	0.19	-	0.020		-	0.093	-	0.019
T1D	+	0.88	-	0.91		-	0.93	-	0.91
T2D	+	$<2x10^{-16}$	+	$4.0x10^{-12}$		+	$9.0x10^{-13}$	+	$8.3x10^{-11}$
p<0.8 (309981 SNPs)					p<0.8 (303188 SNPs)				
BD	-	0.051	-	0.19		-	0.026	-	0.15
CHD	+	0.34	+	0.75		+	0.54	+	0.71
HT	-	0.78	-	0.55		-	0.78	-	0.54
CD	+	0.98	-	0.43		+	0.96	-	0.51
RA	-	0.18	-	0.016		-	0.086	-	0.016
T1D	+	0.95	-	0.67		-	0.87	-	0.68
T2D	+	$<2x10^{-16}$	+	$2.6x10^{-11}$		+	$1.2x10^{-12}$	+	$4.2x10^{-10}$
p<0.9 (342383 SNPs)					p<0.9 (335045 SNPs)				
BD	-	0.052	-	0.22		-	0.027	-	0.18
CHD	+	0.37	-	0.99		+	0.56	+	0.94
HT	-	0.76	-	0.46		-	0.76	-	0.46
CD	-	0.98	-	0.29		+	0.99	-	0.36
RA	-	0.18	-	0.021		-	0.087	-	0.020
T1D	+	0.97	-	0.57		-	0.85	-	0.59
T2D	+	$<2x10^{-16}$	+	$2.7x10^{-10}$		+	$1.6x10^{-12}$	+	$3.3x10^{-9}$
All (375110 SNPs)					All (367220 SNPs)				
BD	-	0.051	-	0.14		-	0.026	-	0.11
CHD	+	0.37	-	0.82		+	0.57	-	0.91

HT	-	0.76	-	0.39		-	0.76	-	0.39
CD	-	0.97	-	0.21		-	0.99	-	0.26
RA	-	0.18	-	$9.0x10^{-3}$		-	0.085	-	$9.1x10^{-3}$
T1D	+	0.97	-	0.44		-	0.85	-	0.47
T2D	+	$<2x10^{-16}$	+	$4.9x10^{-9}$		+	$1.8x10^{-12}$	+	$5.0x10^{-8}$
