# Supplementary material for: Mining the Human Phenome Using Allelic Scores That Index Biological Intermediates
Source: PLoS Genet. 2013 Oct 31;9(10):e1003919. doi: 10.1371/journal.pgen.1003919 (PMC3814299; doi:10.1371/journal.pgen.1003919)
Supplement: Table S2 — Association between case-control status in the WTCCC and an allelic score that proxies for CRP. (PDF) [file pgen.1003919.s011.pdf]

Table S2. Association between case-control status in the WTCCC and an allelic score that proxies for CRP. The left hand side of the table shows results for an allelic score consisting of all SNPs that meet a certain threshold ("All SNPs"), whilst the right side shows results for an allelic score consisting of all SNPs that meet a certain threshold with SNPs from known regions removed from its construction ("Complement"). SNPs have either been weighted according to their effect size from a previous meta-analysis ("Weighted"), or with each SNP getting an equal weighting ("Unweighted"). Results are shown for seventeen different p value thresholds and the number of SNPs that went into construction of the score for each threshold is listed also. Results are shown for seven different diseases. BD = Bipolar Disorder; CHD = Coronary Heart Disease; HT = Hypertension; CD = Crohn's Disease; RA = Rheumatoid Arthritis; T1D = Type 1 Diabetes; T2D = Type 2 Diabetes; Dir = Direction of effect; Pval = P value.

All SNPs					Complement				
	Weighted		Unweighted			Weighted		Unweighted	
	Dir	Pval	Dir	Pval		Dir	Pval	Dir	Pval
$p<5x10^{-8}$ (152 SNPs)					$p<5x10^{-8}$ (0 SNPs)				
BD	+	0.11	+	0.13		+	NA	NA	NA
CHD	+	0.80	+	0.75		+	NA	NA	NA
HT	+	0.23	+	0.18		+	NA	NA	NA
CD	+	0.051	+	0.035		+	NA	NA	NA
RA	+	0.028	+	$4.0x10^{-3}$		+	NA	NA	NA
T1D	+	0.15	+	0.11		+	NA	NA	NA
T2D	+	0.50	+	0.47		+	NA	NA	NA
$p<5x10^{-7}$ (184 SNPs)					$p<5x10^{-7}$ (0 SNPs)				
BD	+	0.086	+	0.087		+	NA	NA	NA
CHD	+	0.63	+	0.44		+	NA	NA	NA
HT	+	0.15	+	0.083		+	NA	NA	NA
CD	+	0.031	+	0.016		+	NA	NA	NA
RA	+	0.023	+	$3.1x10^{-3}$		+	NA	NA	NA
T1D	+	0.080	+	0.034		+	NA	NA	NA
T2D	+	0.32	+	0.23		+	NA	NA	NA

$p<5x10^{-6}$<br>(262 SNPs)					$p<5x10^{-6}$<br>(36 SNPs)				
BD	+	0.12	+	0.17		-	0.40	-	0.41
CHD	+	0.43	+	0.22		+	0.40	+	0.32
HT	+	0.17	+	0.11		-	0.51	-	0.58
CD	+	0.048	+	0.046		-	0.24	-	0.21
RA	+	0.011	+	$1.2x10^{-3}$		+	0.64	+	0.55
T1D	+	0.068	+	0.036		+	0.58	+	0.56
T2D	+	0.19	+	0.084		+	0.029	+	0.016
$p<5x10^{-5}$<br>(388 SNPs)					$p<5x10^{-5}$<br>(112 SNPs)				
BD	+	0.15	+	0.24		-	0.46	-	0.48
CHD	+	0.36	+	0.18		+	0.33	+	0.27
HT	+	0.21	+	0.18		-	0.43	-	0.54
CD	+	0.016	+	$7.0x10^{-3}$		+	0.97	+	0.83
RA	+	$3.0x10^{-3}$	+	$1.4x10^{-4}$		+	0.10	+	0.042
T1D	+	0.077	+	0.063		+	0.60	+	0.61
T2D	+	0.055	+	$6.9x10^{-3}$		+	$1.2x10^{-3}$	+	$2.6x10^{-4}$
$p<5x10^{-4}$<br>(911 SNPs)					$p<5x10^{-4}$<br>(518 SNPs)				
BD	+	0.083	+	0.12		+	0.91	+	0.78
CHD	+	0.15	+	0.057		+	0.14	+	0.12
HT	+	0.085	+	0.055		+	0.46	+	0.29
CD	+	$4.3x10^{-3}$	+	$3.3x10^{-3}$		+	0.41	+	0.39
RA	+	$6.5x10^{-4}$	+	$1.2x10^{-4}$		+	0.021	+	0.016
T1D	+	0.056	+	0.077		+	0.15	+	0.19

T2D	+	$9.5x10^{-3}$	+	$1.0x10^{-3}$		+	$2.2x10^{-4}$	+	$2.0x10^{-4}$
$p<5x10^{-3}$ (3798 SNPs)					$p<5x10^{-3}$ (3163 SNPs)				
BD	+	0.093	+	0.15		+	0.84	+	0.72
CHD	+	0.033	+	0.020		+	0.051	+	0.049
HT	+	0.073	+	0.076		+	0.46	+	0.35
CD	+	$2.2x10^{-3}$	+	$4.7x10^{-3}$		+	0.25	+	0.24
RA	+	$7.6x10^{-4}$	+	$2.4x10^{-3}$		+	0.050	+	0.085
T1D	+	0.038	+	0.089		+	0.087	+	0.14
T2D	+	$1.4x10^{-3}$	+	$6.6x10^{-4}$		+	$1.1x10^{-3}$	+	$1.8x10^{-3}$
$p<5x10^{-2}$ (24159 SNPs)					$p<5x10^{-2}$ (22966 SNPs)				
BD	+	0.15	+	0.31		+	0.90	+	0.98
CHD	+	0.021	+	0.048		+	0.073	+	0.17
HT	+	0.12	+	0.26		+	0.56	+	0.71
CD	+	$8.3x10^{-4}$	+	$4.7x10^{-3}$		+	0.080	+	0.13
RA	+	$8.4x10^{-3}$	+	0.069		+	0.21	+	0.44
T1D	+	0.046	+	0.13		+	0.10	+	0.18
T2D	+	$4.1x10^{-5}$	+	$4.8x10^{-5}$		+	$1.1x10^{-4}$	+	$2.4x10^{-4}$
$p<0.1$ (44677 SNPs)					$p<0.1$ (43193 SNPs)				
BD	+	0.12	+	0.24		+	0.66	+	0.69
CHD	+	$8.9x10^{-3}$	+	0.021		+	0.033	+	0.079
HT	+	0.13	+	0.27		+	0.53	+	0.67
CD	+	$3.0x10^{-4}$	+	$1.6x10^{-3}$		+	0.028	+	0.041
RA	+	0.014	+	0.076		+	0.22	+	0.36

T1D	+	0.023	+	0.046		+	0.045	+	0.058
T2D	+	$2.6x10^{-6}$	+	$2.9x10^{-6}$		+	$6.3x10^{-6}$	+	$1.3x10^{-5}$
p<0.2 (83740 SNPs)					p<0.2 (81768 SNPs)				
BD	+	0.14	+	0.29		+	0.59	+	0.62
CHD	+	$7.3x10^{-3}$	+	0.023		+	0.023	+	0.057
HT	+	0.18	+	0.45		+	0.57	+	0.82
CD	+	$2.3x10^{-4}$	+	$1.7x10^{-3}$		+	0.015	+	0.025
RA	+	0.083	+	0.39		+	0.54	+	0.84
T1D	+	0.012	+	0.018		+	0.021	+	0.020
T2D	+	$1.0x10^{-6}$	+	$4.4x10^{-6}$		+	$2.6x10^{-6}$	+	$1.4x10^{-5}$
p<0.3 (121099 SNPs)					p<0.3 (118734 SNPs)				
BD	+	0.25	+	0.58		+	0.81	+	0.98
CHD	+	0.018	+	0.075		+	0.054	+	0.16
HT	+	0.24	+	0.58		+	0.65	+	0.93
CD	+	$6.9x10^{-4}$	+	$8.6x10^{-3}$		+	0.031	+	0.077
RA	+	0.14	+	0.62		+	0.68	-	0.90
T1D	+	0.017	+	0.036		+	0.030	+	0.042
T2D	+	$8.6x10^{-7}$	+	$6.0x10^{-6}$		+	$2.5x10^{-6}$	+	$1.9x10^{-5}$
p<0.4 (157932 SNPs)					p<0.4 (155241 SNPs)				
BD	+	0.31	+	0.68		+	0.90	-	0.92
CHD	+	0.028	+	0.14		+	0.083	+	0.28
HT	+	0.25	+	0.60		+	0.65	+	0.95
CD	+	$6.0x10^{-4}$	+	$6.9x10^{-3}$		+	0.025	+	0.056

RA	+	0.17	+	0.65		+	0.72	-	0.92
T1D	+	0.021	+	0.059		+	0.036	+	0.068
T2D	+	$6.0x10^{-7}$	+	$5.5x10^{-6}$		+	$1.8x10^{-6}$	+	$1.6x10^{-5}$
p<0.5 (194853 SNPs)					p<0.5 (191822 SNPs)				
BD	+	0.36	+	0.78		+	0.97	-	0.83
CHD	+	0.028	+	0.15		+	0.084	+	0.29
HT	+	0.23	+	0.53		+	0.61	+	0.84
CD	+	$5.0x10^{-4}$	+	$5.2x10^{-3}$		+	0.020	+	0.040
RA	+	0.17	+	0.59		+	0.71	+	0.99
T1D	+	0.017	+	0.043		+	0.030	+	0.050
T2D	+	$2.1x10^{-7}$	+	$7.9x10^{-7}$		+	$6.1x10^{-7}$	+	$2.3x10^{-6}$
p<0.6 (231075 SNPs)					p<0.6 (227678 SNPs)				
BD	+	0.36	+	0.77		+	0.97	-	0.88
CHD	+	0.026	+	0.14		+	0.077	+	0.27
HT	+	0.22	+	0.50		+	0.59	+	0.77
CD	+	$4.8x10^{-4}$	+	$4.6x10^{-3}$		+	0.018	+	0.032
RA	+	0.18	+	0.64		+	0.73	-	0.99
T1D	+	0.019	+	0.049		+	0.032	+	0.056
T2D	+	$1.4x10^{-7}$	+	$4.2x10^{-7}$		+	$3.9x10^{-7}$	+	$1.1x10^{-6}$
p<0.7 (267164 SNPs)					p<0.7 (263431 SNPs)				
BD	+	0.36	+	0.75		+	0.96	-	0.92
CHD	+	0.028	+	0.17		+	0.080	+	0.31
HT	+	0.20	+	0.37		+	0.53	+	0.59

CD	+	$4.0x10^{-4}$	+	$3.0x10^{-3}$		+	0.015	+	0.019
RA	+	0.17	+	0.56		+	0.69	+	0.88
T1D	+	0.018	+	0.045		+	0.031	+	0.052
T2D	+	$8.5x10^{-8}$	+	$1.5x10^{-7}$		+	$2.3x10^{-7}$	+	$3.5x10^{-7}$
p<0.8<br>(303012 SNPs)					p<0.8<br>(298911 SNPs)				
BD	+	0.38	+	0.78		+	0.98	-	0.91
CHD	+	0.028	+	0.17		+	0.082	+	0.31
HT	+	0.20	+	0.37		+	0.54	+	0.58
CD	+	$3.4x10^{-4}$	+	$1.9x10^{-3}$		+	0.013	+	0.013
RA	+	0.17	+	0.51		+	0.68	+	0.83
T1D	+	0.019	+	0.054		+	0.033	+	0.063
T2D	+	$8.4x10^{-8}$	+	$2.0x10^{-7}$		+	$2.3x10^{-7}$	+	$4.5x10^{-7}$
p<0.9<br>(339088 SNPs)					p<0.9<br>(334683 SNPs)				
BD	+	0.37	+	0.72		+	0.97	-	0.97
CHD	+	0.028	+	0.17		+	0.081	+	0.30
HT	+	0.20	+	0.39		+	0.53	+	0.60
CD	+	$3.0x10^{-4}$	+	$1.1x10^{-3}$		+	0.012	+	$7.1x10^{-3}$
RA	+	0.18	+	0.65		+	0.70	+	0.99
T1D	+	0.020	+	0.075		+	0.034	+	0.086
T2D	+	$8.2x10^{-8}$	+	$3.2x10^{-7}$		+	$2.3x10^{-7}$	+	$7.3x10^{-7}$
All<br>(375099 SNPs)					All<br>(370379 SNPs)				
BD	+	0.37	+	0.64		+	0.96	+	0.92
CHD	+	0.028	+	0.11		+	0.079	+	0.20

HT	+	0.20	+	0.29		+	0.53	+	0.46
CD	+	$2.9x10^{-4}$	+	$5.6x10^{-4}$		+	0.011	+	$3.9x10^{-3}$
RA	+	0.17	+	0.59		+	0.69	+	0.91
T1D	+	0.020	+	0.068		+	0.033	+	0.076
T2D	+	$7.6x10^{-8}$	+	$1.4x10^{-7}$		+	$2.1x10^{-7}$	+	$3.3x10^{-7}$
