# Supplementary material for: Mining the Human Phenome Using Allelic Scores That Index Biological Intermediates
Source: PLoS Genet. 2013 Oct 31;9(10):e1003919. doi: 10.1371/journal.pgen.1003919 (PMC3814299; doi:10.1371/journal.pgen.1003919)
Supplement: Table S3 — Association between case-control status in the WTCCC and an allelic score that proxies for LDL. (PDF) [file pgen.1003919.s012.pdf]

**Table S3. Association between case-control status in the WTCCC and an allelic score that proxies for LDL.** The left hand side of the table shows results for an allelic score consisting of all SNPs that meet a certain threshold ("All SNPs"), whilst the right side shows results for an allelic score consisting of all SNPs that meet a certain threshold with SNPs from known regions removed from its construction ("Complement"). SNPs have either been weighted according to their effect size from a previous meta-analysis ("Weighted"), or with each SNP getting an equal weighting ("Unweighted"). Results are shown for seventeen different p value thresholds and the number of SNPs that went into construction of the score for each threshold is listed also. Results are shown for seven different diseases. BD = Bipolar Disorder; CHD = Coronary Heart Disease; HT = Hypertension; CD = Crohn's Disease; RA = Rheumatoid Arthritis; T1D = Type 1 Diabetes; T2D = Type 2 Diabetes; Dir = Direction of effect; Pval = P value.

| All SNPs | | | | | Complement | | | | |
|---|---|---|---|---|---|---|---|---|---|
| | Weighted | | Unweighted | | | Weighted | | Unweighted | |
| | Dir | Pval | Dir | Pval | | Dir | Pval | Dir | Pval |
| $p<5x10^{-8}$ (251 SNPs) | | | | | $p<5x10^{-8}$ (0 SNPs) | | | | |
| BD | - | 0.88 | - | 0.70 | | + | NA | + | NA |
| CHD | + | $9.2x10^{-3}$ | + | 0.047 | | + | NA | + | NA |
| HT | - | 0.75 | - | 0.45 | | + | NA | + | NA |
| CD | - | 0.76 | - | 0.23 | | + | NA | + | NA |
| RA | - | 0.25 | - | 0.048 | | + | NA | + | NA |
| T1D | + | 0.58 | + | 0.82 | | + | NA | + | NA |
| T2D | - | 0.12 | - | 0.14 | | + | NA | + | NA |
| | | | | | | | | | |
| $p<5x10^{-7}$ (310 SNPs) | | | | | $p<5x10^{-7}$ (9 SNPs) | | | | |
| BD | - | 0.98 | - | 0.93 | | + | 0.44 | + | 0.42 |
| CHD | + | $7.5x10^{-3}$ | + | 0.034 | | + | 0.62 | + | 0.59 |
| HT | - | 0.73 | - | 0.45 | | - | 0.83 | - | 0.85 |
| CD | - | 0.79 | - | 0.30 | | + | 0.65 | + | 0.65 |
| RA | - | 0.26 | - | 0.076 | | + | 0.32 | + | 0.30 |
| T1D | + | 0.47 | + | 0.60 | | + | 0.089 | + | 0.089 |
| T2D | - | 0.11 | - | 0.13 | | - | 0.39 | - | 0.39 |

| | | | | | | | | | |
|---|---|---|---|---|---|---|---|---|---|
| | | | | | | | | | |
| $p<5x10^{-6}$ (417 SNPs) | | | | | $p<5x10^{-6}$ (48 SNPs) | | | | |
| BD | + | 0.98 | + | 0.97 | | + | 0.96 | + | 0.96 |
| CHD | + | $5.7x10^{-3}$ | + | 0.021 | | + | 0.86 | + | 0.85 |
| HT | - | 0.68 | - | 0.44 | | - | 0.99 | + | 0.95 |
| CD | - | 0.96 | - | 0.60 | | + | 0.071 | + | 0.067 |
| RA | - | 0.24 | - | 0.098 | | + | 0.62 | + | 0.63 |
| T1D | + | 0.70 | - | 0.95 | | + | 0.098 | + | 0.088 |
| T2D | - | 0.11 | - | 0.17 | | + | 0.66 | + | 0.51 |
| | | | | | | | | | |
| $p<5x10^{-5}$ (647 SNPs) | | | | | $p<5x10^{-5}$ (141 SNPs) | | | | |
| BD | - | 0.87 | - | 0.81 | | + | 0.93 | - | 0.99 |
| CHD | + | $6.4x10^{-3}$ | + | 0.023 | | + | 0.44 | + | 0.40 |
| HT | - | 0.34 | - | 0.11 | | - | 0.86 | - | 0.92 |
| CD | + | 0.84 | - | 0.99 | | + | 0.011 | + | 0.011 |
| RA | - | 0.21 | - | 0.12 | | + | 0.83 | + | 0.88 |
| T1D | - | 0.86 | - | 0.31 | | + | 0.081 | + | 0.13 |
| T2D | - | 0.22 | - | 0.57 | | + | 0.20 | + | 0.12 |
| | | | | | | | | | |
| $p<5x10^{-4}$ (1138 SNPs) | | | | | $p<5x10^{-4}$ (469 SNPs) | | | | |
| BD | - | 0.97 | - | 0.94 | | + | 0.89 | - | 0.94 |
| CHD | + | $1.5x10^{-3}$ | + | $3.9x10^{-3}$ | | + | 0.41 | + | 0.45 |
| HT | - | 0.48 | - | 0.37 | | + | 0.71 | + | 0.59 |
| CD | + | 0.45 | + | 0.40 | | + | 0.021 | + | 0.040 |
| RA | - | 0.38 | - | 0.44 | | + | 0.53 | + | 0.59 |
| T1D | - | 0.64 | - | 0.17 | | + | 0.25 | + | 0.45 |

| | | | | | | | | | |
|---|---|---|---|---|---|---|---|---|---|
| T2D | - | 0.60 | + | 0.62 | | + | 0.039 | + | 0.027 |
| | | | | | | | | | |
| $p<5x10^{-3}$ (3635 SNPs) | | | | | $p<5x10^{-3}$ (2602 SNPs) | | | | |
| BD | - | 0.30 | - | 0.077 | | - | 0.12 | - | 0.035 |
| CHD | + | $1.4x10^{-3}$ | + | $9.0x10^{-3}$ | | + | 0.66 | + | 0.77 |
| HT | - | 0.21 | - | 0.12 | | - | 0.21 | - | 0.16 |
| CD | + | 0.63 | + | 0.74 | | + | 0.40 | + | 0.64 |
| RA | - | 0.19 | - | 0.25 | | - | 0.79 | - | 0.87 |
| T1D | - | 0.011 | - | $1.0x10^{-4}$ | | - | 0.32 | - | 0.14 |
| T2D | - | 0.88 | + | 0.49 | | + | 0.17 | + | 0.22 |
| | | | | | | | | | |
| $p<5x10^{-2}$ (21431 SNPs) | | | | | $p<5x10^{-2}$ (19414 SNPs) | | | | |
| BD | - | 0.34 | - | 0.19 | | - | 0.45 | - | 0.30 |
| CHD | + | $1.2x10^{-4}$ | + | $1.6x10^{-3}$ | | + | 0.022 | + | 0.035 |
| HT | - | 0.062 | - | 0.024 | | - | 0.053 | - | 0.024 |
| CD | + | 0.83 | - | 0.87 | | + | 0.73 | - | 0.96 |
| RA | - | 0.30 | - | 0.37 | | - | 0.91 | - | 0.80 |
| T1D | - | 0.092 | - | 0.076 | | - | 0.87 | - | 0.74 |
| T2D | + | 0.31 | + | 0.086 | | + | 0.044 | + | 0.052 |
| | | | | | | | | | |
| $p<0.1$ (40201 SNPs) | | | | | $p<0.1$ (37522 SNPs) | | | | |
| BD | - | 0.15 | - | 0.071 | | - | 0.18 | - | 0.12 |
| CHD | + | $1.8x10^{-4}$ | + | $3.0x10^{-3}$ | | + | 0.018 | + | 0.031 |
| HT | - | 0.034 | - | 0.016 | | - | 0.035 | - | 0.021 |
| CD | + | 0.96 | - | 0.73 | | + | 0.94 | - | 0.76 |
| RA | - | 0.33 | - | 0.38 | | - | 0.80 | - | 0.67 |

| | | | | | | | | | |
|---|---|---|---|---|---|---|---|---|---|
| T1D | - | 0.026 | - | 0.018 | | - | 0.45 | - | 0.34 |
| T2D | + | 0.38 | + | 0.14 | | + | 0.091 | + | 0.098 |
| | | | | | | | | | |
| p<0.2 (77137 SNPs) | | | | | p<0.2 (73366 SNPs) | | | | |
| BD | - | 0.15 | - | 0.099 | | - | 0.18 | - | 0.15 |
| CHD | + | $2.1x10^{-4}$ | + | $5.4x10^{-3}$ | | + | 0.015 | + | 0.037 |
| HT | - | 0.031 | - | 0.018 | | - | 0.038 | - | 0.028 |
| CD | - | 0.96 | - | 0.58 | | - | 0.94 | - | 0.56 |
| RA | - | 0.44 | - | 0.46 | | - | 0.92 | - | 0.77 |
| T1D | - | 0.031 | - | 0.036 | | - | 0.43 | - | 0.35 |
| T2D | + | 0.40 | + | 0.20 | | + | 0.16 | + | 0.19 |
| | | | | | | | | | |
| p<0.3 (114252 SNPs) | | | | | p<0.3 (109551 SNPs) | | | | |
| BD | - | 0.10 | - | 0.075 | | - | 0.12 | - | 0.10 |
| CHD | + | $5.0x10^{-4}$ | + | 0.010 | | + | 0.028 | + | 0.062 |
| HT | - | 0.018 | - | 0.012 | | - | 0.018 | - | 0.014 |
| CD | - | 0.88 | - | 0.48 | | - | 0.85 | - | 0.45 |
| RA | - | 0.45 | - | 0.44 | | - | 0.86 | - | 0.64 |
| T1D | - | 0.049 | - | 0.11 | | - | 0.50 | - | 0.50 |
| T2D | + | 0.55 | + | 0.34 | | + | 0.33 | + | 0.40 |
| | | | | | | | | | |
| p<0.4 (151402 SNPs) | | | | | p<0.4 (145758 SNPs) | | | | |
| BD | - | 0.057 | - | 0.029 | | - | 0.067 | - | 0.043 |
| CHD | + | $5.9x10^{-4}$ | + | 0.013 | | + | 0.029 | + | 0.068 |
| HT | - | 0.012 | - | $6.0x10^{-3}$ | | - | 0.011 | - | $6.5x10^{-3}$ |
| CD | - | 0.76 | - | 0.30 | | - | 0.74 | - | 0.28 |

| | | | | | | | | | |
|---|---|---|---|---|---|---|---|---|---|
| RA | - | 0.37 | - | 0.33 | | - | 0.70 | - | 0.45 |
| T1D | - | 0.041 | - | 0.074 | | - | 0.41 | - | 0.31 |
| T2D | + | 0.64 | + | 0.46 | | + | 0.45 | + | 0.60 |
| | | | | | | | | | |
| p<0.5 (188909 SNPs) | | | | | p<0.5 (182382 SNPs) | | | | |
| BD | - | 0.064 | - | 0.057 | | - | 0.074 | - | 0.079 |
| CHD | + | $7.2x10^{-4}$ | + | 0.015 | | + | 0.029 | + | 0.066 |
| HT | - | 0.011 | - | $7.0x10^{-3}$ | | - | 0.011 | - | $7.8x10^{-3}$ |
| CD | - | 0.77 | - | 0.28 | | - | 0.74 | - | 0.26 |
| RA | - | 0.32 | - | 0.29 | | - | 0.60 | - | 0.37 |
| T1D | - | 0.035 | - | 0.062 | | - | 0.32 | - | 0.21 |
| T2D | + | 0.61 | + | 0.37 | | + | 0.44 | + | 0.50 |
| | | | | | | | | | |
| p<0.6 (226525 SNPs) | | | | | p<0.6 (219111 SNPs) | | | | |
| BD | - | 0.057 | - | 0.053 | | - | 0.067 | - | 0.078 |
| CHD | + | $1.1x10^{-3}$ | + | 0.032 | | + | 0.039 | + | 0.12 |
| HT | - | $9.1x10^{-3}$ | - | $6.0x10^{-3}$ | | - | $9.3x10^{-3}$ | - | $7.2x10^{-3}$ |
| CD | - | 0.74 | - | 0.23 | | - | 0.72 | - | 0.21 |
| RA | - | 0.32 | - | 0.37 | | - | 0.59 | - | 0.45 |
| T1D | - | 0.023 | - | 0.030 | | - | 0.24 | - | 0.11 |
| T2D | + | 0.64 | + | 0.41 | | + | 0.45 | + | 0.52 |
| | | | | | | | | | |
| p<0.7 (263835 SNPs) | | | | | p<0.7 (255616 SNPs) | | | | |
| BD | - | 0.051 | - | 0.057 | | - | 0.061 | - | 0.087 |
| CHD | + | $1.3x10^{-3}$ | + | 0.036 | | + | 0.044 | + | 0.12 |
| HT | - | $8.9x10^{-3}$ | - | $9.1x10^{-3}$ | | - | $9.5x10^{-3}$ | - | 0.012 |

| | | | | | | | | | |
|---|---|---|---|---|---|---|---|---|---|
| CD | - | 0.72 | - | 0.20 | | - | 0.70 | - | 0.19 |
| RA | - | 0.30 | - | 0.37 | | - | 0.56 | - | 0.46 |
| T1D | - | 0.019 | - | 0.029 | | - | 0.21 | - | 0.11 |
| T2D | + | 0.67 | + | 0.45 | | + | 0.49 | + | 0.56 |
| | | | | | | | | | |
| $p<0.8$ (301224 SNPs) | | | | | $p<0.8$ (292163 SNPs) | | | | |
| BD | - | 0.051 | - | 0.058 | | - | 0.060 | - | 0.085 |
| CHD | + | $1.4x10^{-3}$ | + | 0.045 | | + | 0.044 | + | 0.14 |
| HT | - | $9.9x10^{-3}$ | - | 0.013 | | - | 0.011 | - | 0.016 |
| CD | - | 0.70 | - | 0.14 | | - | 0.68 | - | 0.13 |
| RA | - | 0.29 | - | 0.30 | | - | 0.54 | - | 0.39 |
| T1D | - | 0.020 | - | 0.024 | | - | 0.21 | - | 0.092 |
| T2D | + | 0.66 | + | 0.44 | | + | 0.48 | + | 0.55 |
| | | | | | | | | | |
| $p<0.9$ (338229 SNPs) | | | | | $p<0.9$ (328349 SNPs) | | | | |
| BD | - | 0.047 | - | 0.047 | | - | 0.056 | - | 0.078 |
| CHD | + | $1.5x10^{-3}$ | + | 0.072 | | + | 0.046 | + | 0.19 |
| HT | - | 0.011 | - | 0.018 | | - | 0.012 | - | 0.024 |
| CD | - | 0.69 | - | 0.13 | | - | 0.68 | - | 0.13 |
| RA | - | 0.28 | - | 0.25 | | - | 0.53 | - | 0.35 |
| T1D | - | 0.019 | - | 0.016 | | - | 0.22 | - | 0.075 |
| T2D | + | 0.65 | + | 0.45 | | + | 0.47 | + | 0.55 |
| | | | | | | | | | |
| All (375112 SNPs) | | | | | All (364387 SNPs) | | | | |
| BD | - | 0.049 | - | 0.054 | | - | 0.059 | - | 0.086 |
| CHD | + | $1.7x10^{-3}$ | + | 0.094 | | + | 0.049 | + | 0.24 |

| | | | | | | | | | |
|---|---|---|---|---|---|---|---|---|---|
| HT | - | 0.011 | - | 0.025 | | - | 0.012 | - | 0.032 |
| CD | - | 0.73 | - | 0.23 | | - | 0.71 | - | 0.22 |
| RA | - | 0.26 | - | 0.18 | | - | 0.50 | - | 0.25 |
| T1D | - | 0.018 | - | 0.014 | | - | 0.20 | - | 0.059 |
| T2D | + | 0.66 | + | 0.54 | | + | 0.48 | + | 0.64 |
