# Supplementary material for: Mining the Human Phenome Using Allelic Scores That Index Biological Intermediates
Source: PLoS Genet. 2013 Oct 31;9(10):e1003919. doi: 10.1371/journal.pgen.1003919 (PMC3814299; doi:10.1371/journal.pgen.1003919)
Supplement: Table S4 — Association between case-control status in the WTCCC and an LD pruned allelic score that proxies for BMI. (PDF) [file pgen.1003919.s013.pdf]

Table S4. Association between case-control status in the WTCCC and an LD pruned allelic score that proxies for BMI. The left hand side of the table shows results for a weighted allelic score consisting of all SNPs that meet a certain threshold ("All SNPs"), whilst the right side shows results for a weighted allelic score that has been pruned for LD ("Thinned Set"). Results are shown for seventeen different p value thresholds and the number of SNPs that went into construction of the score for each threshold is listed also. Results are shown for seven different diseases. BD = Bipolar Disorder; CHD = Coronary Heart Disease; HT = Hypertension; CD = Crohn's Disease; RA = Rheumatoid Arthritis; T1D = Type 1 Diabetes; T2D = Type 2 Diabetes; Dir = Direction of effect; Pval = P value.

All SNPs			Thinned Set		
	Dir	Pval		Dir	Pval
$p<5x10^{-8}$ (158 SNPs)			$p<5x10^{-8}$ (30 SNPs)		
BD	-	0.62		-	0.63
CHD	+	0.17		+	0.27
HT	-	0.58		-	0.82
CD	+	0.90		+	0.80
RA	+	0.15		+	0.13
T1D	+	0.77		-	0.85
T2D	+	$4.3x10^{-7}$		+	$3.2x10^{-7}$
$p<5x10^{-7}$ (224 SNPs)			$p<5x10^{-7}$ (48 SNPs)		
BD	-	0.88		-	0.92
CHD	+	0.13		+	0.22
HT	-	0.63		-	0.87
CD	+	0.81		-	0.83
RA	+	0.15		+	0.24
T1D	+	0.75		-	0.64
T2D	+	$1.3x10^{-7}$		+	$3.9x10^{-7}$
$p<5x10^{-6}$			$p<5x10^{-6}$		

(398 SNPs)			(102 SNPs)		
BD	-	0.30		-	0.024
CHD	+	0.18		-	0.96
HT	-	0.53		-	0.10
CD	-	0.62		-	0.013
RA	+	0.18		-	0.65
T1D	+	0.86		-	0.27
T2D	+	$5.3x10^{-8}$		+	$2.3x10^{-5}$
$p<5x10^{-5}$<br>(861 SNPs)			$p<5x10^{-5}$<br>(222 SNPs)		
BD	-	0.20		-	0.012
CHD	+	0.24		-	0.35
HT	-	0.76		-	0.13
CD	-	0.50		-	0.031
RA	+	0.37		-	0.17
T1D	+	0.84		-	0.091
T2D	+	$3.4x10^{-8}$		+	$8.9x10^{-6}$
$p<5x10^{-4}$<br>(2366 SNPs)			$p<5x10^{-4}$<br>(670 SNPs)		
BD	-	0.063		-	0.013
CHD	+	0.45		-	0.50
HT	+	0.83		-	0.50
CD	-	0.67		-	0.82
RA	+	0.40		-	0.27
T1D	-	0.85		-	0.036
T2D	+	$3.9x10^{-9}$		+	$6.6x10^{-8}$

$p<5x10^{-3}$ (8402 SNPs)			$p<5x10^{-3}$ (2616 SNPs)		
BD	-	0.036		-	$5.9x10^{-3}$
CHD	+	0.36		-	0.49
HT	+	0.20		-	0.70
CD	+	0.46		+	0.96
RA	+	0.50		-	0.090
T1D	-	0.90		-	0.084
T2D	+	$1.3x10^{-13}$		+	$2.6x10^{-11}$
$p<5x10^{-2}$ (36921 SNPs)			$p<5x10^{-2}$ (12262 SNPs)		
BD	-	0.013		-	0.016
CHD	+	0.20		+	0.90
HT	+	0.58		-	0.23
CD	+	0.30		+	0.60
RA	+	0.78		-	0.040
T1D	+	0.98		-	0.31
T2D	+	$<2x10^{-16}$		+	$7.7x10^{-14}$
$p<0.1$ (60232 SNPs)			$p<0.1$ (19981 SNPs)		
BD	-	$8.6x10^{-3}$		-	0.045
CHD	+	0.28		+	0.86
HT	+	0.90		-	0.16
CD	+	0.34		+	0.32
RA	-	0.89		-	0.015
T1D	-	0.87		-	0.54
T2D	+	$3.3x10^{-16}$		+	$3.0x10^{-13}$

p<0.2 (101903 SNPs)			p<0.2 (33221 SNPs)		
BD	-	0.025		-	0.26
CHD	+	0.34		+	0.40
HT	-	0.98		-	0.26
CD	+	0.58		+	0.55
RA	-	0.58		-	0.048
T1D	-	0.97		-	0.82
T2D	+	$<2x10^{-16}$		+	$4.3x10^{-16}$
p<0.3 (140135 SNPs)			p<0.3 (44576 SNPs)		
BD	-	0.030		-	0.32
CHD	+	0.34		+	0.45
HT	-	0.99		-	0.26
CD	+	0.59		+	0.35
RA	-	0.37		-	0.039
T1D	+	0.96		-	0.87
T2D	+	$<2x10^{-16}$		+	<2e-16
p<0.4 (176024 SNPs)			p<0.4 (54639 SNPs)		
BD	-	0.046		-	0.39
CHD	+	0.33		+	0.47
HT	+	0.95		-	0.26
CD	+	0.73		+	0.36
RA	-	0.31		-	0.036
T1D	+	0.88		+	0.99

T2D	+	<2x10^{-16}		+	<2e-16
p<0.5 (210612 SNPs)			p<0.5 (63660 SNPs)		
BD	-	0.050		-	0.35
CHD	+	0.31		+	0.47
HT	-	0.87		-	0.10
CD	+	0.79		+	0.43
RA	-	0.22		-	0.018
T1D	+	0.85		-	0.98
T2D	+	<2x10^{-16}		+	9.0x10^{-16}
p<0.6 (244122 SNPs)			p<0.6 (71794 SNPs)		
BD	-	0.052		-	0.34
CHD	+	0.31		+	0.51
HT	-	0.82		-	0.10
CD	+	0.86		+	0.45
RA	-	0.20		-	0.017
T1D	+	0.81		+	0.97
T2D	+	<2x10^{-16}		+	1.8x10^{-15}
p<0.7 (277294 SNPs)			p<0.7 (78949 SNPs)		
BD	-	0.057		-	0.28
CHD	+	0.31		+	0.49
HT	-	0.79		-	0.11
CD	+	0.89		+	0.43
RA	-	0.19		-	0.018

T1D	+	0.88		-	0.92
T2D	+	$<2x10^{-16}$		+	$3.5x10^{-15}$
p<0.8<br>(309981 SNPs)			p<0.8<br>(85243 SNPs)		
BD	-	0.051		-	0.25
CHD	+	0.34		+	0.54
HT	-	0.78		-	0.11
CD	+	0.98		+	0.47
RA	-	0.18		-	0.016
T1D	+	0.95		-	0.85
T2D	+	$<2x10^{-16}$		+	$5.5x10^{-15}$
p<0.9<br>(342383 SNPs)			p<0.9<br>(90741 SNPs)		
BD	-	0.052		-	0.25
CHD	+	0.37		+	0.56
HT	-	0.76		-	0.11
CD	-	0.98		+	0.50
RA	-	0.18		-	0.016
T1D	+	0.97		-	0.82
T2D	+	$<2x10^{-16}$		+	$9.8x10^{-15}$
All<br>(392249 SNPs)			All<br>(95177 SNPs)		
BD	-	0.051		-	0.25
CHD	+	0.37		+	0.56
HT	-	0.76		-	0.11
CD	-	0.97		+	0.50

RA	-	0.18		-	0.016
T1D	+	0.97		-	0.82
T2D	+	$<2x10^{-16}$		+	$8.9x10^{-15}$
