# Supplementary material for: Mining the Human Phenome Using Allelic Scores That Index Biological Intermediates
Source: PLoS Genet. 2013 Oct 31;9(10):e1003919. doi: 10.1371/journal.pgen.1003919 (PMC3814299; doi:10.1371/journal.pgen.1003919)
Supplement: Table S5 — Association between case-control status in the WTCCC and an LD pruned allelic score that proxies for CRP. (PDF) [file pgen.1003919.s014.pdf]

Table S5. Association between case-control status in the WTCCC and an LD pruned allelic score that proxies for CRP. The left hand side of the table shows results for a weighted allelic score consisting of all SNPs that meet a certain threshold ("All SNPs"), whilst the right side shows results for a weighted allelic score that has been pruned for LD ("Thinned Set"). Results are shown for seventeen different p value thresholds and the number of SNPs that went into construction of the score for each threshold is listed also. Results are shown for seven different diseases. BD = Bipolar Disorder; CHD = Coronary Heart Disease; HT = Hypertension; CD = Crohn's Disease; RA = Rheumatoid Arthritis; T1D = Type 1 Diabetes; T2D = Type 2 Diabetes; Dir = Direction of effect; Pval = P value.

All SNPs			Thinned Set		
	Dir	Pval		Dir	Pval
$p<5x10^{-8}$ (152 SNPs)			$p<5x10^{-8}$ (40 SNPs)		
BD	+	0.11		+	0.060
CHD	+	0.80		-	0.71
HT	+	0.23		+	0.44
CD	+	0.051		+	$9.0x10^{-3}$
RA	+	0.028		+	0.13
T1D	+	0.15		+	0.18
T2D	+	0.50		+	0.23
$p<5x10^{-7}$ (184 SNPs)			$p<5x10^{-7}$ (46 SNPs)		
BD	+	0.086		+	0.041
CHD	+	0.63		-	0.89
HT	+	0.15		+	0.42
CD	+	0.031		+	$5.0x10^{-3}$
RA	+	0.023		+	0.11
T1D	+	0.080		+	0.16
T2D	+	0.32		+	0.15
$p<5x10^{-6}$			$p<5x10^{-6}$		

(262 SNPs)			(70 SNPs)		
BD	+	0.12		+	0.031
CHD	+	0.43		+	0.80
HT	+	0.17		+	0.31
CD	+	0.048		+	$5.7x10^{-3}$
RA	+	0.011		+	0.042
T1D	+	0.068		+	0.16
T2D	+	0.19		+	0.065
$p<5x10^{-5}$ (388 SNPs)			$p<5x10^{-5}$ (107 SNPs)		
BD	+	0.15		+	0.027
CHD	+	0.36		+	0.68
HT	+	0.21		+	0.29
CD	+	0.016		+	$2.9x10^{-3}$
RA	+	$3.0x10^{-3}$		+	$9.1x10^{-3}$
T1D	+	0.077		+	0.098
T2D	+	0.055		+	0.058
$p<5x10^{-4}$ (911 SNPs)			$p<5x10^{-4}$ (306 SNPs)		
BD	+	0.083		+	0.023
CHD	+	0.15		+	0.32
HT	+	0.085		+	0.33
CD	+	$4.3x10^{-3}$		+	$5.0x10^{-3}$
RA	+	$6.5x10^{-4}$		+	$2.7x10^{-3}$
T1D	+	0.056		+	0.035
T2D	+	$9.5x10^{-3}$		+	0.050

$p<5x10^{-3}$ (3798 SNPs)			$p<5x10^{-3}$ (1516 SNPs)		
BD	+	0.093		+	0.020
CHD	+	0.033		+	0.024
HT	+	0.073		+	0.094
CD	+	$2.2x10^{-3}$		+	0.016
RA	+	$7.6x10^{-4}$		+	$3.3x10^{-3}$
T1D	+	0.038		+	0.039
T2D	+	$1.4x10^{-3}$		+	$2.7x10^{-3}$
$p<5x10^{-2}$ (24159 SNPs)			$p<5x10^{-2}$ (9597 SNPs)		
BD	+	0.15		+	0.26
CHD	+	0.021		+	0.034
HT	+	0.12		+	0.40
CD	+	$8.3x10^{-4}$		+	0.012
RA	+	$8.4x10^{-3}$		+	0.056
T1D	+	0.046		+	0.081
T2D	+	$4.1x10^{-5}$		+	$2.4x10^{-3}$
$p<0.1$ (44677 SNPs)			$p<0.1$ (17090 SNPs)		
BD	+	0.12		+	0.23
CHD	+	$8.9x10^{-3}$		+	0.011
HT	+	0.13		+	0.33
CD	+	$3.0x10^{-4}$		+	$5.1x10^{-3}$
RA	+	0.014		+	0.10
T1D	+	0.023		+	0.067
T2D	+	$2.6x10^{-6}$		+	$1.8x10^{-4}$

p<0.2 (83740 SNPs)			p<0.2 (30165 SNPs)		
BD	+	0.14		+	0.31
CHD	+	$7.3x10^{-3}$		+	0.021
HT	+	0.18		+	0.44
CD	+	$2.3x10^{-4}$		+	$4.1x10^{-3}$
RA	+	0.083		+	0.33
T1D	+	0.012		+	0.12
T2D	+	$1.0x10^{-6}$		+	$3.9x10^{-5}$
p<0.3 (121099 SNPs)			p<0.3 (41759 SNPs)		
BD	+	0.25		+	0.65
CHD	+	0.018		+	0.049
HT	+	0.24		+	0.72
CD	+	$6.9x10^{-4}$		+	$9.3x10^{-3}$
RA	+	0.14		+	0.59
T1D	+	0.017		+	0.18
T2D	+	$8.6x10^{-7}$		+	$6.5x10^{-5}$
p<0.4 (157932 SNPs)			p<0.4 (52202 SNPs)		
BD	+	0.31		+	0.77
CHD	+	0.028		+	0.046
HT	+	0.25		+	0.67
CD	+	$6.0x10^{-4}$		+	$3.1x10^{-3}$
RA	+	0.17		+	0.78
T1D	+	0.021		+	0.097

T2D	+	6.0×10^{-7}		+	6.9×10^{-5}
p<0.5 (194853 SNPs)			p<0.5 (61963 SNPs)		
BD	+	0.36		+	0.80
CHD	+	0.028		+	0.046
HT	+	0.23		+	0.73
CD	+	5.0×10^{-4}		+	3.6×10^{-3}
RA	+	0.17		+	0.85
T1D	+	0.017		+	0.14
T2D	+	2.1×10^{-7}		+	6.3×10^{-5}
p<0.6 (231075 SNPs)			p<0.6 (70578 SNPs)		
BD	+	0.36		+	0.84
CHD	+	0.026		+	0.054
HT	+	0.22		+	0.73
CD	+	4.8×10^{-4}		+	5.1×10^{-3}
RA	+	0.18		+	0.91
T1D	+	0.019		+	0.13
T2D	+	1.4×10^{-7}		+	6.8×10^{-5}
p<0.7 (267164 SNPs)			p<0.7 (78187 SNPs)		
BD	+	0.36		+	0.82
CHD	+	0.028		+	0.047
HT	+	0.20		+	0.71
CD	+	4.0×10^{-4}		+	4.3×10^{-3}
RA	+	0.17		+	0.88

T1D	+	0.018		+	0.13
T2D	+	$8.5x10^{-8}$		+	$4.8x10^{-5}$
p<0.8 (303012 SNPs)			p<0.8 (85017 SNPs)		
BD	+	0.38		+	0.89
CHD	+	0.028		+	0.051
HT	+	0.20		+	0.77
CD	+	$3.4x10^{-4}$		+	$4.4x10^{-3}$
RA	+	0.17		+	0.93
T1D	+	0.019		+	0.16
T2D	+	$8.4x10^{-8}$		+	$7.4x10^{-5}$
p<0.9 (339088 SNPs)			p<0.9 (90632 SNPs)		
BD	+	0.37		+	0.86
CHD	+	0.028		+	0.051
HT	+	0.20		+	0.75
CD	+	$3.0x10^{-4}$		+	$4.1x10^{-3}$
RA	+	0.18		+	0.92
T1D	+	0.020		+	0.16
T2D	+	$8.2x10^{-8}$		+	$6.8x10^{-5}$
All (375099 SNPs)			All (95090 SNPs)		
BD	+	0.37		+	0.85
CHD	+	0.028		+	0.050
HT	+	0.20		+	0.75
CD	+	$2.9x10^{-4}$		+	$4.0x10^{-3}$

RA	+	0.17		+	0.91
T1D	+	0.020		+	0.15
T2D	+	$7.6x10^{-8}$		+	$6.4x10^{-5}$
