# Supplementary material for: Mining the Human Phenome Using Allelic Scores That Index Biological Intermediates
Source: PLoS Genet. 2013 Oct 31;9(10):e1003919. doi: 10.1371/journal.pgen.1003919 (PMC3814299; doi:10.1371/journal.pgen.1003919)
Supplement: Table S6 — Association between case-control status in the WTCCC and an LD pruned allelic score that proxies for LDLc. (PDF) [file pgen.1003919.s015.pdf]

Table S6. Association between case-control status in the WTCCC and an LD pruned allelic score that proxies for LDLc. The left hand side of the table shows results for a weighted allelic score consisting of all SNPs that meet a certain threshold ("All SNPs"), whilst the right side shows results for a weighted allelic score that has been pruned for LD ("Thinned Set"). Results are shown for seventeen different p value thresholds and the number of SNPs that went into construction of the score for each threshold is listed also. Results are shown for seven different diseases. BD = Bipolar Disorder; CHD = Coronary Heart Disease; HT = Hypertension; CD = Crohn's Disease; RA = Rheumatoid Arthritis; T1D = Type 1 Diabetes; T2D = Type 2 Diabetes; Dir = Direction of effect; Pval = P value.

	All SNPs			Thinned Set	
	Dir	Pval		Dir	Pval
$p<5x10^{-8}$ (251 SNPs)			$p<5x10^{-8}$ (72 SNPs)		
BD	-	0.88		-	0.32
CHD	+	$9.2x10^{-3}$		+	$2.3x10^{-8}$
HT	-	0.75		-	0.27
CD	-	0.76		-	0.37
RA	-	0.25		-	0.066
T1D	+	0.58		+	0.77
T2D	-	0.12		-	$7.7x10^{-3}$
$p<5x10^{-7}$ (310 SNPs)			$p<5x10^{-7}$ (89 SNPs)		
BD	-	0.98		-	0.46
CHD	+	$7.5x10^{-3}$		+	$2.5x10^{-9}$
HT	-	0.73		-	0.42
CD	-	0.79		-	0.48
RA	-	0.26		-	0.16
T1D	+	0.47		+	0.74
T2D	-	0.11		-	0.014
$p<5x10^{-6}$			$p<5x10^{-6}$		

(417 SNPs)			(121 SNPs)		
BD	+	0.98		-	0.64
CHD	+	$5.7x10^{-3}$		+	$2.8x10^{-8}$
HT	-	0.68		-	0.29
CD	-	0.96		-	0.81
RA	-	0.24		-	0.17
T1D	+	0.70		-	0.24
T2D	-	0.11		-	0.033
$p<5x10^{-5}$ (647 SNPs)			$p<5x10^{-5}$ (184 SNPs)		
BD	-	0.87		-	0.51
CHD	+	$6.4x10^{-3}$		+	$1.5x10^{-8}$
HT	-	0.34		-	0.19
CD	+	0.84		-	0.68
RA	-	0.21		-	0.083
T1D	-	0.86		-	0.13
T2D	-	0.22		-	0.087
$p<5x10^{-4}$ (1138 SNPs)			$p<5x10^{-4}$ (360 SNPs)		
BD	-	0.97		-	0.47
CHD	+	$1.5x10^{-3}$		+	$5.3x10^{-9}$
HT	-	0.48		-	0.23
CD	+	0.45		-	0.99
RA	-	0.38		-	0.18
T1D	-	0.64		-	0.13
T2D	-	0.60		-	0.28

$p<5x10^{-3}$ (3635 SNPs)			$p<5x10^{-3}$ (1349 SNPs)		
BD	-	0.30		-	0.21
CHD	+	$1.4x10^{-3}$		+	$8.9x10^{-8}$
HT	-	0.21		-	0.22
CD	+	0.63		+	0.66
RA	-	0.19		-	0.043
T1D	-	0.011		-	0.028
T2D	-	0.88		+	0.98
$p<5x10^{-2}$ (21431 SNPs)			$p<5x10^{-2}$ (8312 SNPs)		
BD	-	0.34		-	0.18
CHD	+	$1.2x10^{-4}$		+	$5.0x10^{-8}$
HT	-	0.062		-	0.10
CD	+	0.83		+	0.60
RA	-	0.30		-	0.24
T1D	-	0.092		-	0.54
T2D	+	0.31		+	0.42
p<0.1 (40201 SNPs)			p<0.1 (15147 SNPs)		
BD	-	0.15		-	0.14
CHD	+	$1.8x10^{-4}$		+	$8.2x10^{-8}$
HT	-	0.034		-	0.070
CD	+	0.96		+	0.73
RA	-	0.33		-	0.35
T1D	-	0.026		-	0.12
T2D	+	0.38		+	0.44

p<0.2 (77137 SNPs)			p<0.2 (27540 SNPs)		
BD	-	0.15		-	0.071
CHD	+	$2.1x10^{-4}$		+	$1.5x10^{-7}$
HT	-	0.031		-	0.17
CD	-	0.96		-	0.99
RA	-	0.44		-	0.48
T1D	-	0.031		-	0.15
T2D	+	0.40		+	0.43
p<0.3 (114252 SNPs)			p<0.3 (39181 SNPs)		
BD	-	0.10		-	0.067
CHD	+	$5.0x10^{-4}$		+	$5.1x10^{-7}$
HT	-	0.018		-	0.10
CD	-	0.88		-	0.73
RA	-	0.45		-	0.30
T1D	-	0.049		-	0.16
T2D	+	0.55		+	0.62
p<0.4 (151402 SNPs)			p<0.4 (49666 SNPs)		
BD	-	0.057		-	0.10
CHD	+	$5.9x10^{-4}$		+	$1.1x10^{-6}$
HT	-	0.012		-	0.091
CD	-	0.76		-	0.78
RA	-	0.37		-	0.27
T1D	-	0.041		-	0.13

T2D	+	0.64		+	0.66
p<0.5 (188909 SNPs)			p<0.5 (59533 SNPs)		
BD	-	0.064		-	0.071
CHD	+	7.2×10^{-4}		+	3.2×10^{-6}
HT	-	0.011		-	0.088
CD	-	0.77		-	0.75
RA	-	0.32		-	0.28
T1D	-	0.035		-	0.085
T2D	+	0.61		+	0.76
p<0.6 (226525 SNPs)			p<0.6 (68349 SNPs)		
BD	-	0.057		-	0.043
CHD	+	1.1×10^{-3}		+	7.3×10^{-6}
HT	-	9.1×10^{-3}		-	0.067
CD	-	0.74		-	0.73
RA	-	0.32		-	0.21
T1D	-	0.023		-	0.046
T2D	+	0.64		+	0.82
p<0.7 (263835 SNPs)			p<0.7 (76173 SNPs)		
BD	-	0.051		-	0.044
CHD	+	1.3×10^{-3}		+	5.2×10^{-6}
HT	-	8.9×10^{-3}		-	0.079
CD	-	0.72		-	0.82
RA	-	0.30		-	0.23

T1D	-	0.019		-	0.048
T2D	+	0.67		+	0.71
p<0.8 (301224 SNPs)			p<0.8 (83110 SNPs)		
BD	-	0.051		-	0.031
CHD	+	$1.4x10^{-3}$		+	$5.5x10^{-6}$
HT	-	$9.9x10^{-3}$		-	0.068
CD	-	0.70		-	0.74
RA	-	0.29		-	0.20
T1D	-	0.020		-	0.038
T2D	+	0.66		+	0.75
p<0.9 (338229 SNPs)			p<0.9 (88950 SNPs)		
BD	-	0.047		-	0.028
CHD	+	$1.5x10^{-3}$		+	$5.4x10^{-6}$
HT	-	0.011		-	0.075
CD	-	0.69		-	0.73
RA	-	0.28		-	0.20
T1D	-	0.019		-	0.038
T2D	+	0.65		+	0.71
All (375112 SNPs)			All (93626 SNPs)		
BD	-	0.049		-	0.025
CHD	+	$1.7x10^{-3}$		+	$5.9x10^{-6}$
HT	-	0.011		-	0.070
CD	-	0.73		-	0.72

RA	-	0.26		-	0.18
T1D	-	0.018		-	0.034
T2D	+	0.66		+	0.73
