# Supplementary material for: Mining the Human Phenome Using Allelic Scores That Index Biological Intermediates
Source: PLoS Genet. 2013 Oct 31;9(10):e1003919. doi: 10.1371/journal.pgen.1003919 (PMC3814299; doi:10.1371/journal.pgen.1003919)
Supplement: Table S8 — Known SNPs contributing to the calculation of BMI, LDLc, and CRP allelic scores in QIMR twins replication set. (PDF) [file pgen.1003919.s017.pdf]

Table S8. Known SNPs contributing to the calculation of BMI, LDLc, and CRP allelic scores in QIMR twins replication set. The first seven columns refer to results from published meta-analyses, whereas the last three columns refer to association in the QIMR twins replication set.

Variable	SNP	Chromosome	Position	Putative Gene	Effect Allele	Effect size	QIMR RQSR[3]	QIMR pvalue	QIMR RSQ[4]
BMI	rs2815752	1	72585028	*NEGR1*	A	0.13	0.9995	0.63	0.00%
BMI	rs1514175	1	74764232	*TNNI3K*	A	0.07	0.9752	0.91	0.00%
BMI	rs1555543	1	96717385	*PTBP2*	C	0.06	0.9973	0.73	0.00%
BMI	rs543874	1	176156103	*SEC16B*	G	0.22	0.9881	0.051	0.05%
BMI	rs2867125	2	612827	*TMEM18*	C	0.31	0.9997	$6.4\text{x}10^{-4}$	0.10%
BMI	rs713586	2	25011512	*RBJ/ADCY3/POMC*	C	0.14	0.9924	0.179	0.02%
BMI	rs887912	2	59156381	*FANCL*	T	0.1	0.9930	0.020	0.09%
BMI	rs2890652	2	142676401	*LRP1B*	C	0.09	0.9658	0.82	0.00%
BMI	rs13078807	3	85966840	*CADM2*	G	0.1	0.9530	0.17	0.02%
BMI	rs9816226	3	187317193	*ETV5*	T	0.14	0.9947	$9.7\text{x}10^{-3}$	0.12%
BMI	rs10938397	4	44877284	*GNPDA2*	G	0.18	0.8209	0.072	0.05%
BMI	rs13107325	4	103407732	*SLC39A8*	T	0.19	0.7212	0.25	0.00%
BMI	rs2112347	5	75050998	*FLJ35779/HMGCR*	T	0.1	0.9192	0.68	0.00%
BMI	rs4836133	5	124360002	*ZNF608*	A	0.07	0.8723	0.75	0.00%
BMI	rs206936	6	34410847	*HMGA1*	G	0.06	0.9972	0.54	0.00%
BMI	rs987237	6	50911009	*TFAP2B*	G	0.13	0.9993	0.17	0.02%
BMI	rs10968576	9	28404339	*LRRN6C*	G	0.11	0.9973	0.041	0.07%
BMI	rs4929949	11	8561169	*RPL27A*	C	0.06	0.9611	0.91	0.00%
BMI	rs10767664	11	27682562	*BDNF*	A	0.19	0.9965	0.53	0.00%
BMI	rs3817334	11	47607569	*MTCH2*	T	0.06	0.9961	0.56	0.00%
BMI	rs7138803	12	48533735	*FAIM2*	A	0.12	0.9961	0.55	0.00%
BMI	rs4771122	13	26918180	*MTIF3*	G	0.09	0.9277	0.026	0.08%
BMI	rs11847697	14	29584863	*PRKD1*	T	0.17	0.9443	0.19	0.02%

BMI	rs10150332	14	79006717	*NRXN3*	C	0.13	0.9942	0.16	0.02%
BMI	rs2241423	15	65873892	*MAP2K5*	G	0.13	0.9993	0.96	0.00%
BMI	rs12444979	16	19841101	*GPRC5B*	C	0.17	0.9955	0.18	0.01%
BMI	rs7359397	16	28793160	*SH2B1*	T	0.15	0.9982	0.83	0.00%
BMI	rs1558902	16	52361075	*FTO*	A	0.39	0.8688	$9.5x10^{-4}$	0.21%
BMI	rs571312	18	55990749	*MC4R*	A	0.23	0.9635	$8.0x10^{-4}$	0.21%
BMI	rs29941	19	39001372	*KCTD15*	G	0.06	0.9973	0.95	0.00%
BMI	rs2287019	19	50894012	*QPCTL/GIPR*	C	0.15	0.9542	0.049	0.06%
BMI	rs3810291	19	52260843	*TMEM160*	A	0.09	0.7790	0.042	0.07%
CRP	rs12037222	1	39837548	*PABPC4*	A	0.045	0.9752	0.23	0.02%
CRP	rs4420065	1	65934049	*LEPR*	C	0.09	0.9972	0.016	0.17%
CRP	rs4129267	1	152692888	*IL6R*	C	0.079	0.9993	0.11	0.06%
CRP	rs2794520	1	157945440	*CRP*	C	0.16	0.9984	$2.1x10^{-8}$	1.10%
CRP	rs12239046	1	245668218	*NLRP3*	C	0.047	0.8573	0.34	0.00%
CRP	rs1260326	2	27584444	*GCKR*	T	0.072	0.9958	0.26	0.00%
CRP	rs6734238	2	113557501	*IL1F10*	G	0.05	0.9908	0.25	0.01%
CRP	rs4705952	5	131867517	*IRF1*	G	0.042	0.8900	0.98	0.00%
CRP	rs6901250	6	117220718	*GPRC6A*	A	0.035	0.9784	0.63	0.00%
CRP	rs13233571	7	72609167	*BCL7B*	C	0.054	0.6752	0.17	0.03%
CRP	rs9987289	8	9220768	*PPP1R3B*	A	0.069	0.9852	0.31	0.00%
CRP	rs10745954	12	102007224	*ASCL1*	A	0.039	0.9772	0.80	0.00%
CRP	rs1183910	12	119905190	*HNF1A*	G	0.149	0.9349	$5.5x10^{-4}$	0.40%
CRP	rs340029	15	58682257	*RORA*	T	0.032	0.9988	0.36	0.00%
CRP	rs10521222	16	49716211	*SALL1*	C	0.104	0.9855	0.28	0.00%
CRP	rs2847281	18	12811593	*PTPN2*	A	0.031	0.9907	0.65	0.00%
CRP	rs4420638	19	50114786	*APOC1*	A	0.236	0.5558	$4.1x10^{-7}$	0.89%
CRP	rs1800961	20	42475778	*HNF4A*	C	0.088	0.2575	0.042	0.11%

LDLc	rs12027135	1	25648320	*LDLRAP1*	T	NA	0.9703	0.88	0.00%
LDLc	rs2479409	1	55277238	*PCSK9*	G	NA	0.5645	0.33	0.00%
LDLc	rs2131925	1	62798530	*ANGPTL3*	T	NA	0.9975	0.80	0.00%
LDLc	rs629301	1	109619829	*SORT1*	T	NA	0.9978	$2.6x10^{-7}$	0.97%
LDLc	rs2642442	1	219040186	*MOSC1*	T	NA	0.8561	0.38	0.00%
LDLc	rs514230	1	232925220	*IRF2BP2*	T	NA	0.9619	0.019	0.17%
LDLc	rs1367117	2	21117405	*APOB*	A	NA	0.8193	$6.2x10^{-8}$	1.07%
LDLc	rs4299376	2	43926080	*ABCG5/8*	G	NA	0.8800	0.14	0.05%
LDLc	rs12916	5	74692295	*HMGCR*	C	NA	0.9536	$9.4x10^{-4}$	0.38%
LDLc	rs6882076	5	156322875	*TIMD4*	C	NA	0.9540	0.14	0.05%
LDLc	rs3757354	6	16235386	*MYLIP*	C	NA	0.9977	0.46	0.00%
LDLc	rs1800562	6	26201120	*HFE*	G	NA	0.9985	0.64	0.00%
LDLc	rs3177928[1]	6	32520413	*HLA*	A	NA	0.9967	0.26	0.01%
LDLc	rs9488822	6	116419586	*FRK*	A	NA	0.9270	0.99	0.00%
LDLc	rs1564348	6	160498850	*LPA*	C	NA	0.9973	0.47	0.00%
LDLc	rs12670798	7	21573877	*DNAH11*	C	NA	0.9969	0.41	0.00%
LDLc	rs2072183	7	44545705	*NPC1L1*	C	NA	0.5197	0.79	0.00%
LDLc	rs9987289	8	9220768	*PPP1R3B*	G	NA	0.9852	0.37	0.00%
LDLc	rs2081687	8	59551119	*CYP7A1*	T	NA	0.9782	0.78	0.00%
LDLc	rs2954029	8	126560154	*TRIB1*	A	NA	0.9851	0.19	0.03%
LDLc	rs11136341	8	145115531	*PLEC1*	G	NA	0.7574	0.18	0.03%
LDLc	rs9411489[2]	9	135144821	*ABO*	T	NA	NA	NA	NA
LDLc	rs2255141	10	113923876	*GPAM*	A	NA	0.9971	0.10	0.07%
LDLc	rs174546	11	61326406	*FADS1-2-3*	C	NA	0.9997	$3.6x10^{-3}$	0.28%
LDLc	rs964184	11	116154127	*APOA1*	G	NA	0.9594	0.37	0.00%
LDLc	rs11220462	11	125749162	*ST3GAL4*	A	NA	0.9651	0.18	0.03%
LDLc	rs11065987	12	110556807	*BRAP*	A	NA	0.9373	0.34	0.00%
LDLc	rs1169288	12	119901033	*HNF1A*	C	NA	0.9483	0.094	0.07%
LDLc	rs8017377	14	23953727	*NYNRIN*	A	NA	0.9941	0.15	0.04%

LDLc	rs3764261	16	55550825	*CETP*	C	NA	0.9960	7.3×10^{-3}	0.23%
LDLc	rs2000999	16	70665594	*HPR*	A	NA	0.9025	0.22	0.02%
LDLc	rs7206971	17	42780114	*OSBPL7*	A	NA	0.9896	0.14	0.05%
LDLc	rs6511720	19	11063306	*LDLR*	G	NA	0.9285	8.7×10^{-4}	0.38%
LDLc	rs10401969	19	19268718	*CILP2*	T	NA	0.8310	2.0×10^{-3}	0.32%
LDLc	rs4420638	19	50114786	*APOE*	G	NA	0.5558	1.9×10^{-5}	0.66%
LDLc	rs2902940	20	38524901	*MAFB*	A	NA	0.9855	0.66	0.00%
LDLc	rs6029526	20	39106032	*TOP1*	A	NA	0.9808	0.48	0.00%

[1] Variants +/- 2 MB around this region were excluded from the calculations excluding known regions because of the high linkage disequilibrium in this part of the genome

[2] This SNP could not be imputed and so did not contribute to the allelic score for LDLc consisting of known variants only, but variants +/- 1 MB around it were still excluded from the calculations involving excluding known regions

[3] RSQR imputation accuracy as calculated by MACH in the QIMR twins cohort

[4] Proportion of variance explained (adjusted R^2) in the QIMR twins cohort
