# Supplementary material for: Mining the Human Phenome Using Allelic Scores That Index Biological Intermediates
Source: PLoS Genet. 2013 Oct 31;9(10):e1003919. doi: 10.1371/journal.pgen.1003919 (PMC3814299; doi:10.1371/journal.pgen.1003919)
Supplement: Table S9 — Performance of BMI SNPs in the CRP meta-analysis. (PDF) [file pgen.1003919.s018.pdf]

Table S9. Performance of BMI SNPs in the CRP meta-analysis

BMI SNP	Chromosome	Gene	P value in CRP Meta-analysis
rs2815752	1	*NEGR1*	0.00037
rs543874	1	*SEC16B*	0.011
rs1514175	1	*TNNI3K*	0.0087
rs1555543	1	*PTBP2*	0.58
rs2867125	2	*TMEM18*	$5.0x10^{-6}$
rs713586	2	*RBJ/ADCY3/POMC*	0.072
rs887912	2	*FANCL*	0.036
rs2890652	2	*LRP1B*	0.26
rs13078807	3	*CADM2*	0.35
rs9816226	3	*ETV5*	0.15
rs13107325	4	*SLC39A8*	0.44
rs10938397	4	*GNPDA2*	0.39
rs2112347	5	*FLJ35779/HMGCR*	0.43
rs4836133	5	*ZNF608*	0.040
rs987237	6	*TFAP2B*	0.0018
rs206936	6	*HMGA1*	0.65
rs10968576	9	*LRRN6C*	0.012
rs3817334	11	*MTCH2*	0.076
rs10767664	11	*BDNF*	0.38
rs4929949	11	*RPL27A*	0.90
rs7138803	12	*FAIM2*	0.12
rs4771122	13	*MTIF3*	0.10
rs10150332	14	*NRXN3*	0.013
rs11847697	14	*PRKD1*	0.00060
rs2241423	15	*MAP2K5*	0.37
rs7359397	16	*SH2B1*	0.56
rs12444979	16	*GPRC5B*	0.40
rs1558902	16	*FTO*	$2.2x10^{-6}$
rs571312	18	*MC4R*	$3.8x10^{-5}$

rs29941	19	*KCTD15*	0.22
rs2287019	19	*QPCTL/GIPR*	0.19
rs3810291	19	*TMEM160*	0.0091
